# Complete positivity and distance-avoiding sets

**DOI:** 10.1007/s10107-020-01562-6

**Published:** 2020-09-16

**Authors:** Evan DeCorte, Fernando Mário de Oliveira Filho, Frank Vallentin

**Affiliations:** 1 Mathematics and Statistics, McGill University, 805 Sherbrooke W., Montreal, QC H3A 0B9 Canada; 2 Delft Institute of Applied Mathematics, Delft University of Technology, Van Mourik Broekmanweg 6, 2628 XE Delft, The Netherlands; 3 Mathematisches Institut, Universität zu Köln, Weyertal 86–90, 50931 Köln, Germany

**Keywords:** Hadwiger-Nelson problem, Chromatic number of Euclidean space, Semidefinite programming, Copositive programming, Harmonic analysis, 46N10, 52C10, 51K99, 90C22, 90C34

## Abstract

We introduce the cone of completely positive functions, a subset of the cone of positive-type functions, and use it to fully characterize maximum-density distance-avoiding sets as the optimal solutions of a convex optimization problem. As a consequence of this characterization, it is possible to reprove and improve many results concerning distance-avoiding sets on the sphere and in Euclidean space.

## Introduction

The two prototypical geometrical problems considered in this paper are:

1. What is the maximum surface measure $m_0(S^{n-1})$ that a subset of the unit sphere $S^{n-1} = \{\, x \in {\mathbb {R}}^n : \Vert x\Vert = 1\,\}$ can have if it does not contain pairs of orthogonal vectors?
2. What is the maximum density $m_1({\mathbb {R}}^n)$ that a subset of ${\mathbb {R}}^n$ can have if it does not contain pairs of points at distance 1?

Problem (P1) was posed by Witsenhausen [48]. Two antipodal open spherical caps of radius $\pi /4$ form a subset of $S^{n-1}$ with no pairs of orthogonal vectors, and Kalai [20, Conjecture 2.8] conjectured that this construction is optimal, that is, that it attains $m_0(S^{n-1})$; this conjecture remains open for all $n \ge 2$. Problem (P1) will be considered in depth in Sect. 8, where many upper bounds for $m_0(S^{n-1})$ will be improved.

Problem (P2) figures in Moser’s collection of problems [32] and was popularized by Erdős, who conjectured that $m_1({\mathbb {R}}^2) < 1/4$ (cf. Székely [45]); this conjecture is still open. A long-standing conjecture of L. Moser (cf. Conjecture 1 in Larman and Rogers [26]), related to Erdős’s conjecture, would imply that $m_1({\mathbb {R}}^n) \le 1/2^n$ for all $n \ge 2$. Moser’s conjecture asserts that the maximum measure of a subset of the unit ball having no pairs of points at distance 1 is at most $1/2^n$ times the measure of the unit ball; it has recently been shown to be false [34]: the behavior of subsets of the unit ball that avoid distance 1 resembles Kalai’s double cap conjecture. Problem (P2) will be considered in detail in Sect. 9, where upper bounds for $m_1({\mathbb {R}}^n)$ will be improved.

Bachoc et al. [1] proposed an upper bound for $m_0(S^{n-1})$ similar to the linear programming bound of Delsarte et al. [10] for the maximum cardinality of spherical codes. Recall that a continuous function $f:[-1,1] \rightarrow {\mathbb {R}}$ is of *positive type for* $S^{n-1}$ if for every finite set $U \subseteq S^{n-1}$ the matrix $\bigl (f(x \cdot y)\bigr )_{x,y\in U}$ is positive semidefinite. Bachoc, Nebe, Oliveira, and Vallentin showed that the optimal value of the infinite-dimensional optimization problem

1 $$\begin{aligned} \begin{array}{r@{\ }l@{\quad }l} \text {maximize}&{}\int _{S^{n-1}} \int _{S^{n-1}} f(x\cdot y)\, d\omega (y) d\omega (x)\\ &{}f(1) = \omega (S^{n-1})^{-1},\\ &{}f(0) = 0,\\ &{}f:[-1,1]\rightarrow {\mathbb {R}}\text { is continuous and of positive type for}~S^{n-1} \end{array} \end{aligned}$$

is an upper bound for $m_0(S^{n-1})$. Here, $\omega$ is the surface measure on $S^{n-1}$.

Later, Oliveira and Vallentin [36] proposed an upper bound for $m_1({\mathbb {R}}^n)$ similar to the linear programming bound of Cohn and Elkies [7] for the maximum density of a sphere packing in ${\mathbb {R}}^n$; the Cohn–Elkies bound has recently been used to solve the sphere-packing problem in dimensions 8 and 24 [8, 46]. Recall that a continuous function $f:{\mathbb {R}}^n \rightarrow {\mathbb {R}}$ is of *positive type* if for every finite set $U \subseteq {\mathbb {R}}^n$ the matrix $\bigl (f(x-y)\bigr )_{x,y \in U}$ is positive semidefinite. Oliveira and Vallentin showed that the optimal value of the infinite-dimensional optimization problem

2 $$\begin{aligned} \begin{array}{r@{\ }l@{\quad }l} \text {maximize}&{}M(f)\\ &{}f(0)=1,\\ &{}f(x)=0\quad \text {if}~\Vert x\Vert = 1,\\ &{}f:{\mathbb {R}}^n \rightarrow {\mathbb {R}}\text { is continuous and of positive type} \end{array} \end{aligned}$$

is an upper bound for $m_1({\mathbb {R}}^n)$. Here, *M*(*f*) is the *mean value* of *f*, defined as

$$\begin{aligned} M(f) = \lim _{T \rightarrow \infty } \frac{1}{{{\,\mathrm{vol}\,}}[-T,T]^n} \int _{[-T,T]^n} f(x)\, dx. \end{aligned}$$

An explicit characterization of functions of positive type for $S^{n-1}$ is given by Schoenberg’s theorem [40]. Likewise, functions of positive type on ${\mathbb {R}}^n$ are characterized by Bochner’s theorem [38, Theorem IX.9]. Using these characterizations, it is possible to rewrite and simplify problems (1) and (2), which become infinite-dimensional linear programs. It then becomes possible to solve these problems by computer or even analytically; in this way, one obtains upper bounds for the geometrical parameters $m_0(S^{n-1})$ and $m_1({\mathbb {R}}^n)$. Both optimization problems above can also be strengthened by the addition of extra constraints. The best bounds for both geometrical parameters, in several dimensions, were obtained through strengthenings of the optimization problems above; see Sects. 8 and 9.

A symmetric matrix $A \in {\mathbb {R}}^{n \times n}$ is *completely positive* if it is a conic combination of rank-one, symmetric, and nonnegative matrices, that is, if there are nonnegative vectors $f_1$, ..., $f_k \in {\mathbb {R}}^n$ such that

$$\begin{aligned} A = f_1 \otimes f_1^* + \cdots + f_k \otimes f_k^*. \end{aligned}$$

The set of all completely positive matrices is a closed and convex cone of symmetric matrices that is strictly contained in the cone of positive-semidefinite matrices. Completely positive matrices are the main object of study in this paper.

A continuous function $f:[-1, 1] \rightarrow {\mathbb {R}}$ is of *completely positive type for* $S^{n-1}$ if for every finite set $U \subseteq S^{n-1}$ the matrix $\bigl (f(x \cdot y)\bigr )_{x,y \in U}$ is completely positive. Analogously, a continuous function $f :{\mathbb {R}}^n \rightarrow {\mathbb {R}}$ is of *completely positive type* if for every $U \subseteq {\mathbb {R}}^n$ the matrix $\bigl (f(x - y)\bigr )_{x,y \in U}$ is completely positive. Notice that functions of completely positive type are functions of positive type, but not every function of positive type is of completely positive type.

The central result of this paper is that, by considering functions of completely positive type instead of functions of positive type, one fully characterizes the geometrical parameters in (P1) and (P2).

### Theorem 1.1

If in (1) we require *f* to be of completely positive type, then the optimal value of the problem is exactly $m_0(S^{n-1})$. Similarly, if in (2) we require *f* to be of completely positive type, then the optimal value is exactly $m_1({\mathbb {R}}^n)$.

The significance of this result is twofold.

First, it gives us a source of constraints that can be added to (1) or (2) and asserts that this source is complete, that is, that the constraints are sufficient for us to obtain the exact parameters. Namely, for every finite set $U \subseteq S^{n-1}$ we can add to (1) the constraint that $\bigl (f(x \cdot y)\bigr )_{x, y \in U}$ has to be completely positive, and similarly for (2). All strengthenings of problems (1) and (2) considered so far in the literature have used such constraints. In this paper, by systematically using them, we are able to improve many of the known upper bounds for $m_0(S^{n-1})$ and $m_1({\mathbb {R}}^n)$; see Table 1 in Sect. 8 and Table 2 in Sect. 9.

**Table 1:** New upper bounds for the independence ratio of $G(S^{n-1}, \{\pi /2\})$. Next to each bound is the number of ${{\,\mathrm{BQP}\,}}(U)$-constraints used to obtain it. The lower bounds come from two opposite spherical caps. The bound for $n = 3$ improves on a previous bound of 0.308 by Zhao (personal communication); the bounds for $n \ge 4$ improve on Witsenhausen’s bound [48] of 1/*n*

*n*	Upper bound	Lower bound	# Extra constraints
3	0.30153	0.2929	11
4	0.21676	0.1817	2
5	0.16765	0.1161	1
6	0.13382	0.0756	3
7	0.11739	0.0498	2
8	0.09981	0.0331	2

**Table 2:** The bounds for $n = 3$ are due to Oliveira and Vallentin [36]; all other bounds are due to Bachoc et al. [2]. The graphs used for the subgraph constraints are indicated in the last column; they are the same ones used by Bachoc, Passuello, and Thiery (ibid., Table 2), except for the 8-simplex, which is the regular simplex of side-length 1 in ${\mathbb {R}}^8$

	Upper bound for $\alpha _{{\bar{\delta }}}$		Lower bound for $\chi _{\mathrm {m}}$		
*n*	Previous	New	Previous	New	Graphs used
3	0.1645090	0.1532996	7	7	None
4	0.1000620	0.0985701	10	11	600-cell
5	0.0677778	0.0624485	15	17	600-cell
6	0.0478444	0.0450325	21	23	600-cell
7	0.0276502	0.0260782	37	39	$E_8$ kissing
8	0.0195941	0.0190945	52	53	$E_8$ and 8-simplex

Second, the characterizations of $m_0(S^{n-1})$ and $m_1({\mathbb {R}}^n)$ in terms of convex optimization problems, even computationally difficult ones, is good enough to allow us to derive some interesting theoretical results through analytical methods. For instance, denote by $m_{d_1, \dots , d_N}({\mathbb {R}}^n)$ the maximum density that a Lebesgue-measurable set $I \subseteq {\mathbb {R}}^n$ can have if it is such that $\Vert x-y\Vert \notin \{d_1, \ldots , d_N\}$ for all distinct *x*, $y \in I$. Bukh [6] showed, unifying results by Furstenberg et al. [17], Bourgain [5], Falconer [14], and Falconer and Marstrand [13], that, as the distances $d_1$, ..., $d_N$ space out, so does $m_{d_1, \ldots , d_N}({\mathbb {R}}^n)$ approach $(m_1({\mathbb {R}}^n))^N$. This precise asymptotic result can be recovered from (2) by using functions of completely positive type in a systematic way that can provide precise analytic results. Another result of Bukh (ibid.) that can be proved using this approach is the Turing-machine computability of $m_1({\mathbb {R}}^n)$. Using our convex formulation one can in principle extend this computability result to distance-avoiding sets in other geometric spaces.

### Outline of the paper

The main theorem proved in this paper is Theorem 5.1, from which Theorem 1.1 follows. Theorem 5.1 is stated in terms of graphs on topological spaces and is much more general than Theorem 1.1. It has a rather technical statement, but it is in fact a natural extension of a well-known result in combinatorial optimization, namely that the independence number of a graph is the optimal value of a convex optimization problem over the cone of completely positive matrices. This connection is the main thread of this paper; it will be clarified in Sect. 3.

In Sect. 2 we will see how geometrical parameters such as $m_0(S^{n-1})$ and $m_1({\mathbb {R}}^n)$ can be modeled as the independence number of certain graphs defined over topological spaces such as the sphere. In Sect. 3 this will allow us to extend the completely positive formulation for the independence number from finite graphs to these topological graphs; this extension will rely on the introduction of the cone of completely positive operators on a Hilbert space. A study of these operators, carried out in Sect. 4, will then allow us to prove Theorem 5.1 in Sect. 5 and extend it from compact spaces to ${\mathbb {R}}^n$ in Sect. 6. In Sects. 7, 8, and 9 we will see how to use Theorem 5.1 to obtain better bounds for $m_0(S^{n-1})$ and $m_1({\mathbb {R}}^n)$; these sections will be focused on computational techniques. We close in Sect. 10 by seeing how Theorem 5.1 can be used to prove Bukh’s results [6] concerning sets avoiding many distances and the computability of $m_1({\mathbb {R}}^n)$.

### Notation

All graphs considered have no loops nor parallel edges. Often, the edge set of a graph $G = (V, E)$ is also seen as a symmetric subset of $V \times V$. In this case, *x*, $y \in V$ are adjacent if and only if (*x*, *y*), $(y, x) \in E$. A graph $G = (V, E)$ is a *topological graph* if *V* is a topological space; topological properties of *E* (e.g., closedness, compactness) always refer to *E* as a subset of $V \times V$.

If *V* is a metric space with metric *d*, then for $x \in V$ and $\delta > 0$ we denote by

$$\begin{aligned} B(x, \delta ) = \{\, y \in V : d(y, x) < \delta \,\} \end{aligned}$$

the open ball with center *x* and radius $\delta$. The topological closure of a set *X* is denoted by ${{\,\mathrm{cl}\,}}X$. The term “neighborhood” always means “open neighborhood”, though the distinction is never really relevant.

The Euclidean inner product on ${\mathbb {R}}^n$ is denoted by $x\cdot y = x_1 y_1 + \cdots + x_n y_n$ for *x*, $y \in {\mathbb {R}}^n$. The $(n-1)$-dimensional unit sphere is $S^{n-1} = \{\, x \in {\mathbb {R}}^n : \Vert x\Vert = 1\, \}$.

All functions considered are real valued unless otherwise noted. If *V* is a measure space with measure $\omega$, then the inner product of *f*, $g \in L^2(V)$ is

$$\begin{aligned} (f, g) = \int _V f(x) g(x)\, d\omega (x). \end{aligned}$$

The inner product of kernels *A*, $B \in L^2(V \times V)$ is

$$\begin{aligned} \langle A, B \rangle = \int _V \int _V A(x, y) B(x, y)\, d\omega (y) d\omega (x). \end{aligned}$$

When *V* is finite and $\omega$ is the counting measure, then $\langle A, B\rangle$ is the trace inner product. If $f \in L^2(V)$, then $f \otimes f^*$ denotes the kernel $(x, y) \mapsto f(x) f(y)$.

Denote by $L_\mathrm {sym}^2(V \times V)$ the space of all kernels that are symmetric, that is, self adjoint as operators. Note that $A \in L_\mathrm {sym}^2(V \times V)$ if and only if $A \in L^2(V \times V)$ and $A(x, y) = A(y, x)$ almost everywhere. A symmetric kernel *A* is *positive* if for all $f \in L^2(V)$ we have

$$\begin{aligned} \int _V \int _V A(x, y) f(x) f(y)\, dydx \ge 0. \end{aligned}$$

## Locally independent graphs

Let $G = (V, E)$ be a graph (without loops and parallel edges). A set $I \subseteq V$ is *independent* if it does not contain pairs of adjacent vertices, that is, if for all *x*, $y \in I$ we have $(x, y) \notin E$. The *independence number* of *G*, denoted by $\alpha (G)$, is the maximum cardinality of an independent set in *G*. The problem of computing the independence number of a finite graph figures, as the complementary maximum-clique problem, in Karp’s original list of 21 NP-hard problems [21].

To model the geometrical parameters $m_0(S^{n-1})$ and $m_1({\mathbb {R}}^n)$ as the independence number of some graph, we will have to extend the concept of independence number from finite to infinite graphs. Then the nature of both the vertex and edge sets plays a role; this can be best seen considering a few examples.

Let *V* be a metric space with metric *d* and take $D \subseteq (0, \infty )$. The *D* *-distance graph* on *V* is the graph *G*(*V*, *D*) whose vertex set is *V* and in which vertices *x*, *y* are adjacent if $d(x, y) \in D$. Independent sets in *G*(*V*, *D*) are sometimes called *D* *-avoiding sets*. Let us consider a few concrete choices for *V* and *D*, corresponding to central problems in discrete geometry.

(i) *The kissing number problem:* $V = S^{n-1}$ *and* $D = (0, \pi / 3)$. Here we consider the metric $d(x, y) = \arccos x\cdot y$. In this case, all independent sets in *G*(*V*, *D*) are finite; even more, the independence number is finite. The independent sets in *G*(*V*, *D*) are exactly the contact points of kissing configurations in ${\mathbb {R}}^n$, so $\alpha (G(V, D))$ is the kissing number of ${\mathbb {R}}^n$.
(ii) *Witsenhausen’s problem (P1):* $V = S^{n-1}$ *and* $D = \{\pi /2\}$. Again we consider the metric $d(x, y) = \arccos x\cdot y$. An independent set in *G*(*V*, *D*) is a set without pairs of orthogonal vectors. These sets can be infinite and even have positive surface measure, so $\alpha (G(V, D)) = \infty$. The right concept in this case is the *measurable independence number*
  $$\begin{aligned} \alpha _\omega (G(V, D)) = \sup \{\, \omega (I) : I \subseteq V \text { is measurable and independent}\,\}, \end{aligned}$$
  where $\omega$ is the surface measure on the sphere. Then $\alpha _\omega (G(V, D)) = m_0(S^{n-1})$.
(iii) *The sphere-packing problem:* $V = {\mathbb {R}}^n$ *and* $D = (0, 1)$. Here we consider the Euclidean metric. The independent sets in *G*(*V*, *D*) are the sets of centers of spheres in a packing of spheres of radius 1/2 in ${\mathbb {R}}^n$. So independent sets in *G*(*V*, *D*) can be infinite but are always discrete, hence $\alpha (G(V, D)) = \infty$ while independent sets always have Lebesgue measure 0. A better definition of independence number in this case would be the center density of the corresponding packing, that is, the average number of points per unit volume.
(iv) *Measurable one-avoiding sets (P2):* $V = {\mathbb {R}}^n$ *and* $D = \{1\}$. In this case, *G*(*V*, *D*) is called the unit-distance graph of ${\mathbb {R}}^n$. Independent sets in this graph can be infinite and even have infinite Lebesgue measure, hence $\alpha (G(V, D)) = \infty$. So the right notion of independence number is the density of a set, informally the fraction of space it covers. We will formally define the independence density $\alpha _{{\bar{\delta }}}(G(V , D)) = m_1({\mathbb {R}}^n)$ in Sect. 6.

In the first two examples above, the vertex set is compact. In (i), there is $\delta > 0$ such that $(0, \delta ) \subseteq D$. Then every point has a neighborhood that is a clique (that is, a set of pairwise adjacent vertices), and this implies that all independent sets are discrete and hence finite, given the compactness of *V*. In (ii), 0 is isolated from *D*. Then every point has an independent neighborhood and there are independent sets of positive measure.

In the last two examples, the vertex set is not compact. In (iii), again there is $\delta > 0$ such that $(0, \delta ) \subseteq D$, and this implies that all independent sets are discrete, though since *V* is not compact they can be infinite. In (iv), 0 is again isolated from *D*, hence there are independent sets of positive measure and even infinite measure, given that *V* is not compact.

We have therefore two things at play. First, compactness of the vertex set. Second, the nature of the edge set, which in the examples above depends on 0 being isolated from *D* or not.

In this paper, the focus rests on graphs with compact vertex sets, though the not compact case of ${\mathbb {R}}^n$ can be handled by seeing ${\mathbb {R}}^n$ as a limit of tori (see Sect. 6 below). As for the edge set, we consider graphs like the ones in examples (ii) and (iv).

The graphs in examples (i) and (iii) are *topological packing graphs*, a concept introduced by de Laat and Vallentin [25]. These are topological graphs in which every finite clique is a subset of an open clique. In particular, every vertex has a neighborhood that is a clique. Here and in the remainder of the paper we consider locally independent graphs, which are in a sense the complements of topological packing graphs.

### Definition 2.1

A topological graph is *locally independent* if every compact independent set in it is a subset of an open independent set.

In particular, every vertex of a locally independent graph has an independent neighborhood. The graphs in examples (ii) and (iv) are locally independent, as follows from the following theorem.

### Theorem 2.2

If $G = (V, E)$ is a topological graph, if *V* is metrizable, and if *E* is closed, then *G* is locally independent.

### Proof

Let *d* be a metric that induces the topology on *V*. For $V \times V$ we consider the metric

$$\begin{aligned} d((x, y), (x', y')) = \max \{d(x, x'), d(y, y')\} \end{aligned}$$

which induces on $V \times V$ the product topology.

Consider the function $d_E:V \times V \rightarrow {\mathbb {R}}$ such that

$$\begin{aligned} d_E(x, y) = d((x, y), E) = \inf \{\, d((x, y), (x', y')) : (x', y') \in E\,\}; \end{aligned}$$

this is a continuous function.

Let $I \subseteq V$ be a nonempty and compact independent set. Since $I \times I$ is compact, the function $d_E$ has a minimum $\delta$ over $I \times I$. Note $\delta > 0$. Indeed, since $I \times I$ is compact, there is $(x, y) \in I \times I$ such that $d((x, y), E) = \delta$. Since *I* is independent, $(x, y) \notin E$. But then from the closedness of *E* there is $\epsilon > 0$ such that $E \cap (B(x, \epsilon ) \times B(y, \epsilon )) = \emptyset$, whence $\delta > 0$.

Next take the set

$$\begin{aligned} S = \bigcup _{x \in I} B(x, \delta ). \end{aligned}$$

This is an open set that contains *I*; it is moreover independent. Indeed, suppose $x'$, $y' \in S$ are adjacent. Take *x*, $y \in I$ such that $x' \in B(x, \delta )$ and $y' \in B(y, \delta )$. Then

$$\begin{aligned} d((x, y), (x', y')) = \max \{d(x, x'), d(y, y')\} < \delta , \end{aligned}$$

a contradiction since $(x', y') \in E$, *x*, $y \in I$, and $d_E(x, y) \ge \delta$. $\square$

Let $G = (V, E)$ be a topological graph and $\omega$ be a Borel measure on *V*. The *independence number* of *G* with respect to the measure $\omega$ is

$$\begin{aligned} \alpha _\omega (G) = \sup \{\, \omega (I) : I \subseteq V \text { is measurable and independent}\,\}; \end{aligned}$$

when speaking of the independence number of a graph, the measure considered will always be clear from the context. The following theorem is a converse of sorts to Theorem 2.2.

### Theorem 2.3

If $G = (V, E)$ is locally independent, then so is $G' = (V, {{\,\mathrm{cl}\,}}E)$. Moreover, if $\omega$ is an inner-regular Borel measure on *V*, then $\alpha _\omega (G') = \alpha _\omega (G)$.

### Proof

Let $I \subseteq V$ be a compact independent set in $G'$. Then *I* is also an independent set in *G* and, since *G* is locally independent, there is an open independent set *S* in *G* that contains *I*. Since *S* is independent, $E \cap (S \times S) = \emptyset$, and hence $E \subseteq (V \times V) {\setminus } (S \times S)$. Now $(V \times V) {\setminus } (S \times S)$ is a closed set and so ${{\,\mathrm{cl}\,}}E \subseteq (V \times V) {\setminus } (S \times S)$, whence *S* is also an independent set in $G'$, finishing the proof that $G'$ is locally independent.

As for the second part of the statement, clearly $\alpha _\omega (G') \le \alpha _\omega (G)$, so we prove the reverse inequality. Since $\omega$ is inner regular, we can restrict ourselves to compact sets, writing

$$\begin{aligned} \alpha _\omega (G) = \sup \{\, \omega (I) : I \subseteq V \text { is compact and independent}\,\}. \end{aligned}$$

So, to prove the reverse inequality, it suffices to show that a compact independent set in *G* is also independent in $G'$. Let *I* be a compact independent set in *G* and let *S* be an open independent set in *G* that contains *I*, which exists since *G* is locally independent. Since *S* is independent, $E \cap (S \times S) = \emptyset$, and hence $E \subseteq (V \times V) {\setminus } (S \times S)$. Now $(V \times V) {\setminus } (S \times S)$ is closed, and so ${{\,\mathrm{cl}\,}}E \subseteq (V \times V) {\setminus } (S \times S)$, whence ${{\,\mathrm{cl}\,}}E \cap (S \times S) = \emptyset$ and ${{\,\mathrm{cl}\,}}E \cap (I \times I) = \emptyset$, that is, *I* is independent in $G'$. $\square$

## A conic programming formulation for the independence number

One of the best polynomial-time-computable upper bounds for the independence number of a finite graph is the theta number, a graph parameter introduced by Lovász [27]. Let $G = (V, E)$ be a finite graph. The theta number and its variants can be defined in terms of the following *conic program*, in which a linear function is maximized over the intersection of a convex cone with an affine subspace:

3 $$\begin{aligned} \begin{array}{r@{\ }l@{\quad }l} \text {maximize}&{}\langle J, A\rangle \\ &{}{{\,\mathrm{tr}\,}}A = 1,\\ &{}A(x, y) = 0&{}\hbox { if}~\ (x, y) \in E,\\ &{}A \in {\mathcal {K}}(V). \end{array} \end{aligned}$$

Here, $A:V \times V \rightarrow {\mathbb {R}}$ is the optimization variable, $J:V \times V \rightarrow {\mathbb {R}}$ is the all-ones matrix, $\langle J, A\rangle = {{\,\mathrm{tr}\,}}JA = \sum _{x,y \in V} A(x, y)$, and ${\mathcal {K}}(V) \subseteq {\mathbb {R}}^{V \times V}$ is a convex cone of symmetric matrices. Both the optimal value of the problem above and the problem itself are denoted by $\vartheta (G, {\mathcal {K}}(V))$.

The *theta number* of *G*, denoted by $\vartheta (G)$, is simply $\vartheta (G, {{\,\mathrm{PSD}\,}}(V))$, where ${{\,\mathrm{PSD}\,}}(V)$ is the cone of positive-semidefinite matrices. In this case our problem becomes a semidefinite program, whose optimal value can be approximated in polynomial time to within any desired precision using the ellipsoid method [19] or interior-point methods [24]. We have moreover $\vartheta (G) \ge \alpha (G)$: if $I \subseteq V$ is a nonempty independent set and $\chi _I:V \rightarrow \{0,1\}$ is its characteristic function, then $A = |I|^{-1} \chi _I \otimes \chi _I^*$, which is the matrix such that

$$\begin{aligned} A(x, y) = |I|^{-1} \chi _I(x) \chi _I(y), \end{aligned}$$

is a feasible solution of $\vartheta (G, {{\,\mathrm{PSD}\,}}(V))$; moreover $\langle J, A\rangle = |I|$, and hence $\vartheta (G) \ge |I|$. Since *I* is any nonempty independent set, $\vartheta (G) \ge \alpha (G)$ follows.

A strengthening of the Lovász theta number is the parameter $\vartheta '(G)$ introduced independently by McEliece et al. [30] and Schrijver [41], obtained by taking ${\mathcal {K}}(V) = {{\,\mathrm{PSD}\,}}(V) \cap {{\,\mathrm{NN}\,}}(V)$, where ${{\,\mathrm{NN}\,}}(V)$ is the cone of matrices with nonnegative entries.

Another choice for ${\mathcal {K}}(V)$ is the cone

$$\begin{aligned} {\mathcal {C}}(V) = {{\,\mathrm{cone}\,}}\{\, f \otimes f^* : f:V \rightarrow {\mathbb {R}}\text { and }f \ge 0\,\} \subseteq {{\,\mathrm{PSD}\,}}(V) \cap {{\,\mathrm{NN}\,}}(V) \end{aligned}$$

of *completely positive matrices*. The proof above that $\vartheta (G) \ge \alpha (G)$ works just as well when ${\mathcal {K}}(V) = {\mathcal {C}}(V)$, and hence

4 $$\begin{aligned} \vartheta (G,{{\,\mathrm{PSD}\,}}(V)) \ge \vartheta (G, {{\,\mathrm{PSD}\,}}(V) \cap {{\,\mathrm{NN}\,}}(V)) \ge \vartheta (G, {\mathcal {C}}(V)) \ge \alpha (G). \end{aligned}$$

De Klerk and Pasechnik [23] observed that a theorem of Motzkin and Straus [33] implies that the last inequality in (4) is actually tight; a streamlined proof of this fact goes as follows. If *A* is a feasible solution of $\vartheta (G, {\mathcal {C}}(V))$, then, after suitable normalization,

5 $$\begin{aligned} A = \alpha _1 f_1 \otimes f_1^* + \cdots + \alpha _n f_n \otimes f_n^*, \end{aligned}$$

where $\alpha _i > 0$, $f_i \ge 0$, and $\Vert f_i\Vert = 1$ for all *i*. Since $\Vert f_i\Vert = 1$, we have ${{\,\mathrm{tr}\,}}f_i \otimes f_i^* = 1$, and then since ${{\,\mathrm{tr}\,}}A = 1$ we must have $\alpha _1 + \cdots + \alpha _n = 1$. It follows that for some *i* we have $\langle J, f_i \otimes f_i^*\rangle \ge \langle J, A\rangle$; assume then that this is the case for $i = 1$.

Next, observe that since $A(x, y) = 0$ for all $(x, y) \in E$ and each $f_i$ is nonnegative, we must have $f_1(x) f_1(y) = 0$ for all $(x, y) \in E$. This implies that *I*, the support of $f_1$, is an independent set. Denoting by $(f, g) = \sum _{x\in V} f(x) g(x)$ the Euclidean inner product in ${\mathbb {R}}^V$, we then have

$$\begin{aligned} \langle J, A\rangle \le \langle J, f_1 \otimes f_1^*\rangle = (f_1, \chi _I)^2 \le \Vert f_1\Vert ^2 \Vert \chi _I\Vert ^2 = |I| \le \alpha (G) \end{aligned}$$

and, since *A* is any feasible solution, we get $\vartheta (G, {\mathcal {C}}(V)) \le \alpha (G)$.

Problem (3) can be naturally extended to infinite topological graphs, as we will see now. Let $G = (V, E)$ be a topological graph where *V* is compact, $\omega$ be a Borel measure on *V*, $J \in L^2(V \times V)$ be the constant 1 kernel, and ${\mathcal {K}}(V) \subseteq L_\mathrm {sym}^2(V \times V)$ be a convex cone of symmetric kernels. When *V* is finite with the discrete topology and $\omega$ is the counting measure, the following optimization problem is exactly (3):

6 $$\begin{aligned} \begin{array}{r@{\ }l@{\quad }l} \text {maximize}&{}\langle J, A\rangle \\ &{}\int _V A(x, x)\, d\omega (x) = 1,\\ &{}A(x, y) = 0\quad \hbox { if}~\ (x, y) \in E,\\ &{}A \text { is continuous and}~A \in {\mathcal {K}}(V). \end{array} \end{aligned}$$

As before, we will denote both the optimal value (that is, the supremum of the objective function) of this problem and the problem itself by $\vartheta (G, {\mathcal {K}}(V))$.

The problem above is a straight-forward extension of (3), except that instead of the trace of the operator *A* we take the integral over the diagonal. Not every Hilbert–Schmidt operator has a trace, so if we were to insist on using the trace instead of the integral, we would have to require that *A* be trace class. Recall that *A* is *trace class* and has trace $\tau$ if for every complete orthonormal system $(f_\alpha )$ of $L^2(V)$ we have

$$\begin{aligned} \tau = \sum _\alpha (A f_\alpha , f_\alpha ). \end{aligned}$$

Mercer’s theorem says that a continuous and positive kernel *A* has a spectral decomposition in terms of continuous eigenfunctions that moreover converges absolutely and uniformly. This implies in particular that *A* is trace class and that its trace is the integral over the diagonal. So, as long as ${\mathcal {K}}(V)$ is a subset of the cone of positive kernels, taking the integral over the diagonal or the trace is the same.

As before, there are at least two cones that can be put in place of ${\mathcal {K}}(V)$. One is the cone ${{\,\mathrm{PSD}\,}}(V)$ of positive kernels. The other is the cone of *completely positive kernels* on *V*, namely

7 $$\begin{aligned} {\mathcal {C}}(V) = {{\,\mathrm{cl}\,}}{{\,\mathrm{cone}\,}}\{\, f \otimes f^* : f \in L^2(V) \text { and}~f \ge 0\,\}, \end{aligned}$$

with the closure taken in the norm topology on $L^2(V \times V)$, and where $f \ge 0$ means that *f* is nonnegative almost everywhere. Note that ${\mathcal {C}}(V) \subseteq {{\,\mathrm{PSD}\,}}(V)$, and hence $\vartheta (G, {{\,\mathrm{PSD}\,}}(V)) \ge \vartheta (G, {\mathcal {C}}(V))$.

### Theorem 3.1

If $G = (V, E)$ is a locally independent graph, if *V* is a compact Hausdorff space, and if $\omega$ is an inner-regular Borel measure on *V* such that $0< \alpha _\omega (G) < \infty$, then $\vartheta (G, {\mathcal {C}}(V)) \ge \alpha _\omega (G)$.

Bachoc et al. [1] proved a similar result for the special case of distance graphs on the sphere; the proof below uses similar ideas.

### Proof

Fix $0< \epsilon < \alpha _\omega (G)$. Since $\omega$ is inner regular and $0< \alpha _\omega (G) < \infty$, there is a compact independent set *I* such that $\omega (I) \ge \alpha _\omega (G) - \epsilon > 0$.

Since *G* is locally independent, there is an open independent set *S* that contains *I*. Now *V* is a compact Hausdorff space and hence normal [16, Proposition 4.25] and *I* and $V {\setminus } S$ are disjoint closed sets, so from Urysohn’s lemma there is a continuous function $f:V \rightarrow [0,1]$ such that $f(x) = 1$ for $x \in I$ and $f(x) = 0$ for $x \in V {\setminus } S$.

Note $\Vert f\Vert > 0$ since $\omega (I) > 0$. Set $A = \Vert f\Vert ^{-2} f \otimes f^*$. Then *A* is a feasible solution of $\vartheta (G, {\mathcal {C}}(V))$. Indeed, *A* is continuous and belongs to ${\mathcal {C}}(V)$, and moreover $\int _V A(x, x)\, d\omega (x) = 1$. Since *S* is independent and *f*’s support is a subset of *S*, $A(x, y) = 0$ if $(x, y) \in E$, and hence *A* is feasible.

Finally, since *S* is independent, $\omega (S) \le \alpha _\omega (G)$. But then $\Vert f\Vert ^2 \le \omega (S)$ and

$$\begin{aligned} \langle J, A \rangle = \frac{\langle J, f \otimes f^*\rangle }{\Vert f\Vert ^2} \ge \frac{\omega (I)^2}{\omega (S)} \ge \frac{(\alpha _\omega (G) - \epsilon )^2}{\alpha _\omega (G)}. \end{aligned}$$

Since $\epsilon$ is any positive number, the theorem follows. $\square$

Theorem 5.1 in Sect. 5 states that, under some extra assumptions on *G* and $\omega$, one has $\vartheta (G, {\mathcal {C}}(V)) = \alpha _\omega (G)$, as in the finite case. The proof of this theorem is fundamentally the same as in the finite case; here is an intuitive description.

There are two key steps in the proof for finite graphs as given above. First, the matrix *A* is a convex combination of rank-one nonnegative matrices, as in (5). Second, this together with the constraints of our problem implies that the support of each $f_i$ in (5) is an independent set. Then the support of one of the $f_i$s will give us a large independent set.

In the proof that $\vartheta (G, {\mathcal {C}}(V)) = \alpha _\omega (G)$ for an infinite topological graph we will have to repeat the two steps above. Now *A* will be a kernel, so it will not be in general a convex combination of finitely many rank-one kernels as in (5); Choquet’s theorem [43, Theorem 10.7] will allow us to express *A* as a sort of convex combination of infinitely many rank-one kernels. Next, it will not be the case that the support of any function appearing in the decomposition of *A* will be independent, but depending on some properties of *G* and $\omega$ we will be able to fix this by removing from the support the measure-zero set consisting of all points that are not density points.

To be able to apply Choquet’s theorem, we first need to better understand the cone ${\mathcal {C}}(V)$; this we do next.

## The completely positive and the copositive cones on compact spaces

Throughout this section, *V* will be a compact Hausdorff space and $\omega$ will be a finite Borel measure on *V* such that every open set has positive measure and $\omega (V) = 1$; the normalization of $\omega$ is made for convenience only.

For $f \in L^2(V)$ and $g \in L^\infty (V)$, write $f \odot g$ for the function $x \mapsto f(x) g(x)$; note that $f \odot g \in L^2(V)$. For $A \in L^2(V \times V)$ and $B \in L^\infty (V \times V)$, define $A \odot B$ analogously. For $U \subseteq V$ and $A \in L^2(V \times V)$, denote by *A*[*U*] the restriction of *A* to $U \times U$.

There are two useful topologies to consider on the $L^2$ spaces we deal with: the norm topology and the weak topology. We begin with a short discussion about them, based on Chapter 5 of Simon [43]. Statements will be given in terms of $L^2(V)$, but they also hold for $L^2(V \times V)$ and $L_\mathrm {sym}^2(V \times V)$.

The norm topology on $L^2(V)$ coincides with the *Mackey topology*, the strongest topology for which only the linear functionals $f \mapsto (f, g)$ for $g \in L^2(V)$ are continuous.

The *weak topology* on $L^2(V)$ is the weakest topology for which all linear functionals $f \mapsto (f, g)$ for $g \in L^2(V)$ are continuous. A net^1^ $(f_\alpha )$ converges in the weak topology if and only if $((f_\alpha , g))$ converges for all $g \in L^2(V)$.

The weak and norm topologies are *dual topologies*, that is, the topological dual of $L^2(V)$ is the same for both topologies, and hence it is isomorphic to $L^2(V)$. Theorem 5.2 (iv) (ibid.) says that if $X \subseteq L^2(V)$ is a convex set, then ${{\,\mathrm{cl}\,}}X$ is the same whether it is taken in the weak or norm topology. Since the set

$$\begin{aligned} {{\,\mathrm{cone}\,}}\{\, f \otimes f^* : \hbox {} f \in L^2(V) \hbox {and}~ f \ge 0\,\} \end{aligned}$$

is convex, it follows that if we take the closure in (7) in the weak topology we also obtain ${\mathcal {C}}(V)$.

The dual cone of ${\mathcal {C}}(V)$ is

$$\begin{aligned} {\mathcal {C}}^*(V) = \{\, Z \in L_\mathrm {sym}^2(V \times V) : \langle Z, f \otimes f^*\rangle \ge 0 \text { for all}~f \in L^2(V)\text { with}~f \ge 0\,\}; \end{aligned}$$

it is the cone of *copositive kernels* on *V*. This is a convex cone and, since it is closed in the weak topology on $L_\mathrm {sym}^2(V \times V)$, it is also closed in the norm topology. Moreover, the dual of ${\mathcal {C}}^*(V)$, namely

$$\begin{aligned} ({\mathcal {C}}^*(V))^* = \{\, A \in L_\mathrm {sym}^2(V \times V) : \hbox {} \langle Z, A\rangle \ge 0 \hbox { for all}~ Z \in {\mathcal {C}}^*(V)\,\} \end{aligned}$$

is exactly ${\mathcal {C}}(V)$ by the Bipolar Theorem [43, Theorem 5.5]; see also Problem 1, §IV.5.3 in Barvinok [3].

### Theorem 4.1

Let $A \in {\mathcal {C}}(V)$ and $Z \in {\mathcal {C}}^*(V)$. Then:

(i) If $U \subseteq V$ is measurable and has positive measure, then $A[U] \in {\mathcal {C}}(U)$ and $Z[U] \in {\mathcal {C}}^*(U)$, where *U* inherits its topology and measure from *V*.
(ii) If $g \in L^\infty (V)$ is nonnegative, then $A \odot (g \otimes g^*) \in {\mathcal {C}}(V)$ and $Z \odot (g \otimes g^*) \in {\mathcal {C}}^*(V)$.

### Proof

The first statement is immediate, so let us prove the second. If $f \in L^2(V)$ is nonnegative, then $f \odot g \ge 0$, and so $(f \otimes f^*) \odot (g \otimes g^*) = (f \odot g) \otimes (f \odot g)^* \in {\mathcal {C}}(V)$. This implies that if $A \in {\mathcal {C}}(V)$, then $A \odot (g \otimes g^*) \in {\mathcal {C}}(V)$.

Now take $Z \in {\mathcal {C}}^*(V)$. If $f \in L^2(V)$ is nonnegative, then

$$\begin{aligned} \langle Z \odot (g \otimes g^*), f \otimes f^*\rangle = \langle Z, (f \odot g) \otimes (f \odot g)^*\rangle \ge 0, \end{aligned}$$

and hence $$Z \odot (g \otimes g^*) \in {\mathcal {C}}^*(V)$$. $$\square $$

### Partitions and averaging^2^

An $\omega$-*partition* of *V* is a partition of *V* into finitely many measurable sets each of positive measure. Given a function $f \in L^2(V)$ and an $\omega$-partition ${\mathcal {P}}$ of *V*, the *averaging* of *f* on ${\mathcal {P}}$ is the function $f *{\mathcal {P}}:V \rightarrow {\mathbb {R}}$ such that

$$\begin{aligned} (f *{\mathcal {P}})(x) = \omega (X)^{-1} \int _X f(x')\, d\omega (x') \end{aligned}$$

for all $X \in {\mathcal {P}}$ and $x \in X$. It is immediate that $f *{\mathcal {P}}\in L^2(V)$. We also see $f *{\mathcal {P}}$ as a function with domain ${\mathcal {P}}$, writing $(f*{\mathcal {P}})(X)$ for the common value of $f*{\mathcal {P}}$ in $X \in {\mathcal {P}}$.

Given $A \in L^2(V \times V)$, the *averaging* of *A* on ${\mathcal {P}}$ is the function $A*{\mathcal {P}}:V \times V \rightarrow {\mathbb {R}}$ such that

$$\begin{aligned} (A*{\mathcal {P}})(x, y) = \omega (X)^{-1} \omega (Y)^{-1} \int _X \int _Y A(x', y')\, d\omega (y') d\omega (x') \end{aligned}$$

for all *X*, $Y \in {\mathcal {P}}$ and $x \in X$, $y \in Y$. Again, $A*{\mathcal {P}}\in L^2(V \times V)$; moreover, if *A* is symmetric, then so is $A*{\mathcal {P}}$. The kernel $A*{\mathcal {P}}$ can also be seen as a function with domain ${\mathcal {P}}\times {\mathcal {P}}$ (that is, as a matrix), so $(A*{\mathcal {P}})(X, Y)$ is the common value of $A*{\mathcal {P}}$ in $X \times Y$ for *X*, $Y \in {\mathcal {P}}$. Seeing $A*{\mathcal {P}}$ as a matrix allows us to show that, as a kernel, $A*{\mathcal {P}}$ has finite rank. Note also that $(f \otimes f^*)*{\mathcal {P}}= (f*{\mathcal {P}}) \otimes (f*{\mathcal {P}})^*$.

The averaging operation preserves step functions and step kernels on the partition ${\mathcal {P}}$. In particular, it is idempotent: if $f \in L^2(V)$, then $(f*{\mathcal {P}})*{\mathcal {P}}= f*{\mathcal {P}}$, and similarly for kernels. Moreover, if *A*, $B \in L^2(V \times V)$, then

$$\begin{aligned} \langle A*{\mathcal {P}}, B\rangle = \langle A*{\mathcal {P}}, B*{\mathcal {P}}\rangle = \langle A, B*{\mathcal {P}}\rangle . \end{aligned}$$

For a proof, simply expand all the inner products. On the one hand,

$$\begin{aligned} \begin{aligned} \langle A*{\mathcal {P}}, B*{\mathcal {P}}\rangle&=\sum _{X,Y \in {\mathcal {P}}} \int _X \int _Y (A*{\mathcal {P}})(x, y) (B*{\mathcal {P}})(x, y)\, d\omega (y) d\omega (x)\\&=\sum _{X, Y \in {\mathcal {P}}} (A*{\mathcal {P}})(X, Y) (B*{\mathcal {P}})(X, Y) \omega (X) \omega (Y). \end{aligned} \end{aligned}$$

On the other hand,

$$\begin{aligned} \begin{aligned} \langle A*{\mathcal {P}}, B\rangle&= \sum _{X, Y \in {\mathcal {P}}} \int _X \int _Y (A*{\mathcal {P}})(x, y) B(x, y)\, d\omega (y) d\omega (x)\\&= \sum _{X, Y \in {\mathcal {P}}} (A*{\mathcal {P}})(X, Y) \int _X \int _Y B(x, y)\, d\omega (y) d\omega (x)\\&= \sum _{X, Y \in {\mathcal {P}}} (A*{\mathcal {P}})(X, Y) (B*{\mathcal {P}})(X, Y) \omega (X) \omega (Y)\\&=\langle A*{\mathcal {P}}, B*{\mathcal {P}}\rangle . \end{aligned} \end{aligned}$$

One concludes similarly that $\langle A, B*{\mathcal {P}}\rangle = \langle A*{\mathcal {P}}, B*{\mathcal {P}}\rangle$.

#### Theorem 4.2

Let ${\mathcal {P}}$ be an $\omega$-partition. If $A \in {\mathcal {C}}(V)$, then $A*{\mathcal {P}}\in {\mathcal {C}}(V)$ and $A*{\mathcal {P}}\in {\mathcal {C}}({\mathcal {P}})$, where on ${\mathcal {P}}$ we consider the discrete topology and the counting measure. Similarly, if $Z \in {\mathcal {C}}^*(V)$, then $Z*{\mathcal {P}}\in {\mathcal {C}}^*(V)$ and $Z*{\mathcal {P}}\in {\mathcal {C}}^*({\mathcal {P}})$.

#### Proof

Let us prove the second statement first. Take $Z \in {\mathcal {C}}^*(V)$ and $f \in L^2(V)$ with $f \ge 0$. Then $f*{\mathcal {P}}\ge 0$ and

$$\begin{aligned} \langle Z*{\mathcal {P}}, f\otimes f^*\rangle = \langle Z, (f \otimes f^*) *{\mathcal {P}}\rangle = \langle Z, (f*{\mathcal {P}}) \otimes (f*{\mathcal {P}})^*\rangle \ge 0, \end{aligned}$$

whence $$Z*{\mathcal {P}}\in {\mathcal {C}}^*(V)$$.

To see that $Z*{\mathcal {P}}\in {\mathcal {C}}^*({\mathcal {P}})$, take a function $\phi :{\mathcal {P}}\rightarrow {\mathbb {R}}$ with $\phi \ge 0$. Let $f \in L^2(V)$ be the function such that $f(x) = \phi (X) \omega (X)^{-1}$ for all $X \in {\mathcal {P}}$ and $x \in X$; notice $f \ge 0$. Then

$$\begin{aligned} \begin{aligned}&\sum _{X,Y \in {\mathcal {P}}} (Z*{\mathcal {P}})(X, Y) \phi (X) \phi (Y)\\&\qquad =\sum _{X,Y \in {\mathcal {P}}} \int _X \int _Y (Z*{\mathcal {P}})(x, y) \phi (X) \phi (Y) \omega (X)^{-1} \omega (Y)^{-1}\, d\omega (y) d\omega (x)\\&\qquad =\langle Z*{\mathcal {P}}, f \otimes f^*\rangle \ge 0, \end{aligned} \end{aligned}$$

and $$Z*{\mathcal {P}}\in {\mathcal {C}}^*({\mathcal {P}})$$.

Now take $A \in {\mathcal {C}}(V)$. If $Z \in {\mathcal {C}}^*(V)$, then since $Z*{\mathcal {P}}\in {\mathcal {C}}^*(V)$ we have

$$\begin{aligned} \langle A*{\mathcal {P}}, Z\rangle = \langle A, Z*{\mathcal {P}}\rangle \ge 0. \end{aligned}$$

So, since $({\mathcal {C}}^*(V))^* = {\mathcal {C}}(V)$, we have $A*{\mathcal {P}}\in {\mathcal {C}}(V)$.

Seeing that $A*{\mathcal {P}}\in {\mathcal {C}}({\mathcal {P}})$ is only slightly more complicated. Given $Z \in {\mathcal {C}}^*({\mathcal {P}})$, consider the kernel $Z' \in L^2(V \times V)$ such that $Z'(x, y) = Z(X, Y) \omega (X)^{-1} \omega (Y)^{-1}$ for all *X*, $Y \in {\mathcal {P}}$ and $x \in X$, $y \in Y$. Then $Z' \in {\mathcal {C}}^*(V)$. Indeed, let $f \in L^2(V)$ be nonnegative. Note $Z'*{\mathcal {P}}= Z'$ and expand $\langle Z', f \otimes f^*\rangle$ to get

$$\begin{aligned} \begin{aligned}&\langle Z', f \otimes f^*\rangle = \langle Z'*{\mathcal {P}}, f\otimes f^*\rangle = \langle Z', (f*{\mathcal {P}}) \otimes (f*{\mathcal {P}})^*\rangle \\&\qquad =\sum _{X, Y \in {\mathcal {P}}} \int _X \int _Y Z(X, Y) \omega (X)^{-1} \omega (Y)^{-1} (f*{\mathcal {P}})(X) (f*{\mathcal {P}})(Y)\, d\omega (y)d\omega (x)\\&\qquad =\sum _{X,Y \in {\mathcal {P}}} Z(X, Y) (f*{\mathcal {P}})(X) (f*{\mathcal {P}})(Y) \ge 0, \end{aligned} \end{aligned}$$

since $f*{\mathcal {P}}\ge 0$. So $Z' \in {\mathcal {C}}^*(V)$. Now, since $A*{\mathcal {P}}\in {\mathcal {C}}(V)$ and $Z'\in {\mathcal {C}}^*(V)$,

$$\begin{aligned} \sum _{X, Y \in {\mathcal {P}}} (A*{\mathcal {P}})(X, Y) Z(X, Y) = \langle A*{\mathcal {P}}, Z'\rangle \ge 0, \end{aligned}$$

and $$A*{\mathcal {P}}\in {\mathcal {C}}({\mathcal {P}})$$. $$\square $$

#### Corollary 4.3

If ${\mathcal {P}}$ is an $\omega$-partition and if $A \in {\mathcal {C}}(V)$, then there are nonnegative and nonzero functions $f_1$, ..., $f_n \in L^2(V)$, each one constant in each $X \in {\mathcal {P}}$, such that

$$\begin{aligned} A*{\mathcal {P}}= f_1 \otimes f_1^* + \cdots + f_n \otimes f_n^*. \end{aligned}$$

#### Proof

From Theorem 4.2 we know that $A*{\mathcal {P}}\in {\mathcal {C}}({\mathcal {P}})$. So there are nonnegative and nonzero functions $\phi _1$, ..., $\phi _n$ with domain ${\mathcal {P}}$ such that

$$\begin{aligned} A*{\mathcal {P}}= \phi _1 \otimes \phi _1^* + \cdots + \phi _n \otimes \phi _n^*, \end{aligned}$$

where $A*{\mathcal {P}}$ is seen as a function on ${\mathcal {P}}\times {\mathcal {P}}$. The result now follows by taking $f_i(x) = \phi _i(X)$ for $X \in {\mathcal {P}}$ and $x \in X$. $\square$

### Approximation of continuous kernels

The main use of averaging is in approximating continuous kernels by finite-rank ones. We say that a continuous kernel $A:V \times V \rightarrow {\mathbb {R}}$ *varies* (strictly) less than $\epsilon$ over an $\omega$-partition ${\mathcal {P}}$ if the variation of *A* in each $X \times Y$ for *X*, $Y \in {\mathcal {P}}$ is less than $\epsilon$. We say that a partition ${\mathcal {P}}$ of *V* *separates* $U \subseteq V$ if $|U \cap X| \le 1$ for all $X \in {\mathcal {P}}$. The main tool we need is the following result.

#### Theorem 4.4

If $A:V \times V \rightarrow {\mathbb {R}}$ is continuous and if $U \subseteq V$ is finite, then for every $\epsilon > 0$ there is an $\omega$-partition ${\mathcal {P}}$ that separates *U* and over which *A* varies less than $\epsilon$.

#### Proof

Since *V* is a Hausdorff space and *U* is finite, every $x \in V$ has a neighborhood $N_x$ such that every $y \in U {\setminus } \{x\}$ is in the exterior of $N_x$. Since *A* is continuous, for every $(x, y) \in V \times V$ we can choose neighborhoods $N_{x,y}^x$ of *x* and $N_{x,y}^y$ of *y* such that the variation of *A* in $N_{x,y}^x \times N_{x,y}^y$ is less than $\epsilon / 2$. The same is then true of the neighborhoods $N_{x,y}^x \cap N_x$ and $N_{x,y}^y \cap N_y$ of *x* and *y*.

The sets $(N_{x,y}^x \cap N_x) \times (N_{x,y}^y \cap N_y)$ form an open cover of $V \times V$, and since $V \times V$ is compact there is a finite subcover ${\mathcal {B}}$ consisting of such sets. Set

$$\begin{aligned} {\mathcal {C}}= \{\, S \subseteq V : \text {there is}~T \text { such that}~(S, T) \text {or}~(T, S) \in {\mathcal {B}}\,\}. \end{aligned}$$

Note ${\mathcal {C}}$ is an open cover of *V*. Moreover, by construction, $|U \cap S| \le 1$ for all $S \in {\mathcal {C}}$ and, if $x \in U$ is such that $x \notin S$ for some $S \in {\mathcal {C}}$, then *x* is in the exterior of *S*. Let us turn this open cover ${\mathcal {C}}$ into the desired $\omega$-partition ${\mathcal {P}}$.

For ${\mathcal {S}}\subseteq {\mathcal {C}}$, consider the set

$$\begin{aligned} E_{\mathcal {S}}= \bigcap _{S \in {\mathcal {S}}} S {\setminus } \bigcup _{S \in {\mathcal {C}}{\setminus } {\mathcal {S}}} S = \bigcap _{S \in {\mathcal {S}}} S \cap \bigcap _{S \in {\mathcal {C}}{\setminus }{\mathcal {S}}} V {\setminus } S. \end{aligned}$$

Write ${\mathcal {R}}= \{\, E_{\mathcal {S}}: {\mathcal {S}}\subseteq {\mathcal {C}}\text {and}~E_{\mathcal {S}}\ne \emptyset \,\}$. Then ${\mathcal {R}}$ is a partition of *V* that, by construction, separates *U*. Moreover, if *X*, $Y \in {\mathcal {R}}$, then the variation of *A* in $X \times Y$ is less than $\epsilon / 2$. Indeed, note that if ${\mathcal {S}}\subseteq {\mathcal {C}}$ and $S \in {\mathcal {C}}$ are such that $E_{\mathcal {S}}\cap S \ne \emptyset$, then $E_{\mathcal {S}}\subseteq S$. Since ${\mathcal {B}}$ is a cover of $V \times V$, given *X*, $Y \in {\mathcal {R}}$ there must be $S \times T \in {\mathcal {B}}$ such that $(X \times Y) \cap (S \times T) \ne \emptyset$, implying that $X \cap S \ne \emptyset$ and $Y \cap T \ne \emptyset$, whence $X \subseteq S$ and $Y \subseteq T$. But then $X \times Y \subseteq S \times T$, and we know that the variation of *A* in $S \times T$ is less than $\epsilon / 2$.

Now ${\mathcal {R}}$ may not be an $\omega$-partition: though the sets in ${\mathcal {R}}$ are measurable, some may have measure 0. This does not happen, however, for sets in ${\mathcal {R}}$ that contain some point in *U*. Indeed, if for ${\mathcal {S}}\subseteq {\mathcal {C}}$ and $x \in U$ we have $x \in E_{\mathcal {S}}$, then $x \in \bigcap _{S \in {\mathcal {S}}} S$, which is an open set. Moreover, $x \notin S$ for all $S \in {\mathcal {C}}{\setminus } {\mathcal {S}}$, and hence *x* is in the exterior of each $S \in {\mathcal {C}}{\setminus } {\mathcal {S}}$. But then *x* is in the interior of $E_{\mathcal {S}}$ and so $E_{\mathcal {S}}$ has nonempty interior and hence positive measure.

Let us fix ${\mathcal {R}}$ by getting rid of sets with measure 0. Let *W* be the union of all sets in ${\mathcal {R}}$ with measure 0. Note ${{\,\mathrm{cl}\,}}(V{\setminus } W) = V$. For if not, then there would be $x \in W$ and a neighborhood *N* of *x* such that $N \cap {{\,\mathrm{cl}\,}}(V{\setminus } W) = \emptyset$. But then $N \subseteq V {\setminus } {{\,\mathrm{cl}\,}}(V{\setminus } W) \subseteq W$, and hence $\omega (W) > 0$, a contradiction.

Let $X_1$, ..., $X_n$ be the sets of positive measure in ${\mathcal {R}}$. Set

$$\begin{aligned} X_i' = X_i \cup (W \cap {{\,\mathrm{cl}\,}}X_i) {\setminus } (X_1' \cup \cdots \cup X_{i-1}'). \end{aligned}$$

Since $V = {{\,\mathrm{cl}\,}}(V{\setminus } W) = {{\,\mathrm{cl}\,}}X_1 \cup \cdots \cup {{\,\mathrm{cl}\,}}X_n$, ${\mathcal {P}}= \{X_1', \ldots , X_n'\}$ is an $\omega$-partition of *V*; moreover, since $U \cap W = \emptyset$, ${\mathcal {P}}$ separates *U*. Now $X_i' \subseteq {{\,\mathrm{cl}\,}}X_i$, and so the variation of *A* in $X \times Y$ for *X*, $Y \in {\mathcal {P}}$ is at most $\epsilon / 2$, and hence less than $\epsilon$. $\square$

The existence of $\omega$-partitions over which *A* has small variation allows us to approximate a continuous kernel by its averages.

#### Theorem 4.5

If a continuous kernel $A:V \times V \rightarrow {\mathbb {R}}$ varies less than $\epsilon$ over an $\omega$-partition ${\mathcal {P}}$, then $|A(x, y) - (A*{\mathcal {P}})(x, y)| < \epsilon$ for all *x*, $y \in V$.

#### Proof

Take *x*, $y \in V$ and say $x \in X$, $y \in Y$ for some *X*, $Y \in {\mathcal {P}}$. Then

$$\begin{aligned} \begin{aligned} (A*{\mathcal {P}})(x, y)&= \omega (X)^{-1} \omega (Y)^{-1} \int _X \int _Y A(x', y')\, d\omega (y') d\omega (x')\\&< \omega (X)^{-1} \omega (Y)^{-1} \int _X \int _Y A(x, y) + \epsilon \, d\omega (y') d\omega (x')\\&= A(x, y) + \epsilon . \end{aligned} \end{aligned}$$

Similarly, $(A*{\mathcal {P}})(x, y) > A(x, y) - \epsilon$, and the theorem follows. $\square$

#### Corollary 4.6

If a continuous kernel $A:V \times V \rightarrow {\mathbb {R}}$ varies less than $\epsilon$ over an $\omega$-partition ${\mathcal {P}}$, then $\Vert A - A*{\mathcal {P}}\Vert < \epsilon$. If moreover *A* is positive, then $|{{\,\mathrm{tr}\,}}A - {{\,\mathrm{tr}\,}}A*{\mathcal {P}}| < \epsilon$.

#### Proof

Using Theorem 4.5 we get

$$\begin{aligned} \Vert A - A*{\mathcal {P}}\Vert ^2 = \int _V \int _V (A(x, y) - (A*{\mathcal {P}})(x, y))^2\, d\omega (y) d\omega (x) < \epsilon ^2, \end{aligned}$$

as desired.

Since *A* is positive and continuous, Mercer’s theorem implies that the trace of *A* is the integral over the diagonal. Since $A*{\mathcal {P}}$ is a finite-rank step kernel, its trace is also the integral over the diagonal. Then, using Theorem 4.5,

$$\begin{aligned} \begin{aligned} |{{\,\mathrm{tr}\,}}A - {{\,\mathrm{tr}\,}}A*{\mathcal {P}}|&= \biggl | \int _V A(x, x) - (A*{\mathcal {P}})(x, x)\, d\omega (x)\biggr |\\&\le \int _V |A(x, x) - (A*{\mathcal {P}})(x, x)|\, d\omega (x)\\&<\epsilon , \end{aligned} \end{aligned}$$

as we wanted. $\square$

A continuous kernel $A:V \times V \rightarrow {\mathbb {R}}$ is positive if and only if the matrix *A*[*U*] is positive semidefinite for all finite $U \subseteq V$ (cf. Bochner [4]). An analogous result holds for ${\mathcal {C}}(V)$ and its dual; see also Lemma 2.1 of Dobre et al. [12].

#### Theorem 4.7

A continuous kernel $A:V \times V \rightarrow {\mathbb {R}}$ belongs to ${\mathcal {C}}(V)$ if and only if *A*[*U*] belongs to ${\mathcal {C}}(U)$ for all finite $U \subseteq V$, where we consider for *U* the discrete topology and the counting measure. Likewise, a continuous $Z:V \times V \rightarrow {\mathbb {R}}$ belongs to ${\mathcal {C}}^*(V)$ if and only if *Z*[*U*] belongs to ${\mathcal {C}}^*(U)$ for all finite $U \subseteq V$.

#### Proof

Take $A \in {\mathcal {C}}(V)$ and let $U \subseteq V$ be finite. For $n \ge 1$, let ${\mathcal {P}}_n$ be an $\omega$-partition that separates *U* and over which *A* varies less than 1/*n*, as given by Theorem 4.4. Since $A*{\mathcal {P}}_n \in {\mathcal {C}}({\mathcal {P}}_n)$ and ${\mathcal {P}}_n$ separates *U*, Theorem 4.2 implies that $(A*{\mathcal {P}}_n)[U] \in {\mathcal {C}}(U)$ for all $n \ge 1$; Theorem 4.5 implies that *A*[*U*] is the limit, in the norm topology, of $((A*{\mathcal {P}}_n)[U])$, so $A[U] \in {\mathcal {C}}(U)$. One proves similarly that if $Z \in {\mathcal {C}}^*(V)$, then $Z[U] \in {\mathcal {C}}^*(U)$ for all finite $U \subseteq V$.

Now let $A:V \times V \rightarrow {\mathbb {R}}$ be a continuous kernel such that $A \notin {\mathcal {C}}(V)$. Let us show that there is a finite set $U \subseteq V$ such that $A[U] \notin {\mathcal {C}}(U)$. If *A* is not symmetric, we are done. So assume *A* is symmetric and let $Z \in {\mathcal {C}}^*(V)$ be such that $\langle A, Z \rangle = \delta < 0$.

Corollary 4.6 together with the Cauchy–Schwarz inequality implies that, if *A* varies less than $\epsilon$ over an $\omega$-partition ${\mathcal {P}}$, then $|\langle A, Z \rangle - \langle A*{\mathcal {P}}, Z\rangle | < \epsilon \Vert Z\Vert$. So, for all small enough $\epsilon$, if *A* varies less than $\epsilon$ over the $\omega$-partition ${\mathcal {P}}$, then

8 $$\begin{aligned} \delta / 2 > \langle A*{\mathcal {P}}, Z\rangle = \langle A*{\mathcal {P}}, Z*{\mathcal {P}}\rangle = \sum _{X, Y \in {\mathcal {P}}} (A*{\mathcal {P}})(X, Y) (Z*{\mathcal {P}})(X, Y) \omega (X) \omega (Y).\nonumber \\ \end{aligned}$$

Let $g \in L^\infty (V)$ be the function such that $g(x) = \omega (X)$ for $X \in {\mathcal {P}}$ and $x \in X$. Theorems 4.1 and 4.2 say that $Z' = (Z*{\mathcal {P}}) \odot (g \otimes g^*) \in {\mathcal {C}}^*(V)$. For *x*, $y \in V$, write $s(x, y) = {{\,\mathrm{sgn}\,}}Z'(x, y)$. Let $U \subseteq V$ be a set of representatives of the parts of ${\mathcal {P}}$. Develop (8) using Theorem 4.5 to obtain

9 $$\begin{aligned} \begin{aligned} \delta / 2&> \sum _{x, y \in U} (A*{\mathcal {P}})(x, y) Z'(x, y)\\&\ge \sum _{x, y \in U} (A(x, y) - s(x, y)\epsilon ) Z'(x, y)\\&=\sum _{x, y \in U} A(x, y) Z'(x, y) - \epsilon \sum _{x, y \in U} s(x, y) Z'(x, y). \end{aligned} \end{aligned}$$

Now notice that, if ${\mathcal {P}}$ is an $\omega$-partition, then $\Vert Z*{\mathcal {P}}\Vert _1 \le \Vert Z\Vert _1$. So

$$\begin{aligned} \sum _{x,y \in U} s(x, y) Z'(x, y) = \Vert Z*{\mathcal {P}}\Vert _1 \le \Vert Z\Vert _1. \end{aligned}$$

Together with (9) this gives

$$\begin{aligned} \sum _{x, y \in U} A(x, y) Z'(x, y) < \delta / 2 + \epsilon \Vert Z\Vert _1. \end{aligned}$$

Since *U* is a set of representatives of the parts of ${\mathcal {P}}$, Theorem 4.2 says $Z'[U] \in {\mathcal {C}}^*(U)$. Since $\Vert Z\Vert _1 < \infty$ (as $\omega$ is finite, $L^2(V \times V) \subseteq L^1(V \times V)$), by taking $\epsilon$ sufficiently small we see that $A[U] \notin {\mathcal {C}}(U)$, as we wanted.

The analogous result for ${\mathcal {C}}^*(V)$ can be similarly proved. $\square$

Using Theorem 4.7, we can rewrite problem $\vartheta (G, {\mathcal {C}}(V))$ (see (6)) by replacing the constraint “$A \in {\mathcal {C}}(V)$” by infinitely many constraints on finite subkernels of *A*.

### The tip of the cone of completely positive kernels

A *base* of a cone *K* is a set $B \subseteq K$ that does not contain the origin and is such that for every nonzero $x \in K$ there is a unique $\alpha > 0$ for which $\alpha ^{-1} x \in B$. Cones with compact and convex bases have many pleasant properties that are particularly useful to the theory of conic programming [3, Chapter IV].

It is not in general clear whether ${\mathcal {C}}(V)$ has a compact and convex base, however the following subset of ${\mathcal {C}}(V)$ — its *tip* — will be just as useful in the coming developments:

$$\begin{aligned} {\mathcal {T}}(V) = {{\,\mathrm{cch}\,}}\{\, f \otimes f^* : f \in L^2(V), f \ge 0, \text { and}~\Vert f\Vert \le 1\,\}, \end{aligned}$$

where ${{\,\mathrm{cch}\,}}X$ is the closure of the convex hull of *X*. Notice the closure is the same whether taken in the norm or the weak topology.

If $\Vert f\Vert \le 1$, then $\Vert f\otimes f^*\Vert = \Vert f\Vert ^2 \le 1$, so ${\mathcal {T}}(V)$ is a closed subset of the closed unit ball in $L^2(V \times V)$, and hence by Alaoglu’s theorem [16, Theorem 5.18] it is weakly compact. If $L^2(V \times V)$ is separable, then the weak topology on the closed unit ball of $L^2(V \times V)$, and hence the weak topology on ${\mathcal {T}}(V)$, is metrizable [16, p. 171, Exercise 50].

The tip displays a key property of a base, at least for continuous kernels.

#### Theorem 4.8

If $A \in {\mathcal {C}}(V)$ is nonzero and continuous, then $({{\,\mathrm{tr}\,}}A)^{-1} A \in {\mathcal {T}}(V)$.

#### Proof

For $n \ge 1$, let ${\mathcal {P}}_n$ be an $\omega$-partition over which *A* varies less than 1/*n*. For each $n \ge 1$, use Corollary 4.3 to write

$$\begin{aligned} A*{\mathcal {P}}_n = \sum _{m=1}^{r_n} \alpha _{mn} f_{mn} \otimes f_{mn}^*, \end{aligned}$$

where $$\alpha _{mn} \ge 0$$, $$f_{mn} \ge 0$$, and $$\Vert f_{mn}\Vert = 1$$.

The kernel *A* is in ${\mathcal {C}}(V)$ and hence positive, so using Corollary 4.6 we have

$$\begin{aligned} \lim _{n\rightarrow \infty } ({{\,\mathrm{tr}\,}}A*{\mathcal {P}}_n)^{-1} A*{\mathcal {P}}_n = ({{\,\mathrm{tr}\,}}A)^{-1} A \end{aligned}$$

in the norm topology. Now ${{\,\mathrm{tr}\,}}A*{\mathcal {P}}_n = \sum _{m=1}^{r_n} \alpha _{mn} > 0$ for all large enough *n*, and then $({{\,\mathrm{tr}\,}}A*{\mathcal {P}}_n)^{-1} A*{\mathcal {P}}_n \in {\mathcal {T}}(V)$ for all large enough *n*, proving the theorem. $\square$

Finally, we also know how the extreme points of ${\mathcal {T}}(V)$ look like.

#### Theorem 4.9

An extreme point of ${\mathcal {T}}(V)$ is either 0 or of the form $f \otimes f^*$ for $f \in L^2(V)$ with $f \ge 0$ and $\Vert f\Vert = 1$.

#### Proof

We show first that the set ${\mathcal {B}}= \{\, f \otimes f^* : \hbox {} f \in L^2(V) \hbox {,} f \ge 0$, and $\Vert f\Vert \le 1\,\}$ is weakly closed. Then, since ${\mathcal {T}}(V)$ is weakly compact and convex and since the weak topology is locally convex, it will follow from Milman’s theorem [43, Theorem 9.4] that all extreme points of ${\mathcal {T}}(V)$ are contained in ${\mathcal {B}}$.

Let $(f_\alpha \otimes f_\alpha ^*)$ be a weakly converging net with $f_\alpha \in L^2(V)$, $f_\alpha \ge 0$, and $\Vert f_\alpha \Vert \le 1$ for all $\alpha$. The net $(f_\alpha )$ lies in the closed unit ball, which is weakly compact, and hence it has a weakly converging subnet. So we may assume that the net $(f_\alpha )$ is itself weakly converging; let *f* be its limit.

Immediately we have $f \ge 0$ and $\Vert f\Vert \le 1$. Claim: $f \otimes f^*$ is the limit of $(f_\alpha \otimes f_\alpha ^*)$. Proof: We have to show that, if $G \in L^2(V \times V)$, then

10 $$\begin{aligned} \langle f_\alpha \otimes f_\alpha ^*, G \rangle \rightarrow \langle f \otimes f^*, G \rangle . \end{aligned}$$

Let *S* be a complete orthonormal system of $L^2(V)$; then $\{\, g \otimes h^* : \hbox {} g \hbox {,} h \in S\,\}$ is a complete orthonormal system of $L^2(V \times V)$. Given $G \in L^2(V \times V)$, write

$$\begin{aligned} G = \sum _{i=1}^\infty \lambda _i g_i \otimes h_i^*, \end{aligned}$$

where $g_i$, $h_i \in S$ and $\sum _{i=1}^\infty \lambda _i^2 = \Vert G\Vert ^2$. For every $\epsilon > 0$, let $N_\epsilon$ be such that the finite-rank kernel

$$\begin{aligned} G_\epsilon = \sum _{i=1}^{N_\epsilon } \lambda _i g_i \otimes h_i^* \end{aligned}$$

satisfies $\Vert G - G_\epsilon \Vert < \epsilon$. Apply the Cauchy–Schwarz inequality to get

11 $$\begin{aligned} |\langle g \otimes h^*, G\rangle - \langle g \otimes h^*, G_\epsilon \rangle | < \epsilon \end{aligned}$$

for every *g*, $h \in L^2(V)$ with $\Vert g\Vert = \Vert h\Vert \le 1$.

Since *f* is the weak limit of $(f_\alpha )$, for *g*, $h \in L^2(V)$ we have

$$\begin{aligned} \langle f_\alpha \otimes f_\alpha ^*, g \otimes h^*\rangle = (f_\alpha , g) (f_\alpha , h) \rightarrow (f, g) (f, h) = \langle f \otimes f^*, g \otimes h^*\rangle . \end{aligned}$$

Now, $G_\epsilon$ has finite rank for every $\epsilon > 0$, so we must have

$$\begin{aligned} \langle f_\alpha \otimes f_\alpha ^*, G_\epsilon \rangle \rightarrow \langle f \otimes f^*, G_\epsilon \rangle \end{aligned}$$

and, together with (11), it follows that ${\mathcal {B}}$ is weakly closed.

Now we only have to argue that $f \otimes f^*$ for $f \ge 0$ is an extreme point if and only if $f = 0$ or $\Vert f\Vert = 1$. First, if $0< \Vert f\Vert < 1$, then $f \otimes f^*$ is a convex combination of 0 and $\Vert f\Vert ^{-2} f \otimes f^*$, and hence not an extreme point.

Conversely, 0 is clearly not a convex combination of nonzero points, and hence it is an extreme point. Moreover, if $\Vert f\Vert = 1$, then $\Vert f \otimes f^*\Vert = 1$. Now, by the Cauchy–Schwarz inequality, it is impossible for a vector of norm 1 in $L^2$ to be a nontrivial convex combination of other vectors of norm 1, so $f \otimes f^*$ is an extreme point. $\square$

## When is the completely positive formulation exact?

*Throughout this section, the Haar measure on a compact group will always be normalized so the group has total measure* 1.

When is $\vartheta (G, {\mathcal {C}}(V)) = \alpha _\omega (G)$? When *G* is a finite graph and $\omega$ is the counting measure, equality holds, as we saw in the introduction. In the finite case, actually, equality holds irrespective of the measure. In this section, we will see some sufficient conditions on *G* and $\omega$ under which $\vartheta (G, {\mathcal {C}}(V)) = \alpha _\omega (G)$; these conditions will be satisfied by the main examples of infinite graphs considered here.

Let $G = (V, E)$ be a topological graph. An *automorphism* of *G* is a homeomorphism $\sigma :V \rightarrow V$ such that $(x, y) \in E$ if and only if $(\sigma x, \sigma y) \in E$. Denote by ${{\,\mathrm{Aut}\,}}(G)$ the set of all automorphisms of *G*, which is a group under function composition.

Say *V* is a set and $\Gamma$ a group that acts on *V*. We say that $\Gamma$ *acts continuously* on *V* if

(i) for every $\sigma \in \Gamma$, the map $x\mapsto \sigma x$ from *V* to *V* is continuous and
(ii) for every $x \in V$, the map $\sigma \mapsto \sigma x$ from $\Gamma$ to *V* is continuous.

We say that $\Gamma$ *acts transitively* on *V* if for all *x*, $y \in V$ there is $\sigma \in \Gamma$ such that $\sigma x = y$.

Assume that $\Gamma$ is compact and that it acts continuously and transitively on *V* and let $\mu$ be its Haar measure. Fix $x \in V$ and consider the function $p:\Gamma \rightarrow V$ such that $p(\sigma ) = \sigma x$. The *pushforward* of $\mu$ is the measure $\omega$ on *V* defined as follows: a set $X \subseteq V$ is measurable if $p^{-1}(X)$ is measurable and its measure is $\omega (X) = \mu (p^{-1}(X))$. The pushforward is a Borel measure; moreover, since $\Gamma$ acts transitively and since $\mu$ is invariant, it is independent of the choice of *x*. The pushforward is also invariant under the action of $\Gamma$, that is, if $X \subseteq V$ and $\sigma \in \Gamma$, then

$$\begin{aligned} \omega (\sigma X) = \omega (\{\, \sigma x : x \in X\,\}) = \omega (X). \end{aligned}$$

Let *V* be a metric space with metric *d* and $\omega$ be a Borel measure on *V* such that every open set has positive measure. A point *x* in a measurable set $S \subseteq V$ is a *density point* of *S* if

$$\begin{aligned} \lim _{\delta \downarrow 0} \frac{\omega (S \cap B(x, \delta ))}{\omega (B(x, \delta ))} = 1. \end{aligned}$$

We say that the metric *d* is a *density metric* for $\omega$ if for every measurable set $S \subseteq V$ the set of all density points of *S* has the same measure as *S*, that is, almost all points of *S* are density points. For example, Lebesgue’s density theorem states that the Euclidean metric on ${\mathbb {R}}^n$ is a density metric for the Lebesgue measure.

We now come to the main theorem of the paper.

### Theorem 5.1

Let $G = (V, E)$ be a locally independent graph where *V* is a compact Hausdorff space, $\Gamma \subseteq {{\,\mathrm{Aut}\,}}(G)$ be a compact group that acts continuously and transitively on *V*, and $\omega$ be a multiple of the pushforward of the Haar measure on $\Gamma$. If $\Gamma$ is metrizable via a bi-invariant density metric for the Haar measure, then $\vartheta (G, {\mathcal {C}}(V)) = \alpha _\omega (G)$.

Here, a *bi-invariant metric* on $\Gamma$ is a metric *d* such that for all $\lambda$, $\gamma$, $\sigma$, $\tau \in \Gamma$ we have $d(\lambda \sigma \gamma , \lambda \tau \gamma ) = d(\sigma , \tau )$.

Theorem 5.1 implies for instance that

$$\begin{aligned} \vartheta (G(S^{n-1}, \{\theta \}), {\mathcal {C}}(S^{n-1})) = \alpha _\omega (G(S^{n-1}, \{\theta \})) \end{aligned}$$

for every angle $\theta > 0$. Indeed, $G(S^{n-1}, \{\theta \})$ is a locally independent graph. For $\Gamma$ we take the orthogonal group $\mathrm {O}(n)$; this group acts continuously and transitively on $S^{n-1}$ and the surface measure on the sphere is a multiple of the pushforward of the Haar measure [29, Theorem 3.7]. The metric on $\mathrm {O}(n) \subseteq {\mathbb {R}}^{n \times n}$ inherited from the Euclidean metric is bi-invariant and is moreover a density metric since $\mathrm {O}(n)$ is a Riemannian manifold [15]. More generally, any compact Lie group is metrizable via a bi-invariant metric [31, Corollary 1.4].

In the proof of the theorem, the symmetry provided by the group $\Gamma$ is used to reduce the problem to an equivalent problem on a graph over $\Gamma$, a Cayley graph.

### Cayley graphs

Let $\Gamma$ be a topological group with identity 1 and $\Sigma \subseteq \Gamma$ be such that $1 \notin \Sigma$ and $\Sigma ^{-1} = \{\, \sigma ^{-1} : \sigma \in \Sigma \,\} = \Sigma$. Consider the graph whose vertex set is $\Gamma$ and in which $\sigma$, $\tau \in \Gamma$ are adjacent if and only if $\sigma ^{-1} \tau \in \Sigma$ (which happens, since $\Sigma ^{-1} = \Sigma$, if and only if $\tau ^{-1}\sigma \in \Sigma$). This is the *Cayley graph* over $\Gamma$ with *connection set* $\Sigma$; it is denoted by ${{\,\mathrm{Cayley}\,}}(\Gamma , \Sigma )$. Note that $\Gamma$ acts on itself continuously and transitively and that left multiplication by an element of $\Gamma$ is an automorphism of the Cayley graph.

We will use the following construction to relate a vertex-transitive graph to a Cayley graph over any transitive subgroup of its automorphism group. Let $G = (V, E)$ be a topological graph and $\Gamma \subseteq {{\,\mathrm{Aut}\,}}(G)$ be a group that acts transitively on *V*. Fix $x_0 \in V$ and set $\Sigma _{G,x_0} = \{\, \sigma \in \Gamma : (\sigma x_0, x_0) \in E\,\}$. Since $\Gamma \subseteq {{\,\mathrm{Aut}\,}}(G)$, we have $\Sigma _{G,x_0}^{-1} = \Sigma _{G,x_0}$.

#### Lemma 5.2

If $G = (V, E)$ is a locally independent graph and if $\Gamma \subseteq {{\,\mathrm{Aut}\,}}(G)$ is a topological group that acts continuously and transitively on *V*, then ${{\,\mathrm{Cayley}\,}}(\Gamma , \Sigma _{G,x_0})$ is locally independent for all $x_0 \in V$. If moreover $\omega$ is a multiple of the pushforward of the Haar measure $\mu$ on $\Gamma$, then for every $M \ge 0$ the graph *G* has a measurable independent set of measure at least *M* if and only if ${{\,\mathrm{Cayley}\,}}(\Gamma , \Sigma _{G,x_0})$ has a measurable independent set of measure at least $M / \omega (V)$; in particular,

$$\begin{aligned} \alpha _\mu ({{\,\mathrm{Cayley}\,}}(\Gamma , \Sigma _{G,x_0})) = \alpha _\omega (G) / \omega (V) \end{aligned}$$

for all $$x_0 \in V$$.

#### Proof

Independent sets in *G* and ${{\,\mathrm{Cayley}\,}}(\Gamma , \Sigma _{G,x_0})$ are related: if $p:\Gamma \rightarrow V$ is the function such that $p(\sigma ) = \sigma x_0$, then (i) if $I\subseteq V$ is independent, then so is $p^{-1}(I)$; conversely, (ii) if $I \subseteq \Gamma$ is independent, then so is *p*(*I*).

Let us first prove the second statement of the theorem. By normalizing $\omega$ if necessary, we may assume that $\omega (V) = 1$. Then $\omega$ is the pushforward of $\mu$, and (i) implies directly that if $I \subseteq V$ is a measurable independent set, then $p^{-1}(I) \subseteq \Gamma$ is a measurable independent set with $\mu (p^{-1}(I)) = \omega (I)$.

Now suppose $I \subseteq \Gamma$ is a measurable independent set. The Haar measure is inner regular, meaning that we can take a sequence $C_1$, $C_2$, ... of compact subsets of *I* such that $\mu (I {\setminus } C_n) < 1/n$. Let *C* be the union of all $C_n$. Since $C \subseteq I$, we have that *C*, and hence *p*(*C*), are both independent sets. Since $C_n$ is compact, $p(C_n)$ is also compact and hence measurable. But then since

$$\begin{aligned} p(C) = \bigcup _{n=1}^\infty p(C_n), \end{aligned}$$

it follows that *p*(*C*) is measurable. Finally, $\omega (p(C)) = \mu (p^{-1}(p(C))) \ge \mu (C) = \mu (I)$, as we wanted.

As for the first statement of the theorem, suppose *G* is locally independent and let $I \subseteq \Gamma$ be a compact independent set. The function *p* is continuous and hence $p(I) \subseteq V$ is compact. Since *G* is locally independent and *p*(*I*) is independent, there is an open independent set *S* in *G* that contains *p*(*I*). But then $p^{-1}(S)$ is an open independent set in ${{\,\mathrm{Cayley}\,}}(\Gamma , \Sigma _{G,x_0})$ that contains *I*, and thus the Cayley graph is locally independent. $\square$

The theta parameters of *G* and any corresponding Cayley graph are also related:

#### Lemma 5.3

If $G = (V, E)$ is a locally independent graph, if $\Gamma \subseteq {{\,\mathrm{Aut}\,}}(G)$ is a compact group that acts continuously and transitively on *V*, and if $\omega$ is a multiple of the pushforward of the Haar measure $\mu$ on $\Gamma$, then

$$\begin{aligned}\vartheta (G, {\mathcal {C}}(V)) / \omega (V) \le \vartheta ({{\,\mathrm{Cayley}\,}}(\Gamma , \Sigma _{G,x_0}), {\mathcal {C}}(\Gamma )) \end{aligned}$$

for all $$x_0 \in V$$.

In fact, there is nothing special about the cone ${\mathcal {C}}(V)$ in the above statement; the statement holds for any cone invariant under the action of $\Gamma$, for example the cone of positive kernels.

#### Proof

We may assume that $\omega (V) = 1$. Fix $x_0\in V$ and let $\Phi :L^2(V\times V) \rightarrow L^2(\Gamma \times \Gamma )$ be the operator such that

$$\begin{aligned} \Phi (A)(\sigma , \tau ) = A(\sigma x_0, \tau x_0) \end{aligned}$$

for all $\sigma$, $\tau \in \Gamma$. Since $\Gamma$ acts continuously on *V*, if *A* is continuous, then so is $\Phi (A)$. Moreover,

$$\begin{aligned} \int _\Gamma \Phi (A)(\sigma , \sigma )\, d\mu (\sigma ) = \int _V A(x, x)\, d\omega (x). \end{aligned}$$

Indeed,

12 $$\begin{aligned} \int _\Gamma \Phi (A)(\sigma , \sigma )\, d\mu (\sigma ) = \int _\Gamma A(\sigma x_0, \sigma x_0)\, d\mu (\sigma ). \end{aligned}$$

Now, the right-hand side above is independent of $x_0$. For if $x_0' \ne x_0$, then since $\Gamma$ acts transitively on *V* there is $\tau \in \Gamma$ such that $x_0' = \tau x_0$. Then using the right invariance of the Haar measure we get

$$\begin{aligned} \int _\Gamma A(\sigma x_0', \sigma x_0')\, d\mu (\sigma ) = \int _\Gamma A(\sigma \tau x_0, \sigma \tau x_0)\, d\mu (\sigma ) = \int _\Gamma A(\sigma x_0, \sigma x_0)\, d\mu (\sigma ). \end{aligned}$$

The measure $\omega$ is the pushforward of $\mu$, so it is invariant under the action of $\Gamma$ and $\omega (V) = 1$. Continuing (12) we get

$$\begin{aligned} \begin{aligned} \int _\Gamma A(\sigma x_0, \sigma x_0)\, d\mu (\sigma )&= \int _V \int _\Gamma A(\sigma x, \sigma x)\, d\mu (\sigma ) d\omega (x)\\&=\int _\Gamma \int _V A(\sigma x, \sigma x)\, d\omega (x) d\mu (\sigma )\\&=\int _V A(x, x)\, d\omega (x), \end{aligned} \end{aligned}$$

as we wanted. Similarly, one can prove that $\langle \Phi (A), \Phi (B)\rangle = \langle A, B\rangle$; in particular, for all *A*, $B \in L^2(V \times V)$ we have $\Vert \Phi (A)\Vert = \Vert A\Vert$ and we see that $\Phi$ is a bounded operator.

Now let *A* be a feasible solution of $\vartheta (G, {\mathcal {C}}(V))$. Claim: $\Phi (A)$ is a feasible solution of $\vartheta ({{\,\mathrm{Cayley}\,}}(\Gamma , \Sigma _{G,x_0}), {\mathcal {C}}(\Gamma ))$.

Indeed, $\int _\Gamma \Phi (A)(\sigma , \sigma )\, d\mu (\sigma ) = 1$. If $\sigma$, $\tau \in \Gamma$ are adjacent in the Cayley graph, then $(\sigma x_0, \tau x_0) \in E$, so that $\Phi (A)(\sigma , \tau ) = A(\sigma x_0, \tau x_0) = 0$. So it remains to show that $\Phi (A) \in {\mathcal {C}}(\Gamma )$.

Note *A* is the limit, in the norm topology, of a sequence $(A_n)$, where each $A_n$ is a finite sum of kernels of the form $f \otimes f^*$ with $f \in L^2(V)$ nonnegative. Since $\Phi$ is linear and since $\Phi (f \otimes f^*) \in {\mathcal {C}}(\Gamma )$ for all nonnegative $f \in L^2(V)$, we have $\Phi (A_n) \in {\mathcal {C}}(\Gamma )$ for all *n*. Now $\Vert \Phi (A_n - A)\Vert = \Vert A_n - A\Vert$, so $\Phi (A)$ is the limit of $(\Phi (A_n))$, and hence $\Phi (A) \in {\mathcal {C}}(\Gamma )$, proving the claim.

Finally, $\langle J, \Phi (A)\rangle = \langle \Phi (J), \Phi (A)\rangle = \langle J, A\rangle$, and since *A* is any feasible solution of $\vartheta (G, {\mathcal {C}}(V))$, the theorem follows. $\square$

### The Reynolds operator

Let *V* be a compact Hausdorff space, let $\Gamma$ be a compact group that acts continuously and transitively on *V*, and consider on *V* a multiple of the pushforward of the Haar measure $\mu$ on $\Gamma$. An important tool in the proof of Theorem 5.1 will be the *Reynolds operator* $R:L^2(V \times V) \rightarrow L^2(V \times V)$ that maps a kernel to its symmetrization: for $A \in L^2(V \times V)$,

$$\begin{aligned} R(A)(x, y) = \int _\Gamma A(\sigma x, \sigma y)\, d\mu (\sigma ) \end{aligned}$$

almost everywhere^3^ in $V \times V$. The operator is defined given a group that acts on *V*; the group and its action will always be clear from context. Since $\Gamma$ is compact and therefore the Haar measure is both left and right invariant, the Reynolds operator is self adjoint, that is, $\langle R(A), B\rangle = \langle A, R(B)\rangle$.

#### Lemma 5.4

If *V* is a compact space, if $\Gamma$ is a compact group that acts continuously and transitively on *V*, and if *V* is metrizable via a $\Gamma$-invariant metric, then for every continuous $A:V \times V \rightarrow {\mathbb {R}}$ the kernel $R(A)$ is also continuous.

Here we say that a metric *d* on *V* is $\Gamma$ *-invariant* if $d(\sigma x, \sigma y) = d(x, y)$ for all *x*, $y \in V$ and $\sigma \in \Gamma$.

#### Proof

If *d* is a $\Gamma$-invariant metric on *V*, then

$$\begin{aligned} d((x, y), (x', y')) = \max \{ d(x, x'), d(y, y') \} \end{aligned}$$

is a metric inducing the product topology on $V \times V$. Now *A* is continuous, and hence uniformly continuous on the compact metric space $V \times V$. So for every $\epsilon > 0$ there is $\delta > 0$ such that for all (*x*, *y*), $(x', y') \in V \times V$,

$$\begin{aligned} \text {if }d((x, y), (x', y'))< \delta , \text { then }|A(x, y) - A(x', y')| < \epsilon . \end{aligned}$$

Since *d* is $\Gamma$-invariant, $d((\sigma x, \sigma y), (\sigma x', \sigma y')) = d((x, y), (x', y'))$, and

13 $$\begin{aligned} \text {if}~d((x, y), (x', y'))< \delta , \text { then } |A(\sigma x, \sigma y) - A(\sigma x', \sigma y')| < \epsilon \text { for all}~\sigma \in \Gamma .\nonumber \\ \end{aligned}$$

So, given $\epsilon > 0$, if $\delta > 0$ is such that (13) holds, then $d((x, y), (x', y')) < \delta$ implies that

$$\begin{aligned} |R(A)(x, y) - R(A)(x', y')| \le \int _\Gamma |A(\sigma x, \sigma y) - A(\sigma x', \sigma y')|\, d\mu (\sigma ) < \epsilon , \end{aligned}$$

proving that *R*(*A*) is continuous. $\square$

#### Lemma 5.5

If *V* is a compact space, if $\Gamma$ is a compact group that acts continuously and transitively on *V*, if *V* is metrizable via a $\Gamma$-invariant metric, and if on *V* we consider a multiple $\omega$ of the pushforward of the Haar measure on $\Gamma$, then for every $f \in L^2(V)$ the kernel $R(f \otimes f^*)$ is continuous.

#### Proof

By normalizing $\omega$ if necessary, we may assume that $\omega (V) = 1$. Fix $x \in V$. Given a function $f \in L^2(V)$, consider the function $\phi :\Gamma \rightarrow {\mathbb {R}}$ such that $\phi (\sigma ) = f(\sigma x)$; given $g \in L^2(V)$, define $\psi :\Gamma \rightarrow {\mathbb {R}}$ similarly. Then

14 $$\begin{aligned} (f, g) = (\phi , \psi ), \end{aligned}$$

where $({\cdot }, {\cdot })$ denotes the usual $L^2$ inner product in the respective spaces; this implies in particular that $\phi$, $\psi \in L^2(\Gamma )$. To see (14) note that, since $\Gamma$ acts transitively, for every $x' \in V$ there is $\tau \in \Gamma$ such that $x = \tau x'$. Then use the invariance of the Haar measure to get

$$\begin{aligned} \int _\Gamma f(\sigma x') g(\sigma x')\, d\mu (\sigma ) = \int _\Gamma f(\sigma \tau x') g(\sigma \tau x')\, d\mu (\sigma ) = \int _\Gamma f(\sigma x) g(\sigma x)\, d\mu (\sigma ) = (\phi , \psi ). \end{aligned}$$

So, using the invariance of $\omega$ under the action of $\Gamma$,

$$\begin{aligned} (\phi , \psi ) = \int _V \int _\Gamma f(\sigma x) g(\sigma x)\, d\mu (\sigma ) d\omega (x) = \int _\Gamma \int _V f(\sigma x) g(\sigma x)\, d\omega (x) d\mu (\sigma ) = (f, g), \end{aligned}$$

as we wanted.

Assume without loss of generality that $\Vert f\Vert \le 1$. Continuous functions are dense in $L^2(V)$, so given $\epsilon > 0$ there is a continuous function *g* such that $\Vert f - g\Vert < \epsilon$. Then, for *x*, $y \in V$,

$$\begin{aligned} \begin{aligned}&\biggl |\int _\Gamma f(\sigma x) f(\sigma y) - g(\sigma x) g(\sigma y)\, d\mu (\sigma )\biggr |\\&\qquad =\biggl |\int _\Gamma f(\sigma x) f(\sigma y) - g(\sigma x) f(\sigma y) + g(\sigma x) f(\sigma y) - g(\sigma x) g(\sigma y)\, d\mu (\sigma )\biggr |\\&\qquad \le \int _\Gamma |f(\sigma x) - g(\sigma x)| |f(\sigma y)|\, d\mu (\sigma ) + \int _\Gamma |g(\sigma x)| |f(\sigma y) - g(\sigma y)|\, d\mu (\sigma ). \end{aligned} \end{aligned}$$

Since $\Vert f\Vert \le 1$, and hence $\Vert g\Vert \le 1 + \epsilon$, the Cauchy–Schwarz inequality together with (14) implies that the right-hand side above is less than $\epsilon + (1 + \epsilon )\epsilon$. So

$$\begin{aligned} |R(f \otimes f^*)(x, y) - R(g \otimes g^*)(x, y)| < \epsilon + (1 + \epsilon )\epsilon \end{aligned}$$

for all *x*, $$y \in V$$.

Now $g \otimes g^*$ is continuous, so Lemma 5.4 says that $R(g \otimes g^*)$ is continuous. With the above inequality, this implies that $R(f \otimes f^*)$ is the uniform limit of continuous functions, and hence continuous. $\square$

### Proof of Theorem 5.1

Under the hypotheses of Theorem 5.1, we must establish the identity $\vartheta (G, {\mathcal {C}}(V)) = \alpha _\omega (G)$. The ‘$\ge$’ inequality follows from Theorem 3.1; for the reverse inequality we use the following lemma.

#### Lemma 5.6

Let $G = (V, E)$ be a locally independent graph where *V* is a compact Hausdorff space, let $\Gamma \subseteq {{\,\mathrm{Aut}\,}}(G)$ be a compact group that acts continuously and transitively on *V*, let $\omega$ be a multiple of the pushforward of the Haar measure on $\Gamma$, and assume $\Gamma$ is metrizable via a bi-invariant density metric for the Haar measure. If *A* is a feasible solution of $\vartheta (G, {\mathcal {C}}(V))$, then there is a measurable independent set in *G* with measure at least $\langle J, A\rangle$.

#### Proof

In view of Lemmas 5.2 and 5.3, it is sufficient to prove that, if $\Sigma \subseteq \Gamma$ is a connection set such that ${{\,\mathrm{Cayley}\,}}(\Gamma , \Sigma )$ is a locally independent graph and if *A* is a feasible solution of $\vartheta ({{\,\mathrm{Cayley}\,}}(\Gamma , \Sigma ), {\mathcal {C}}(\Gamma ))$, then there is an independent set in ${{\,\mathrm{Cayley}\,}}(\Gamma , \Sigma )$ of measure at least $\langle J, A\rangle$.

So fix a connection set $\Sigma \subseteq \Gamma$ and suppose ${{\,\mathrm{Cayley}\,}}(\Gamma , \Sigma )$ is locally independent. Throughout the rest of the proof, $E_\Sigma$ will be the edge set of ${{\,\mathrm{Cayley}\,}}(\Gamma , \Sigma )$. It is immediate that

$$\begin{aligned} \vartheta ({{\,\mathrm{Cayley}\,}}(\Gamma , \Sigma ), {\mathcal {C}}(\Gamma )) = \vartheta ((\Gamma , E_\Sigma ), {\mathcal {C}}(\Gamma )) = \vartheta ((\Gamma , {{\,\mathrm{cl}\,}}E_\Sigma ), {\mathcal {C}}(\Gamma )), \end{aligned}$$

that is, considering the closure of the edge set does not change the optimal value. Together with Theorem 2.3, this implies that we may assume that $E_\Sigma$ is closed.

Notice that $\Gamma$ is a Hausdorff space (topological groups are Hausdorff spaces by definition) and that $\mu$ is an inner-regular Borel measure (because it is a Haar measure) that is positive on open sets (indeed, if $S \subseteq \Gamma$ is open, then $\{\,\sigma S : \sigma \in \Gamma \,\}$ is an open cover of $\Gamma$; since $\Gamma$ is compact, there is a finite subcover, hence $\mu (S) > 0$ or else we would have $\mu (\Gamma ) = 0$). So we can use the results of Sect. 4.

There is a countable set $E' \subseteq E_\Sigma$ such that ${{\,\mathrm{cl}\,}}E' = E_\Sigma$. Indeed, since $E_\Sigma$ is closed and hence compact, for every $n \ge 1$ we can cover $E_\Sigma$ with finitely many open balls of radius 1/*n*; now choose one point of $E_\Sigma$ in each such ball and let $E'$ be the set of all points chosen for $n = 1$, 2, ....

Let $(\sigma _1, \tau _1)$, $(\sigma _2, \tau _2)$, ... be an enumeration of $E'$. For $n \ge 1$ consider the kernel

$$\begin{aligned} T_n = \sum _{i=1}^\infty 2^{-i} \mu (B(\sigma _i, 1/n))^{-1} \mu (B(\tau _i, 1/n))^{-1} \chi _{B(\sigma _i, 1/n) \times B(\tau _i, 1/n)}. \end{aligned}$$

This is indeed a kernel: the norm of each summand is $2^{-i}$ times a constant that depends only on *n*, so $T_n$ is square integrable.

If $A:\Gamma \times \Gamma \rightarrow {\mathbb {R}}$ is continuous, and hence uniformly continuous, then for every $\epsilon > 0$ there is $n_0$ such that for all $n \ge n_0$ we have

$$\begin{aligned} |A(\sigma , \tau ) - A(\sigma _i, \tau _i)| < \epsilon \qquad \text {for all}~i \ge 1, \sigma \in B(\sigma _i, 1/n), \text {and}~\tau \in B(\tau _i, 1/n). \end{aligned}$$

This implies that

15 $$\begin{aligned} \lim _{n\rightarrow \infty } \langle T_n, A\rangle = \sum _{i=1}^\infty 2^{-i} A(\sigma _i, \tau _i). \end{aligned}$$

Let *A* be a feasible solution of $\vartheta ({{\,\mathrm{Cayley}\,}}(\Gamma , \Sigma ), {\mathcal {C}}(\Gamma ))$. Since ${{\,\mathrm{tr}\,}}A = 1$, Theorem 4.8 tells us that $A \in {\mathcal {T}}(\Gamma )$, where ${\mathcal {T}}(\Gamma )$ is the tip of ${\mathcal {C}}(\Gamma )$; see Sect. 4.3. Also from Sect. 4.3 we know that ${\mathcal {T}}(\Gamma )$ is weakly compact, that it is a subset of $L^2(\Gamma \times \Gamma )$, whose weak topology is locally convex, and that the weak topology on ${\mathcal {T}}(\Gamma )$ is metrizable.^4^ So we can apply Choquet’s theorem [43, Theorem 10.7] to get a probability measure $\nu$ on ${\mathcal {T}}(\Gamma )$ with barycenter *A* and $\nu ({\mathcal {X}}) = 1$, where ${\mathcal {X}}$ is the set of extreme points of ${\mathcal {T}}(\Gamma )$. From Theorem 4.9 we know that any element of ${\mathcal {X}}$ is of the form $f\otimes f^*$ for some nonnegative $f \in L^2(\Gamma )$ that is either 0 or such that $\Vert f\Vert = 1$. So *A* being the barycenter of $\nu$ means that for every $K \in L_\mathrm {sym}^2(\Gamma \times \Gamma )$ we have

16 $$\begin{aligned} \langle K, A\rangle = \int _{\mathcal {X}}\langle K, f \otimes f^*\rangle \, d\nu (f \otimes f^*). \end{aligned}$$

Since *A* is feasible, its symmetrization $R(A)$ is also feasible, and in particular $R(A)(\sigma , \tau ) = 0$ for all $(\sigma , \tau ) \in E_\Sigma$. (Note that here we need to use Lemma 5.4, and for that we need the left invariance of the metric on $\Gamma$.) This, together with (15), (16), and the self-adjointness of the Reynolds operator gives

$$\begin{aligned} \begin{aligned} 0&= \lim _{n\rightarrow \infty } \langle T_n, R(A)\rangle \\&=\lim _{n\rightarrow \infty } \langle R(T_n), A\rangle \\&=\lim _{n\rightarrow \infty } \int _{\mathcal {X}}\langle R(T_n), f \otimes f^*\rangle \, d\nu (f \otimes f^*)\\&=\lim _{n\rightarrow \infty } \int _{\mathcal {X}}\langle T_n, R(f \otimes f^*)\rangle \, d\nu (f \otimes f^*). \end{aligned} \end{aligned}$$

Fatou’s lemma now says that we can exchange the integral with the limit (that becomes a $\liminf$) to get

$$\begin{aligned} 0 \ge \int _{\mathcal {X}}\liminf _{n\rightarrow \infty }\langle T_n, R(f \otimes f^*)\rangle \, d\nu (f \otimes f^*). \end{aligned}$$

So, since $T_n$ and all *f*s above are nonnegative, the set

$$\begin{aligned} \{\, f \otimes f^* : \liminf _{n\rightarrow \infty }\langle T_n, R(f \otimes f^*)\rangle > 0\,\} \end{aligned}$$

has measure 0 with respect to $\nu$.

Taking $K = J$ in (16), we see that we can choose $f \ge 0$ with $\Vert f\Vert = 1$ such that $\langle J, f \otimes f^*\rangle \ge \langle J, A\rangle$ and

$$\begin{aligned} \liminf _{n\rightarrow \infty }\langle T_n, R(f \otimes f^*)\rangle = 0. \end{aligned}$$

By Lemma 5.5, $R(f \otimes f^*)$ is continuous, and hence from (15) we see that *f* satisfies

$$\begin{aligned} \sum _{i=1}^\infty 2^{-i} R(f \otimes f^*)(\sigma _i, \tau _i) = 0. \end{aligned}$$

So it must be that $R(f \otimes f^*)(\sigma _i, \tau _i) = 0$ for all *i*, and hence $R(f \otimes f^*)(\sigma , \tau ) = 0$ for all $(\sigma , \tau ) \in E_\Sigma$.

We are now almost done. Let *I* be the set of density points in the support of *f* (note that $f \in L^2(\Gamma )$, so its support is not clearly defined; here it suffices to take, however, an arbitrary representative of the equivalence class of *f* and then its support). Claim: *I* is independent. Proof: Since $R(f \otimes f^*)(\sigma , \tau ) = 0$ for every $(\sigma , \tau ) \in E_\Sigma$, it suffices to show that if $\sigma$, $\tau \in I$, then $R(f \otimes f^*)(\sigma , \tau ) > 0$.

Since $\sigma$, $\tau \in I$ are density points, there is $\delta > 0$ such that

17 $$\begin{aligned} \frac{\mu (I \cap B(\sigma , \delta ))}{\mu (B(\sigma , \delta ))} \ge 2/3\qquad \text {and}\qquad \frac{\mu (I \cap B(\tau , \delta ))}{\mu (B(\tau , \delta ))} \ge 2/3. \end{aligned}$$

For $\zeta \in \Gamma$, write $N_\zeta = \{\,\gamma \in \Gamma : \gamma \zeta \in I\,\}$; note that $I = N_\zeta \zeta$. The right invariance of the metric on $\Gamma$ implies that $B(\zeta , \delta ) = B(1, \delta ) \zeta$ for all $\zeta \in \Gamma$ and $\delta > 0$. Then, using (17) and the invariance of $\mu$,

$$\begin{aligned} \begin{aligned} 1&\ge \mu (B(1, \delta ))^{-1} \mu ((N_\sigma \cup N_\tau ) \cap B(1, \delta ))\\&=\mu (B(1, \delta ))^{-1} (\mu (N_\sigma \cap B(1, \delta )) + \mu (N_\tau \cap B(1, \delta )) - \mu (N_\sigma \cap N_\tau \cap B(1, \delta )))\\&\ge 4/3 - \mu (B(1, \delta ))^{-1} \mu (N_\sigma \cap N_\tau \cap B(1, \delta )). \end{aligned} \end{aligned}$$

Hence $\mu (N_\sigma \cap N_\tau ) \ge \mu (N_\sigma \cap N_\tau \cap B(1, \delta )) \ge \mu (B(1, \delta )) / 3 > 0$. Finally, since $f(\gamma ) > 0$ for all $\gamma \in I$,

$$\begin{aligned} R(f \otimes f^*)(\sigma , \tau ) = \int _{N_\sigma \cap N_\tau } f(\gamma \sigma ) f(\gamma \tau )\, d\mu (\gamma ) > 0, \end{aligned}$$

proving the claim.

So *I* is independent; it remains to estimate its measure. Recall *I* has the same measure as the support of *f*. Since $\Vert f\Vert = 1$, if $\chi _\Gamma$ is the constant 1 function, then

$$\begin{aligned} \langle J, A\rangle \le \langle J, f \otimes f^*\rangle = (f, \chi _\Gamma )^2 = (f, \chi _I)^2 \le \Vert f\Vert ^2 \Vert \chi _I\Vert ^2 = \mu (I), \end{aligned}$$

proving the lemma. $\square$

#### Proof of Theorem 5.1

Theorem 3.1 says that $\vartheta (G, {\mathcal {C}}(V)) \ge \alpha _\omega (G)$. The reverse inequality follows directly from Lemma 5.6. $\square$

Notice that, if $\vartheta (G, {\mathcal {C}}(V))$ has an optimal solution, then Lemma 5.6 implies that the measurable independence number is attained, that is, there is a measurable independent set *I* with $\omega (I) = \alpha _\omega (G)$. This is the case, for instance, of the distance graph $G = G(S^{n-1}, \{\theta \})$ for $n \ge 3$. In this case, a convergence argument, akin to the one we will use in Sect. 10.2, can be used to show that $\vartheta (G, {\mathcal {C}}(V))$ has an optimal solution. This provides another proof of a result of DeCorte and Pikhurko [9].

## Distance graphs on the Euclidean space

Theorem 5.1 applies only to graphs on compact spaces, but thanks to a limit argument it can be extended to some graphs on ${\mathbb {R}}^n$; we will see now how to make this extension for distance graphs.

Let $D \subseteq (0, \infty )$ be a set of forbidden distances and consider the *D*-distance graph $G({\mathbb {R}}^n, D)$, where two vertices *x*, $y \in {\mathbb {R}}^n$ are adjacent if $\Vert x-y\Vert \in D$. To measure the size of an independent set in $G({\mathbb {R}}^n, D)$ we use the upper density. Given a Lebesgue-measurable set $X \subseteq {\mathbb {R}}^n$, its *upper density* is

$$\begin{aligned} {\bar{\delta }}(X) = \sup _{p\in {\mathbb {R}}^n}\limsup _{T\rightarrow \infty } \frac{{{\,\mathrm{vol}\,}}(X \cap (p + [-T, T]^n))}{{{\,\mathrm{vol}\,}}[-T,T]^n}, \end{aligned}$$

where ${{\,\mathrm{vol}\,}}$ is the Lebesgue measure. The *independence density* of $G({\mathbb {R}}^n, D)$ is

$$\begin{aligned} \alpha _{{\bar{\delta }}}(G({\mathbb {R}}^n, D)) = \sup \{\, {\bar{\delta }}(I) : I \subseteq {\mathbb {R}}^n \text { is Lebesgue-measurable and independent}\,\}. \end{aligned}$$

### Periodic sets and limits of tori

The key idea is to consider independent sets that are periodic. A set $X \subseteq {\mathbb {R}}^n$ is *periodic* if there is a lattice $\Lambda \subseteq {\mathbb {R}}^n$ whose action leaves *X* invariant, that is, $X + v = X$ for all $v \in \Lambda$; in this case we say that $\Lambda$ is a *periodicity lattice* of *X*. Given a lattice $\Lambda \subseteq {\mathbb {R}}^n$ spanned by vectors $u_1$, ..., $u_n$, its (strict) *fundamental domain* with respect to $u_1$, ..., $u_n$ is the set

$$\begin{aligned} F = \{\, \alpha _1 u_1 + \cdots + \alpha _n u_n : \hbox {} \alpha _i \in [-1/2, 1/2) \hbox { for all}~ i\, \}. \end{aligned}$$

A periodic set with periodicity lattice $\Lambda$ repeats itself in copies of *F* translated by vectors in $\Lambda$. We identify the torus ${\mathbb {R}}^n / \Lambda$ with the fundamental domain *F* of $\Lambda$, identifying a coset *S* with the unique $x \in F$ such that $S = x + \Lambda$. When speaking of an element $x \in {\mathbb {R}}^n / \Lambda$, it is always implicit that *x* is the unique representative of $x + \Lambda$ that lies in the fundamental domain.

Given a lattice $\Lambda \subseteq {\mathbb {R}}^n$, consider the graph $G({\mathbb {R}}^n / \Lambda , D)$ whose vertex set is the torus ${\mathbb {R}}^n / \Lambda$ and in which vertices *x*, $y \in {\mathbb {R}}^n / \Lambda$ are adjacent if there is $v \in \Lambda$ such that $\Vert x-y+v\Vert \in D$. Independent sets in $G({\mathbb {R}}^n / \Lambda , D)$ correspond to periodic independent sets in $G({\mathbb {R}}^n, D)$ with periodicity lattice $\Lambda$ and vice versa.

#### Lemma 6.1

If $D \subseteq (0, \infty )$ is closed and bounded, then $G({\mathbb {R}}^n / L{\mathbb {Z}}^n, D)$ is locally independent for every $L > 2\sup D$.

The hypothesis that *D* is bounded is essential: for instance, if $D = (1,\infty )$, then for every $L > 0$, any $x \in {\mathbb {R}}^n / L{\mathbb {Z}}^n$ would be adjacent to itself. When *D* is unbounded, however, a theorem of Furstenberg et al. [17] implies that $\alpha _{{\bar{\delta }}}(G({\mathbb {R}}^n, D)) = 0$, so this case is not really interesting.

Though the lemma is stated in terms of the lattice $L{\mathbb {Z}}^n$, a similar statement holds for any lattice $\Lambda$, as long as the shortest nonzero vectors have length greater than $2\sup D$. The lattice $L{\mathbb {Z}}^n$ is chosen here for concreteness and also because it is the lattice that will be used later on.

#### Proof

The torus ${\mathbb {R}}^n / L{\mathbb {Z}}^n$ is a metric space, for instance with the metric

18 $$\begin{aligned} d(x, y) = \inf _{v \in L{\mathbb {Z}}^n} \Vert x-y+v\Vert \end{aligned}$$

for *x*, $y \in {\mathbb {R}}^n / L{\mathbb {Z}}^n$. If *x*, *y* lie in the fundamental domain with respect to the canonical basis vectors, then $\Vert x-y\Vert _\infty < L$ and $\Vert x-y\Vert < L n^{1/2}$. So if $\Vert v\Vert _\infty \ge L + L n^{1/2}$, then $\Vert x-y+v\Vert \ge \Vert x-y+v\Vert _\infty > L n^{1/2}$. This shows that the infimum above is attained by one of the finitely many vectors $v \in {\mathbb {R}}^n / L{\mathbb {Z}}^n$ with $\Vert v\Vert _\infty < L + L n^{1/2}$.

Let $L > 2\sup D$. Since any nonzero $v \in L{\mathbb {Z}}^n$ is such that $\Vert v\Vert \ge L$, the graph $G = G({\mathbb {R}}^n / L{\mathbb {Z}}^n, D)$ is loopless. We show that *x*, $y \in {\mathbb {R}}^n / L{\mathbb {Z}}^n$ are adjacent in *G* if and only if $d(x, y) \in D$, so *G* is a distance graph. Since *D* is closed, this will moreover imply that the edge set of *G* is closed and then, since the torus is metrizable, from Theorem 2.2 it will follow that *G* is locally independent.

If $d(x, y) \in D$, then immediately we have that *x*, *y* are adjacent. So suppose that *x*, *y* are adjacent, that is, that there is $v \in L{\mathbb {Z}}^n$ such that $\Vert x-y+v\Vert \in D$. Claim: $d(x, y) = \Vert x-y+v\Vert$. Indeed, take $w \in {\mathbb {R}}^n / L{\mathbb {Z}}^n$, $w \ne v$. Note that $\Vert x-y+v\Vert _\infty \le \Vert x-y+v\Vert \le \sup D < L / 2$ and that $\Vert w-v\Vert _\infty \ge L$. So

$$\begin{aligned} \Vert x-y+w\Vert \ge \Vert x-y+w\Vert _\infty = \Vert x-y+v+(w-v)\Vert _\infty > L / 2, \end{aligned}$$

proving the claim. $\square$

The independence numbers of the graphs $G({\mathbb {R}}^n / L{\mathbb {Z}}^n, D)$ are also related to the independence density of $G({\mathbb {R}}^n, D)$:

#### Lemma 6.2

If $D \subseteq (0, \infty )$ is bounded, then

$$\begin{aligned} \limsup _{L\rightarrow \infty } \frac{\alpha _{{{\,\mathrm{vol}\,}}}(G({\mathbb {R}}^n / L{\mathbb {Z}}^n, D))}{{{\,\mathrm{vol}\,}}({\mathbb {R}}^n / L{\mathbb {Z}}^n)} = \alpha _{{\bar{\delta }}}(G({\mathbb {R}}^n, D)), \end{aligned}$$

where ${{\,\mathrm{vol}\,}}$ denotes the Lebesgue measure.

It is well known that the densities of periodic sphere packings approximate the sphere-packing density arbitrarily well [7, Appendix A]. The proof of the lemma above is very similar to the proof of this fact.

#### Proof

Any independent set in $G({\mathbb {R}}^n / L{\mathbb {Z}}^n, D)$ gives rise to a periodic independent set in $G({\mathbb {R}}^n, D)$, so the ‘$\le$’ inequality is immediate. Let us then prove the reverse inequality.

If $D = \emptyset$, the statement is trivial. So assume $D \ne \emptyset$, write $r = \sup D$, and let $I \subseteq {\mathbb {R}}^n$ be a measurable independent set. From the definition of upper density, for every $\epsilon > 0$ there is a point $p \in {\mathbb {R}}^n$ such that for every $L_0 \ge 0$ there is $L \ge L_0$ with

19 $$\begin{aligned} \biggl |\frac{{{\,\mathrm{vol}\,}}(I \cap (p + [-L/2, L/2]^n))}{{{\,\mathrm{vol}\,}}[-L/2,L/2]^n} - {\bar{\delta }}(I)\biggr | < \epsilon / 2. \end{aligned}$$

Now take $L > 2r$ satisfying (19) and write $X = I \cap (p + [-L/2 + r, L/2 - r]^n)$; in words, *X* is obtained from $I \cap (p + [-L/2, L/2]^n)$ by erasing a border of width *r* around the facets of the hypercube. Then consider the set

$$\begin{aligned} I' = \bigcup _{v \in L{\mathbb {Z}}^n} X + v. \end{aligned}$$

The set $I'$ is, by construction, periodic with periodicity lattice $L{\mathbb {Z}}^n$, measurable, and independent. If moreover we take *L* large enough compared to *r*, then the volume of the border that was erased is negligible compared to the volume of the hypercube, and so using (19) we can make sure that $|{\bar{\delta }}(I') - {\bar{\delta }}(I)| < \epsilon$. Since *I* is an arbitrary measurable independent set, we just proved that for any $\epsilon > 0$ and any $L_0 \ge 0$ there is $L \ge L_0$ such that

$$\begin{aligned} \biggl |\frac{\alpha _{{{\,\mathrm{vol}\,}}}(G({\mathbb {R}}^n / L{\mathbb {Z}}^n, D))}{{{\,\mathrm{vol}\,}}({\mathbb {R}}^n / L{\mathbb {Z}}^n)} - \alpha _{{\bar{\delta }}}(G({\mathbb {R}}^n, D))\biggr | < \epsilon , \end{aligned}$$

establishing the reverse inequality. $\square$

### Some harmonic analysis

This is a good place to gather some notation and basic facts about harmonic analysis, which will be used next to extend Theorem 5.1 to $G({\mathbb {R}}^n, D)$; harmonic analysis will again be used in Sects. 9 and 10. For background, see e.g. the book by Reed and Simon [38]. *In this section, functions are complex-valued unless stated otherwise.*

A function $f \in L^\infty ({\mathbb {R}}^n)$ is said to be of *positive type* if $f(x) = \overline{f(-x)}$ for all $x \in {\mathbb {R}}^n$ and if for every $\rho \in L^1({\mathbb {R}}^n)$ we have

$$\begin{aligned} \int _{{\mathbb {R}}^n} \int _{{\mathbb {R}}^n} f(x-y) \rho (x) \overline{\rho (y)}\, dy dx \ge 0. \end{aligned}$$

A continuous function $f:{\mathbb {R}}^n \rightarrow {\mathbb {C}}$ is of positive type if and only if for every finite $U \subseteq {\mathbb {R}}^n$ the matrix

$$\begin{aligned} \bigl (f(x - y)\bigr )_{x, y \in U} \end{aligned}$$

is (Hermitian) positive semidefinite. This characterization shows that if *f* is a continuous function of positive type, then $\Vert f\Vert _\infty = f(0)$, since for every $x \in {\mathbb {R}}^n$ the matrix

$$\begin{aligned} \begin{pmatrix} f(0)&{}\quad f(x)\\ f(-x)&{}\quad f(0) \end{pmatrix} \end{aligned}$$

is positive semidefinite and hence $|f(x)| \le f(0)$. The set of all functions of positive type is a closed and convex cone, which we denote by ${{\,\mathrm{PSD}\,}}({\mathbb {R}}^n)$.

Bochner’s theorem says that functions of positive type are exactly the Fourier transforms of finite measures: a continuous function $f:{\mathbb {R}}^n \rightarrow {\mathbb {C}}$ is of positive type if and only if

20 $$\begin{aligned} f(x) = \int _{{\mathbb {R}}^n} e^{iu\cdot x}\, d\nu (u) \end{aligned}$$

for some finite (positive) Borel measure $\nu$, with the integral converging uniformly^5^ over ${\mathbb {R}}^n$.

A continuous function of positive type $f:{\mathbb {R}}^n \rightarrow {\mathbb {C}}$ has a well-defined *mean value*

$$\begin{aligned} M(f) = \lim _{T \rightarrow \infty } \frac{1}{{{\,\mathrm{vol}\,}}[-T,T]^n} \int _{[-T,T]^n} f(x)\, dx, \end{aligned}$$

and if $\nu$ is the measure in (20), then $M(f) = \nu (\{0\})$. To see this last identity, for $T > 0$ and $u \in {\mathbb {R}}^n$, write

$$\begin{aligned} g_T(u) = \frac{1}{{{\,\mathrm{vol}\,}}[-T, T]^n} \int _{[-T, T]^n} e^{i u \cdot x}\, dx. \end{aligned}$$

Let $g:{\mathbb {R}}^n \rightarrow {\mathbb {R}}$ be the function such that $g(0) = 1$ and $g(u) = 0$ for all nonzero $u \in {\mathbb {R}}^n$. Then *g* is the pointwise limit of $g_T$ as $T \rightarrow \infty$. Moreover, $|g_T(u)| \le 1$ for all *u*, and the constant one function is integrable with respect to the measure $\nu$, since $\nu$ is finite. So we may use Lebesgue’s dominated convergence theorem, and together with (20) we get

$$\begin{aligned} M(f) = \lim _{T \rightarrow \infty } \int _{{\mathbb {R}}^n} g_T(u)\, d\nu (u) = \int _{{\mathbb {R}}^n} g(u)\, d\nu (u) = \nu (\{0\}). \end{aligned}$$

A function $f:{\mathbb {R}}^n \rightarrow {\mathbb {C}}$ is *periodic* if there is a lattice $\Lambda \subseteq {\mathbb {R}}^n$ whose action leaves *f* invariant, that is, $f(x + v) = f(x)$ for all $x \in {\mathbb {R}}^n$ and $v \in \Lambda$; in this case we say that $\Lambda$ is a *periodicity lattice* of *f*. If *f* is periodic with periodicity lattice $\Lambda$, then

$$\begin{aligned} M(f) = \frac{1}{{{\,\mathrm{vol}\,}}({\mathbb {R}}^n / \Lambda )} \int _{{\mathbb {R}}^n / \Lambda } f(x)\, dx. \end{aligned}$$

So we may equip $L^2({\mathbb {R}}^n / \Lambda )$ with the inner product

$$\begin{aligned} (f, g) = {{\,\mathrm{vol}\,}}({\mathbb {R}}^n / \Lambda ) M(x \mapsto f(x) \overline{g(x)}). \end{aligned}$$

Then the functions $x\mapsto e^{i u \cdot x}$, for $u \in 2\pi \Lambda ^*$ where

$$\begin{aligned} \Lambda ^* = \{\, v \in {\mathbb {R}}^n : u \cdot v \in {\mathbb {Z}}\text { for all}~u \in \Lambda \,\} \end{aligned}$$

is the *dual lattice* of $\Lambda$, form a complete orthogonal system of $L^2({\mathbb {R}}^n / \Lambda )$. Given $f \in L^2({\mathbb {R}}^n / \Lambda )$ and $u \in 2\pi \Lambda ^*$, the *Fourier coefficient* of *f* at *u* is

$$\begin{aligned} {\widehat{f}}(u) = \frac{1}{{{\,\mathrm{vol}\,}}({\mathbb {R}}^n / \Lambda )} (f, x \mapsto e^{i u \cdot x}). \end{aligned}$$

We then have that

$$\begin{aligned} f(x) = \sum _{u\in 2\pi \Lambda ^*} {\widehat{f}}(u) e^{i u \cdot x} \end{aligned}$$

with convergence in $L^2$ norm, and from this follows *Parseval’s identity*: if *f*, $g \in L^2({\mathbb {R}}^n / \Lambda )$, then

$$\begin{aligned} (f, g) = \sum _{u \in 2\pi \Lambda ^*} {\widehat{f}}(u) \overline{{\widehat{g}}(u)}. \end{aligned}$$

### An exact completely positive formulation

Let $D \subseteq (0, \infty )$ be a set of forbidden distances and ${\mathcal {K}}({\mathbb {R}}^n) \subseteq {{\,\mathrm{PSD}\,}}({\mathbb {R}}^n)$ be a convex cone; consider the optimization problem

21 $$\begin{aligned} \begin{array}{r@{\ }l@{\quad }l} \text {maximize}&{}M(f)\\ &{}f(0) = 1,\\ &{}f(x) = 0\qquad \hbox { if}~\ \Vert x\Vert \in D,\\ &{}{f:{\mathbb {R}}^n \rightarrow {\mathbb {R}}\text { is continuous and}~f \in {\mathcal {K}}({\mathbb {R}}^n).} \end{array} \end{aligned}$$

We denote both the problem above and its optimal value by $\vartheta (G({\mathbb {R}}^n, D), {\mathcal {K}}({\mathbb {R}}^n))$. Notice that, since ${\mathcal {K}}({\mathbb {R}}^n) \subseteq {{\,\mathrm{PSD}\,}}({\mathbb {R}}^n)$, every $f \in {\mathcal {K}}({\mathbb {R}}^n)$ has a mean value, so the objective function is well defined.

Again, there are at least two cones that can be put in place of ${\mathcal {K}}({\mathbb {R}}^n)$. One is the cone ${{\,\mathrm{PSD}\,}}({\mathbb {R}}^n)$ of functions of positive type. The other is the cone of *real-valued completely positive functions* on ${\mathbb {R}}^n$, namely

$$\begin{aligned} {\mathcal {C}}({\mathbb {R}}^n) = {{\,\mathrm{cl}\,}}\{\, f \in L^\infty ({\mathbb {R}}^n): f \text { is real valued and continuous}\\ \text {and } \bigl (f(x-y)\bigr )_{x,y\in U} \in {\mathcal {C}}(U) \text { for all finite}~U \subseteq {\mathbb {R}}^n\,\}, \end{aligned}$$

where the closure is taken in the $L^\infty$ norm; note that ${\mathcal {C}}({\mathbb {R}}^n)$ is a cone contained in ${{\,\mathrm{PSD}\,}}({\mathbb {R}}^n)$.

#### Theorem 6.3

If $D \subseteq (0, \infty )$ is closed, then $\vartheta (G({\mathbb {R}}^n, D), {\mathcal {C}}({\mathbb {R}}^n)) = \alpha _{{\bar{\delta }}}(G({\mathbb {R}}^n, D))$.

Write $G = G({\mathbb {R}}^n, D)$ for short. Since *D* is closed and does not contain 0, Theorem 2.2 implies that *G* is locally independent. Recall that, if *D* is unbounded, then a theorem of Furstenberg et al. [17] implies that $\alpha _{{\bar{\delta }}}(G) = 0$. In this case, one can show that $\vartheta (G, {\mathcal {C}}({\mathbb {R}}^n)) = 0$; actually, $\vartheta (G, {{\,\mathrm{PSD}\,}}({\mathbb {R}}^n)) = 0$, as shown by Oliveira and Vallentin [36, Theorem 5.1] (see also Sect. 10 below).

To prove the theorem we may therefore assume that *D* is bounded and nonempty. Write $r = \sup D$, and for $L > 2r$ write $V_L = {\mathbb {R}}^n / L{\mathbb {Z}}^n$; note $V_L$ is a compact Abelian group. Lemma 6.1 says that $G_L = G(V_L, D)$ is locally independent. Since $V_L$ is metrizable via the bi-invariant metric (18), by taking $V = \Gamma = V_L$ and letting $\omega$ be the Lebesgue measure on $V_L$, the graph $G_L$ satisfies the hypotheses of Theorem 5.1, and so

$$\begin{aligned} \vartheta (G_L, {\mathcal {C}}(V_L)) = \alpha _{{{\,\mathrm{vol}\,}}}(G_L). \end{aligned}$$

Lemma 6.2 then implies that

22 $$\begin{aligned} \limsup _{L\rightarrow \infty } \frac{\vartheta (G_L, {\mathcal {C}}(V_L))}{{{\,\mathrm{vol}\,}}V_L} = \alpha _{{\bar{\delta }}}(G). \end{aligned}$$

So to prove Theorem 6.3 it suffices to show that the limit above is equal to $\vartheta (G, {\mathcal {C}}({\mathbb {R}}^n))$. The proof of this fact is a bit technical, but the main idea is simple; we prove the following two assertions:

1. If *A* is a feasible solution of $\vartheta (G_L, {\mathcal {C}}(V_L))$ for $L > 2r$, then there is a feasible solution *f* of $\vartheta (G, {\mathcal {C}}({\mathbb {R}}^n))$ such that $M(f) = ({{\,\mathrm{vol}\,}}V_L)^{-1} \langle J, A \rangle$.
2. If *f* is a feasible solution of $\vartheta (G, {\mathcal {C}}({\mathbb {R}}^n))$, then for every $L > 2r$ there is a feasible solution $A_L$ of $\vartheta (G_L, {\mathcal {C}}(V_L))$ and $({{\,\mathrm{vol}\,}}V_L)^{-1} \langle J, A_L\rangle \rightarrow M(f)$ as $L \rightarrow \infty$.

The first assertion establishes that the limit in (22) is $\le \vartheta (G, {\mathcal {C}}({\mathbb {R}}^n))$; the second assertion establishes the reverse inequality.

To prove (A1), fix $L > 2r$ and let *A* be a feasible solution of $\vartheta (G_L, {\mathcal {C}}(V_L))$. By applying the Reynolds operator to *A* if necessary, we may assume that *A* is invariant under the action of $V_L$, that is, $A(x + z, y + z) = A(x, y)$ for all *x*, *y*, $z \in V_L$. Indeed, if *A* is feasible, then $R(A)$ is also feasible, and to see this it suffices to show that $R(A)$ is continuous, since the other constraints are easily seen to be satisfied. But the continuity of $R(A)$ follows from Lemma 5.4, since $V_L$ is metrizable via the invariant metric (18).

Since *A* is invariant, there is a function $g:V_L \rightarrow {\mathbb {R}}$ such that

$$\begin{aligned} A(x, y) = g(x - y)\qquad \hbox {for all}~ x \hbox {,} y \in V_L. \end{aligned}$$

Then:

(i) *g* is continuous;
(ii) since $L > 2r$, if $x \in {\mathbb {R}}^n$ is such that $\Vert x\Vert \in D$, then *x* lies in the fundamental domain of $L{\mathbb {Z}}^n$ with respect to the canonical basis vectors, and so $g(x) = A(0, x) = 0$ since 0 and *x* are adjacent in $G_L$;
(iii) since $A \in {\mathcal {C}}(V_L)$, using Theorem 4.7 we see that $g \in {\mathcal {C}}({\mathbb {R}}^n)$;
(iv) since *A* is invariant, its diagonal is constant, and then since ${{\,\mathrm{tr}\,}}A = 1$ we have $g(0) = ({{\,\mathrm{vol}\,}}V_L)^{-1}$.

This all implies that $f = ({{\,\mathrm{vol}\,}}V_L) g$ is a feasible solution of $\vartheta (G, {\mathcal {C}}({\mathbb {R}}^n))$; all that is left to do is to compute *M*(*f*). Since *g* is periodic, its mean value is the integral of *g* on the fundamental domain *F* of the periodicity lattice divided by the volume of *F*, hence

$$\begin{aligned} \langle J, A\rangle = \int _{V_L} \int _{V_L} g(x-y)\, dy dx = \int _{V_L} \int _{V_L} g(y)\, dy dx = ({{\,\mathrm{vol}\,}}V_L)^2 M(g), \end{aligned}$$

and we get $M(f) = ({{\,\mathrm{vol}\,}}V_L) M(g) = ({{\,\mathrm{vol}\,}}V_L)^{-1} \langle J, A\rangle$, as we wanted.

To prove (A2), let *f* be a feasible solution of $\vartheta (G, {\mathcal {C}}({\mathbb {R}}^n))$ and fix $L > 2r$. Let $W_L = [-L/2, L/2]^n$ and consider the kernel $H:W_L \times W_L \rightarrow {\mathbb {R}}$ such that $H(x, y) = f(x - y)$. Note *H* is continuous and, since $f \in {\mathcal {C}}({\mathbb {R}}^n)$, using Theorem 4.7 we see that $H \in {\mathcal {C}}(W_L)$.

Let $W'_L = [-L/2 + r, L/2 - r]^n$ and consider the kernel $F:V_L \times V_L \rightarrow {\mathbb {R}}$ such that

$$\begin{aligned} F(x, y) = {\left\{ \begin{array}{ll} H(x, y)&{}\hbox {if}~ x \hbox {,} y \in W_L';\\ 0&{}\text {otherwise}. \end{array}\right. } \end{aligned}$$

If *x*, $y \in V_L$ are adjacent in $G_L$, then $F(x, y) = 0$. Indeed, if either *x* or *y* is not in $W'_L$, then $F(x, y) = 0$. If *x*, $y \in W'_L$, then $\Vert x-y\Vert _\infty \le L - 2r$ and, if $v \in L{\mathbb {Z}}^n$ is nonzero, then $\Vert v\Vert _\infty \ge L$ and $\Vert x-y+v\Vert _\infty \ge 2r > r$, whence $\Vert x-y+v\Vert \notin D$. But then if *x* and *y* are adjacent, we must have $\Vert x-y\Vert \in D$ and $F(x, y) = H(x, y) = f(x-y) = 0$.

Now *F* is not continuous, but $R(F)$ is; here is a proof. Since *H* is continuous and positive (recall $H \in {\mathcal {C}}(W_L)$), Mercer’s theorem says that there are continuous functions $\phi _i:W_L \rightarrow {\mathbb {R}}$ with $\Vert \phi _i\Vert = 1$ and numbers $\lambda _i \ge 0$ for $i = 1$, 2, ... such that $\sum _{i=1}^\infty \lambda _i < \infty$ and

$$\begin{aligned} H(x, y) = \sum _{i=1}^\infty \lambda _i \phi _i(x) \phi _i(y) = \sum _{i=1}^\infty \lambda _i (\phi _i \otimes \phi _i^*)(x, y) \end{aligned}$$

with absolute and uniform convergence over $W_L \times W_L$.

For $i = 1$, 2, ... define the function $\psi _i:V_L \rightarrow {\mathbb {R}}$ by setting

$$\begin{aligned} \psi _i(x) = {\left\{ \begin{array}{ll} \phi _i(x)&{}\hbox { if}\ x \in W'_L;\\ 0&{}\text {otherwise}. \end{array}\right. } \end{aligned}$$

Then

$$\begin{aligned} F(x, y) = \sum _{i=1}^\infty \lambda _i \psi _i(x) \psi _i(y) = \sum _{i=1}^\infty \lambda _i (\psi _i \otimes \psi _i^*)(x, y). \end{aligned}$$

We show now that the series

$$\begin{aligned} \sum _{i=1}^\infty \lambda _i R(\psi _i \otimes \psi _i^*)(x, y) \end{aligned}$$

converges absolutely and uniformly over $V_L \times V_L$ and, since $R(\psi _i \otimes \psi _i^*)$ is continuous by Lemma 5.5, this will imply that $R(F)$ is continuous.

For $u \in V_L$ and $\psi :V_L \rightarrow {\mathbb {R}}$, write $\psi _u$ for the function such that $\psi _u(x) = \psi (x + u)$. Then

$$\begin{aligned} R(\psi _i \otimes \psi _i^*)(x, y) = \frac{1}{{{\,\mathrm{vol}\,}}V_L} \int _{V_L} \psi _i(x+z) \psi _i(y+z)\, dz = \frac{1}{{{\,\mathrm{vol}\,}}V_L} ((\psi _i)_x, (\psi _i)_y). \end{aligned}$$

Now $$|((\psi _i)_x, (\psi _i)_y)| \le \Vert \psi _i\Vert ^2 \le \Vert \phi _i\Vert ^2 = 1$$, so

$$\begin{aligned} \sum _{i=1}^\infty |\lambda _i ((\psi _i)_x, (\psi _i)_y)| \le \sum _{i=1}^\infty \lambda _i < \infty , \end{aligned}$$

establishing absolute convergence. For uniform convergence, note that given $\epsilon > 0$ there is $m \ge 1$ such that $\sum _{i=m}^\infty \lambda _i < \epsilon$. But then

$$\begin{aligned} \sum _{i=m}^\infty |\lambda _i ((\psi _i)_x, (\psi _i)_y)| \le \sum _{i=m}^\infty \lambda _i < \epsilon , \end{aligned}$$

establishing uniform convergence and thus finishing the proof that $R(F)$ is continuous.

Now that we know that $R(F)$ is continuous, we can show that $R(F) \in {\mathcal {C}}(V_L)$. Indeed, since *H* is continuous and belongs to ${\mathcal {C}}(W_L)$, using Theorem 4.7 it is straightforward to show that, if $U \subseteq V_L$ is finite, then $F[U] \in {\mathcal {C}}(U)$ and hence also $R(F)[U] \in {\mathcal {C}}(U)$. But then, since $R(F)$ is continuous, Theorem 4.7 implies that $R(F) \in {\mathcal {C}}(V_L)$.

So far we can conclude that $A_L = ({{\,\mathrm{tr}\,}}R(F))^{-1} R(F)$ is a feasible solution of $\vartheta (G_L, {\mathcal {C}}(V_L))$. To estimate $\langle J, A_L\rangle$ we use the following fact.

#### Lemma 6.4

If $f:{\mathbb {R}}^n \rightarrow {\mathbb {C}}$ is continuous and of positive type, then

23 $$\begin{aligned} \lim _{T\rightarrow \infty } \frac{1}{({{\,\mathrm{vol}\,}}[-T, T]^n)^2} \int _{[-T, T]^n} \int _{[-T, T]^n} f(x-y)\, dy dx = M(f). \end{aligned}$$

#### Proof

The function $g:{\mathbb {R}}^n \times {\mathbb {R}}^n \rightarrow {\mathbb {C}}$ such that $g(x, y) = f(x - y)$ is continuous and of positive type. Indeed, let $\nu$ be the measure given by Bochner’s theorem such that (20) holds and consider the Borel measure $\mu$ on ${\mathbb {R}}^n \times {\mathbb {R}}^n$ such that

$$\begin{aligned} \mu (X) = \nu (\{\,u \in {\mathbb {R}}^n : (u, -u) \in X\,\}) \end{aligned}$$

for all measurable $X \subseteq {\mathbb {R}}^n \times {\mathbb {R}}^n$. Then $\mu$ is a finite measure and

$$\begin{aligned} g(x, y) = f(x-y) = \int _{{\mathbb {R}}^n} e^{i u \cdot (x-y)}\, d\nu (u) = \int _{{\mathbb {R}}^n \times {\mathbb {R}}^n} e^{i (u \cdot x + v \cdot y)}\, d\mu (u, v), \end{aligned}$$

so $\mu$ is the measure representing *g*. But then the left-hand side of (23) is $M(g) = \mu (\{(0, 0)\}) = \nu (\{0\}) = M(f)$. $\square$

Now note that

$$\begin{aligned} {{\,\mathrm{tr}\,}}R(F) = \int _{V_L} F(x, x)\, dx = ({{\,\mathrm{vol}\,}}W'_L) f(0) = {{\,\mathrm{vol}\,}}W'_L. \end{aligned}$$

Since *r* is fixed,

$$\begin{aligned} \lim _{L \rightarrow \infty } \frac{{{\,\mathrm{vol}\,}}W'_L}{{{\,\mathrm{vol}\,}}V_L} = 1. \end{aligned}$$

So using the lemma above we get

$$\begin{aligned} \begin{aligned} \lim _{L \rightarrow \infty } ({{\,\mathrm{vol}\,}}V_L)^{-1} \langle J, A_L\rangle&= \lim _{L \rightarrow \infty } \frac{1}{{{\,\mathrm{vol}\,}}V_L} \int _{V_L} \int _{V_L} A_L(x, y)\, dy dx\\&=\lim _{L \rightarrow \infty } \frac{1}{({{\,\mathrm{vol}\,}}V_L)({{\,\mathrm{vol}\,}}W'_L)} \int _{W'_L} \int _{W'_L} f(x-y)\, dy dx\\&=\lim _{L \rightarrow \infty } \frac{{{\,\mathrm{vol}\,}}W'_L}{{{\,\mathrm{vol}\,}}V_L} \frac{1}{({{\,\mathrm{vol}\,}}W'_L)^2} \int _{W'_L} \int _{W'_L} f(x-y)\, dy dx\\&=M(f), \end{aligned} \end{aligned}$$

finishing the proof of (A2). Here, the second identity follows from the definition of $A_L$ and the self-adjointness of the Reynolds operator.

#### Proof of Theorem 6.3

Follows from (A1) and (A2), proved above. $\square$

## The Boolean-quadratic cone and polytope

As was said in Sect. 1, one can use valid inequalities for ${\mathcal {C}}(V)$ to strengthen the upper bound provided by $\vartheta (G, {{\,\mathrm{PSD}\,}}(V))$. This is one of our goals: to obtain better upper bounds in some particular cases of interest, like the unit-distance graph on Euclidean space or distance graphs on the sphere.

From a practical standpoint, and for reasons that will become clear soon, instead of using valid inequalities for the completely positive cone, it is more convenient to use valid inequalities for the Boolean-quadratic cone. Given a nonempty finite set *V*, the *Boolean-quadratic cone* on *V* is

$$\begin{aligned} {{\,\mathrm{BQC}\,}}(V) = {{\,\mathrm{cone}\,}}\{\, f \otimes f^* : f:V\rightarrow \{0,1\}\,\}; \end{aligned}$$

notice that ${{\,\mathrm{BQC}\,}}(V) \subseteq {\mathcal {C}}(V)$. The dual cone of ${{\,\mathrm{BQC}\,}}(V)$ is

$$\begin{aligned} {{\,\mathrm{BQC}\,}}^*(V)&= \{\, Z:V \times V \rightarrow {\mathbb {R}}: Z \text { is symmetric}\\&\qquad \hbox {and} \ \langle Z, A\rangle \ge 0 \hbox { for all}~ A \in {{\,\mathrm{BQC}\,}}(V)\,\}. \end{aligned}$$

Now let *V* be a compact topological space and $\omega$ be a finite Borel measure on *V* and consider the cone

$$\begin{aligned} {{\,\mathrm{BQC}\,}}(V)&= {{\,\mathrm{cl}\,}}\{\, A \in L^2(V \times V) : A {\text { is continuous}}\\&\qquad {\text {and }} A[U] \in {{\,\mathrm{BQC}\,}}(U) {\text { for all finite}}~U \subseteq V\,\}, \end{aligned}$$

with the closure taken in the $L^2$-norm topology. In view of Theorem 4.7, if *V* is a compact Hausdorff space and $\omega$ is positive on open sets, then ${{\,\mathrm{BQC}\,}}(V) \subseteq {\mathcal {C}}(V)$.

Let *V* be a compact Hausdorff space and $\omega$ be a finite Borel measure on *V*. If $G = (V, E)$ is a locally independent graph, then since ${{\,\mathrm{BQC}\,}}(V) \subseteq {\mathcal {C}}(V)$ we have

$$\begin{aligned} \vartheta (G, {{\,\mathrm{BQC}\,}}(V)) \le \vartheta (G, {\mathcal {C}}(V)). \end{aligned}$$

If *V* is finite and $\omega$ is the counting measure, then recalling the proof of the inequality $\vartheta (G, {\mathcal {C}}(V)) \ge \alpha _\omega (G)$ given in Sect. 3 we immediately get

24 $$\begin{aligned} \vartheta (G, {{\,\mathrm{BQC}\,}}(V)) \ge \alpha _\omega (G). \end{aligned}$$

If *V* is infinite, it is not clear that (24) holds; at least the proof of Theorem 3.1 does not go through anymore: if $f:V \rightarrow {\mathbb {R}}$ is the continuous function approximating the characteristic function of the independent set, then in general it is not true that $\Vert f\Vert ^{-2} f \otimes f^* \in {{\,\mathrm{BQC}\,}}(V)$. If *G* and $\omega$ satisfy the hypotheses of Theorem 5.1, however, then (24) holds and we have:

### Theorem 7.1

Let $G = (V, E)$ be a locally independent graph where *V* is a compact Hausdorff space, $\Gamma \subseteq {{\,\mathrm{Aut}\,}}(G)$ be a compact group that acts continuously and transitively on *V*, and $\omega$ be a multiple of the pushforward of the Haar measure on $\Gamma$. If $\Gamma$ is metrizable via a bi-invariant density metric for the Haar measure, then $\vartheta (G, {{\,\mathrm{BQC}\,}}(V)) = \alpha _\omega (G)$.

The proof requires the use of the Reynolds operator on *V*, namely of Lemma 5.5. For this we need a $\Gamma$-invariant metric on *V*, whose existence is implied by the metrizability of $\Gamma$ via a bi-invariant metric, as shown by the following lemma.

### Lemma 7.2

Let *V* be a compact Hausdorff space and $\Gamma$ be a compact group that acts continuously and transitively on *V*. If $\Gamma$ is metrizable via a bi-invariant metric, then *V* is metrizable via a $\Gamma$-invariant metric.

### Proof

For $x \in V$, consider the map $p_x:\Gamma \rightarrow V$ such that $p_x(\sigma ) = \sigma x$; the continuous action of $\Gamma$ implies that $p_x$ is continuous for every $x \in V$. Since $\Gamma$ is compact and Hausdorff and *V* is Hausdorff, $p_x$ is a closed and proper map: images of closed sets are closed and preimages of compact sets are compact.

Let $d_\Gamma$ be a bi-invariant metric that induces the topology on $\Gamma$ and for $\sigma \in \Gamma$ and $\delta \ge 0$ let

$$\begin{aligned} {\overline{B}}_\Gamma (\sigma , \delta ) = \{\, \tau \in \Gamma : d_\Gamma (\sigma , \tau ) \le \delta \,\} \end{aligned}$$

be the closed ball in $\Gamma$ with center $\sigma$ and radius $\delta$. For *x*, $y \in V$, let

$$\begin{aligned} d_V(x, y) = \inf \{\, \delta : y \in p_x({\overline{B}}_\Gamma (1, \delta ))\,\} = \inf \{\, d_\Gamma (1, \sigma ) : \sigma \in \Gamma ,\ \sigma x = y\,\}. \end{aligned}$$

It is easy to show that $d_V$ is a $\Gamma$-invariant metric; we show now that it induces the topology on *V*.

To this end, for $x \in V$ consider the closed ball with center *x* and radius $\delta \ge 0$, namely

$$\begin{aligned} \begin{aligned} {\overline{B}}_V(x, \delta )&= \{\, y \in V : d_V(x, y) \le \delta \,\}\\&= \{\, \sigma x : \sigma \in \Gamma \text { and }d_\Gamma (1, \sigma ) \le \delta \,\}\\&= p_x({\overline{B}}_\Gamma (1, \delta )). \end{aligned} \end{aligned}$$

Notice that this ball is closed since ${\overline{B}}_\Gamma (1, \delta )$ is closed and $p_x$ is a closed map. We show now that the collection of finite unions of such balls is a base of closed sets of the topology on *V*, and it will follow that the metric $d_V$ induces the topology on *V*.

Let $X \subseteq V$ be a closed set and take $x \notin X$. Note $p_x^{-1}(X)$ and $p_x^{-1}(\{x\})$ are compact and disjoint, so

$$\begin{aligned} \delta = d_\Gamma (p_x^{-1}(X), p_x^{-1}(\{x\})) > 0. \end{aligned}$$

Since $p_x^{-1}(X)$ is compact, it can be covered by finitely many closed balls of radius $\delta / 2$, say ${\overline{B}}_\Gamma (\sigma _i, \delta / 2)$ with $\sigma _i \in p_x^{-1}(X)$ for $i = 1$, ..., *N*; moreover, by the definition of $\delta$, we have that $p_x^{-1}(\{x\})$ is disjoint from each such ball. But then

$$\begin{aligned} X \subseteq p_x(p_x^{-1}(X)) \subseteq \bigcup _{i=1}^N p_x({\overline{B}}_\Gamma (\sigma _i, \delta /2)) = \bigcup _{i=1}^N p_{\sigma _i x}({\overline{B}}_\Gamma (1, \delta /2)) = \bigcup _{i=1}^N {\overline{B}}_V(\sigma _i x, \delta /2) \end{aligned}$$

and $x \notin \bigcup _{i=1}^N {\overline{B}}_V(\sigma _i x, \delta /2)$. We have shown that, given any closed set $X \subseteq V$ and any $x \notin X$, there is a finite union of $d_V$-balls that contains *X* but not *x*, that is, finite unions of $d_V$-balls form a base of closed sets of the topology on *V*. $\square$

### Proof of Theorem 7.1

Since ${{\,\mathrm{BQC}\,}}(V) \subseteq {\mathcal {C}}(V)$, from Theorem 5.1 it suffices to show that (24) holds. So let $I \subseteq V$ be a measurable independent set with $\omega (I) > 0$ (such a set exists since *G* is locally independent and $\omega$ is positive on open sets) and consider the kernel $A = \omega (I)^{-1} R(\chi _I \otimes \chi _I^*)$. Using Lemma 7.2 we know that *V* is metrizable via a $\Gamma$-invariant metric, and then using Lemma 5.5 we see that *A* is continuous; it is also immediate that ${{\,\mathrm{tr}\,}}A = 1$ and $A(x, y) = 0$ if *x*, $y \in V$ are adjacent. Let us then show that $A \in {{\,\mathrm{BQC}\,}}(V)$.

Indeed, given a finite $U \subseteq V$, note that for any $Z \in {{\,\mathrm{BQC}\,}}^*(U)$, if $\mu$ is the Haar measure on $\Gamma$, then

$$\begin{aligned} \sum _{x,y \in U} Z(x, y) A(x, y) = \omega (I)^{-1} \int _\Gamma \sum _{x,y \in U} Z(x, y) \chi _I(\sigma x) \chi _I(\sigma y) \, d\mu (\sigma ) \ge 0, \end{aligned}$$

whence $A[U] \in {{\,\mathrm{BQC}\,}}(U)$. So *A* is a feasible solution of $\vartheta (G, {{\,\mathrm{BQC}\,}}(V))$ with $\langle J, A\rangle = \omega (I)$, establishing (24). $\square$

A corresponding result holds for the bound for distance graphs on ${\mathbb {R}}^n$, presented in Sect. 6, by considering the cone

$$\begin{aligned} {{\,\mathrm{BQC}\,}}({\mathbb {R}}^n)&= {{\,\mathrm{cl}\,}}\{\, f \in L^\infty ({\mathbb {R}}^n) : f \text { is real valued and continuous}\\&\quad \text {and } \bigl (f(x- y)\bigr )_{x,y \in U} \in {{\,\mathrm{BQC}\,}}(U) \text { for all finite}~U \subseteq V\,\}, \end{aligned}$$

with the closure taken in the $L^\infty$ norm. Note that ${{\,\mathrm{BQC}\,}}({\mathbb {R}}^n) \subseteq {\mathcal {C}}({\mathbb {R}}^n)$.

### Theorem 7.3

If $D \subseteq (0, \infty )$ is closed, then

$$\begin{aligned} \vartheta (G({\mathbb {R}}^n, D), {{\,\mathrm{BQC}\,}}({\mathbb {R}}^n)) = \alpha _{{\bar{\delta }}}(G({\mathbb {R}}^n, D)). \end{aligned}$$

### Proof

Recall from Sect. 6.3 that we may assume *D* is bounded. In view of Theorem 6.3, it then suffices to show that $\vartheta (G({\mathbb {R}}^n, D), {{\,\mathrm{BQC}\,}}({\mathbb {R}}^n))\ge \alpha _{{\bar{\delta }}}(G({\mathbb {R}}^n, D))$.

Let $I \subseteq {\mathbb {R}}^n$ be a measurable and periodic independent set with ${\bar{\delta }}(I) > 0$ (which exists since *D* is bounded) and consider the function $f:{\mathbb {R}}^n \rightarrow {\mathbb {R}}$ given by

$$\begin{aligned} f(x) = {\bar{\delta }}(I)^{-1} \lim _{T\rightarrow \infty } \frac{1}{{{\,\mathrm{vol}\,}}[-T,T]^n} \int _{[-T,T]^n} \chi _I(z) \chi _I(x+z)\, dz \end{aligned}$$

(notice the limit above exists since *I* is periodic). This function is continuous and satisfies $f(0) = 1$ and $f(x) = 0$ if $\Vert x\Vert \in D$, since if $\Vert x\Vert \in D$ then for all *z* we cannot have both *z* and $x+z \in I$. Moreover, $f \in {{\,\mathrm{BQC}\,}}({\mathbb {R}}^n)$: if $U \subseteq {\mathbb {R}}^n$ is finite and $Z \in {{\,\mathrm{BQC}\,}}^*(U)$, then

$$\begin{aligned} \begin{aligned}&\sum _{x,y \in U} Z(x, y) f(x-y)\\&\qquad ={\bar{\delta }}(I)^{-1} \lim _{T\rightarrow \infty } \frac{1}{{{\,\mathrm{vol}\,}}[-T,T]^n} \int _{[-T,T]^n} \sum _{x,y \in U} Z(x, y) \chi _I(z) \chi _I(x-y+z)\, dz\\&\qquad ={\bar{\delta }}(I)^{-1} \lim _{T\rightarrow \infty } \frac{1}{{{\,\mathrm{vol}\,}}[-T,T]^n} \int _{[-T,T]^n} \sum _{x,y \in U} Z(x, y) \chi _I(x+z) \chi _I(y+z)\, dz\\&\qquad \ge 0, \end{aligned} \end{aligned}$$

whence *f* is a feasible solution of $\vartheta (G({\mathbb {R}}^n, D), {{\,\mathrm{BQC}\,}}({\mathbb {R}}^n))$. We also have $M(f) = {\bar{\delta }}(I)$. Indeed, the characteristic function $\chi _I$ of *I* is periodic, say with periodicity lattice $\Lambda$. For $x \in {\mathbb {R}}^n$, consider the function $(\chi _I)_x$ such that $(\chi _I)_x(z) = \chi _I(x+z)$. Then it is easy to check that the Fourier coefficient of $(\chi _I)_x$ at *u* equals $e^{i u \cdot x} {\widehat{\chi }}_I(u)$, and thus Parseval’s identity gives us

$$\begin{aligned} f(x) = {\bar{\delta }}(I)^{-1} ((\chi _I)_x, \chi _I) = {\bar{\delta }}(I)^{-1} \sum _{u \in 2\pi \Lambda ^*} |{\widehat{\chi }}_I(u)|^2 e^{i u \cdot x}. \end{aligned}$$

From this it is clear that $M(f) = {\widehat{f}}(0) = {\bar{\delta }}(I)^{-1} |{\widehat{\chi }}_I(0)|^2 = {\bar{\delta }}(I)$, since ${\widehat{\chi }}_I(0) = {\bar{\delta }}(I)$.

To finish, note that *I* is any measurable and periodic independent set, so using Lemma 6.2 the theorem follows. $\square$

Theorem 7.1 tells us that any number of constraints of the form

$$\begin{aligned} \sum _{x, y \in U} Z(x, y) A(x, y) \ge 0, \end{aligned}$$

for finite $U \subseteq {\mathbb {R}}^n$ and $Z \in {{\,\mathrm{BQC}\,}}^*(U)$, can be added to $\vartheta (G, {{\,\mathrm{PSD}\,}}(V))$, and that the resulting problem still provides an upper bound for the independence number. Moreover, if all such constraints are added, then we obtain the independence number. Theorem 7.3 says the same for the independence density of $G({\mathbb {R}}^n, D)$.

The main advantage of using ${{\,\mathrm{BQC}\,}}(U)$ instead of ${\mathcal {C}}(U)$ is that the Boolean-quadratic cone in finite dimension is a polyhedral cone, so for finite *U* one is able to compute all (or at least some of) the facets of ${{\,\mathrm{BQC}\,}}(U)$, though the amount of work gets prohibitively large already for $|U| = 7$ [11, §30.6]. The better upper bounds described in Sects. 8 and 9 were obtained by the use of constraints based on such facets.

### Subgraph constraints

Constraints from subgraphs of $G({\mathbb {R}}^n, \{1\})$ played a central role in the computation of the best upper bounds for the independence density of the unit-distance graph [2, 22, 36].

Such *subgraph constraints* are as follows. Let $G = (V, E)$ be a locally independent graph and $\omega$ be a Borel measure on *V* and assume *G* and $\omega$ satisfy the hypotheses of Theorem 5.1. Let $U \subseteq V$ be finite and for every $x_0 \in V$ consider the inequality

25 $$\begin{aligned} \sum _{y \in U} A(x_0, y) \le \alpha (G[U]) A(x_0, x_0), \end{aligned}$$

where $A \in L^2(V \times V)$ is continuous and *G*[*U*] is the subgraph of *G* induced by *U*.

After adding any number of such constraints to $\vartheta (G, {{\,\mathrm{PSD}\,}}(V))$ we still get an upper bound for $\alpha _\omega (G)$. Indeed, if $I \subseteq V$ is a measurable independent set of positive measure, then $A = \omega (I)^{-1} R(\chi _I \otimes \chi _I^*)$ is continuous, positive, and such that ${{\,\mathrm{tr}\,}}A = 1$, $A(x, y) = 0$ if *x*, $y \in V$ are adjacent, and $\langle J, A\rangle = \omega (I)$ (recall the proof of Theorem 7.1). Moreover, since $A(x, x) = \omega (V)^{-1}$ for all $x \in V$, and since for every $\sigma \in \Gamma \subseteq {{\,\mathrm{Aut}\,}}(G)$ the set $\sigma ^{-1} I$ is independent, we get

$$\begin{aligned} \begin{aligned} \sum _{y \in U} A(x_0, y)&= \sum _{y \in U} \omega (I)^{-1} \int _\Gamma \chi _I(\sigma x_0) \chi _I(\sigma y)\, d\mu (\sigma )\\&=\omega (I)^{-1} \int _\Gamma \chi _I(\sigma x_0) \sum _{y \in U} \chi _I(\sigma y)\, d\mu (\sigma )\\&=\omega (I)^{-1}\int _\Gamma \chi _I(\sigma x_0) |U \cap \sigma ^{-1}I|\, d\mu (\sigma )\\&\le \frac{\alpha (G[U])}{\omega (V)}=\alpha (G[U]) A(x_0, x_0). \end{aligned} \end{aligned}$$

Notice these constraints do not come directly from ${\mathcal {C}}(V)$ or ${{\,\mathrm{BQC}\,}}(V)$, since they rely on the edge set of the graph. Theorem 5.1 says that they must be somehow implied by the constraints coming from ${\mathcal {C}}(V)$ together with the other constraints of problem $\vartheta (G, {\mathcal {C}}(V))$, but the way in which this implication is carried out is not necessarily simple: it could be that only by adding many constraints from the completely positive cone for sets other than *U* one would get the implication.

The situation is clearer when one considers instead the Boolean-quadratic cone. In this case, a subgraph constraint for a given finite $U \subseteq V$ and a given $x_0 \in V$ is implied by a single constraint from ${{\,\mathrm{BQC}\,}}(U \cup \{x_0\})$ together with the constraints $A(x, y) = 0$ for adjacent *x* and *y*.

To see this, assume for the sake of simplicity that $x_0 \notin U$ and write $U' = U \cup \{x_0\}$ (if $x_0 \in U$, a simple modification of the argument below works). Let $C:U' \times U' \rightarrow {\mathbb {R}}$ be the matrix such that

$$\begin{aligned} C(x, y) = {\left\{ \begin{array}{ll} \alpha (G[U])&{}\hbox { if }\; x = y = x_0;\\ -1/2&{} \hbox { if }\; x = x_0\; \hbox {or}\; y = x_0;\\ 0&{}\text {otherwise.} \end{array}\right. } \end{aligned}$$

Then the subgraph constraint (25) is

$$\begin{aligned} \sum _{x,y \in U'} C(x, y) A(x, y) \ge 0. \end{aligned}$$

We now show that there are matrices $Z \in {{\,\mathrm{BQC}\,}}^*(U')$ and $B:U' \times U' \rightarrow {\mathbb {R}}$ such that $B(x, y) = 0$ if *x*, $y \in U$ are not adjacent satisfying $C = Z + B$, and it will follow that, if *A* is feasible for $\vartheta (G, {{\,\mathrm{PSD}\,}}(V))$ and $\sum _{x,y \in U'} Z(x,y) A(x,y) \ge 0$, then

$$\begin{aligned} \sum _{x,y \in U'} C(x, y) A(x, y) = \sum _{x,y \in U'} Z(x, y) A(x, y) + \sum _{x,y \in U'} B(x, y) A(x, y) \ge 0, \end{aligned}$$

whence *A* satisfies the subgraph constraint.

For *Z*, consider the matrix

26 $$\begin{aligned} Z(x, y) = {\left\{ \begin{array}{ll} \alpha (G[U])&{}\text {if }x = y = x_0;\\ -1/2&{}\text {if }x = x_0 \text { or}~y = x_0;\\ 1/2&{}\text {if }(x, y) \in E;\\ 0&{}\text {otherwise}, \end{array}\right. } \end{aligned}$$

and for *B* take the matrix with $-1/2$ on entries corresponding to edges of *G*[*U*] and 0 everywhere else. Then $C = Z + B$, and it remains to show that $Z \in {{\,\mathrm{BQC}\,}}^*(U')$. To this end, take $f:U' \rightarrow \{0,1\}$. If $f(x_0) = 0$, then clearly $\langle Z, f \otimes f^*\rangle \ge 0$. So suppose $f(x_0) = 1$ and write $S = \{\, x \in U : f(x) = 1\,\}$. Then

$$\begin{aligned} \langle Z, f \otimes f^*\rangle = \alpha (G[U]) - |S| + |E(G[S])|. \end{aligned}$$

Now let $X \subseteq S$ be a maximal independent set in *G*[*S*]. Then $|X| \le \alpha (G[U])$. Since *X* is maximal, every $y \in S {\setminus } X$ is adjacent to some $x \in X$, so $|S{\setminus } X| \le |E(G[S])|$, and

$$\begin{aligned} \alpha (G[U]) - |S| + |E(G[S])| = \alpha (G[U]) - |X| - |S{\setminus } X| + |E(G[S])| \ge 0, \end{aligned}$$

showing that $$Z \in {{\,\mathrm{BQC}\,}}^*(U')$$.

Finally, subgraph constraints can also be used for distance graphs on ${\mathbb {R}}^n$: given a set $D \subseteq (0, \infty )$ of forbidden distances, one can add to $\vartheta (G({\mathbb {R}}^n, D), {{\,\mathrm{PSD}\,}}({\mathbb {R}}^n))$ any number of constraints of the form

$$\begin{aligned} \sum _{y \in U} f(x_0 - y) \le \alpha (G({\mathbb {R}}^n, D)[U]) f(0), \end{aligned}$$

where $U \subseteq {\mathbb {R}}^n$ is finite and $x_0 \in {\mathbb {R}}^n$ is fixed. Such constraints have been used by Oliveira and Vallentin [36] to get improved upper bounds for the independence density of the unit-distance graph on ${\mathbb {R}}^n$ in several dimensions; the sets *U* used were always vertex sets of regular simplices in ${\mathbb {R}}^n$. Keleti et al. [22] used the points of the Moser spindle to get improved bounds for the independence density of $G({\mathbb {R}}^2, \{1\})$; Bachoc et al. [2] used several different graphs to get better bounds for the independence density of $G({\mathbb {R}}^n, \{1\})$ for $n = 4$, ..., 24 and a better asymptotic bound.

#### A new class of graphical facets of the Boolean-quadratic cone

The matrix *Z* defined in (26) is sometimes an extreme ray of ${{\,\mathrm{BQC}\,}}^*(U')$, that is, $\langle Z, A\rangle \ge 0$ induces a facet of ${{\,\mathrm{BQC}\,}}(U')$. In fact, matrices like *Z* comprise a whole class of facets of the Boolean-quadratic cone that generalizes the class of clique inequalities introduced by Padberg [37].

Let $G = (V, E)$ be a finite graph with at least two vertices. We say that *G* is $\alpha$ *-critical* if $\alpha (G - e) > \alpha (G)$ for all $e \in E$; $\alpha$-critical graphs have been extensively studied in the context of combinatorial optimization [42, §68.5].

Assume $\emptyset \notin V$ and write $W = V \cup \{\emptyset \}$. Consider the matrix $Q_G:W \times W \rightarrow {\mathbb {R}}$ defined as

$$\begin{aligned} Q_G(x, y) = {\left\{ \begin{array}{ll} \alpha (G)&{}\text {if }x = y = \emptyset ;\\ -1/2&{}\text {if }x = \emptyset \textit{ or}~y = \emptyset ;\\ 1/2&{}\text {if }(x, y) \in E;\\ 0&{}\text {otherwise}. \end{array}\right. } \end{aligned}$$

##### Theorem 7.4

Let $G = (V, E)$ be a finite graph with at least two vertices, and assume $\emptyset \notin V$. The inequality $\langle Q_G, A\rangle \ge 0$ induces a facet of ${{\,\mathrm{BQC}\,}}(W)$, where $W = V \cup \{\emptyset \}$, if and only if *G* is connected and $\alpha$-critical.

##### Proof

The argument given in the previous section shows that $\langle Q_G, A\rangle \ge 0$ is valid for ${{\,\mathrm{BQC}\,}}(W)$; let us then establish the necessary and sufficient conditions for it to be facet defining.

As a subset of the space of symmetric matrices indexed by $W \times W$, the cone ${{\,\mathrm{BQC}\,}}(W)$ is full dimensional. Indeed, it suffices to notice that the $1+|W|(|W|+1)/2$ matrices $\chi _U \otimes \chi _U^*$ for $U \subseteq W$ with $|U| \le 2$ are affinely independent.

We first show necessity. If $G = G_1 + G_2$, where $G_1$, $G_2$ have disjoint vertex sets and $G_1$ is a connected component of *G*, then $Q_G = Q_{G'_1} + P$, where $G'_1 = (V, E(G_1))$ and $P:W \times W \rightarrow {\mathbb {R}}$ is such that $P(\emptyset , \emptyset ) = \alpha (G_2)$ and $P(x, y) = 1/2$ if $(x, y) \in E(G_2)$. Now $\langle Q_{G'_1}, A\rangle \ge 0$ is valid for ${{\,\mathrm{BQC}\,}}(W)$ and, since $P \ge 0$, so is $\langle P, A\rangle \ge 0$. Since $\alpha (G) = \alpha (G_1) + \alpha (G_2)$ and since ${{\,\mathrm{BQC}\,}}(W)$ is full dimensional, we see that $\langle Q_G, A\rangle \ge 0$ does not induce a facet.

Similarly, if $\alpha (G - e) = \alpha (G)$ for some $e = (x, y) \in E$, then $Q_G = Q_{G - e} + P$, where $P(x, y) = P(y, x) = 1/2$, and we see that $\langle Q_G, A\rangle \ge 0$ does not induce a facet.

To see sufficiency, assume *G* is connected and $\alpha$-critical. Now suppose $Z:W \times W \rightarrow {\mathbb {R}}$ is such that $\langle Z, A\rangle \ge 0$ induces a facet of ${{\,\mathrm{BQC}\,}}(W)$ and

$$\begin{aligned} \{\, A \in {{\,\mathrm{BQC}\,}}(W) : \langle Q_G, A\rangle = 0\,\} \subseteq \{\, A \in {{\,\mathrm{BQC}\,}}(W) : \langle Z, A\rangle = 0\,\}. \end{aligned}$$

To show that $\langle Q_G, A\rangle \ge 0$ induces a facet it suffices to show that *Z* is a nonnegative multiple of $Q_G$.

To this end, notice first that if $x \in V$, then $\langle Q_G, \chi _{\{x\}} \otimes \chi _{\{x\}}^*\rangle = 0$, so

$$\begin{aligned} Z(x, x) = \langle Z, \chi _{\{x\}} \otimes \chi _{\{x\}}^*\rangle = 0. \end{aligned}$$

Next, let *x*, $y \in V$ and assume $(x, y) \notin E$. Then $\langle Q_G, \chi _{\{x,y\}} \otimes \chi _{\{x,y\}}^* \rangle = 0$, whence

$$\begin{aligned} Z(x, y) = Z(y, x) = \langle Z, \chi _{\{x,y\}} \otimes \chi _{\{x,y\}}^* \rangle = 0. \end{aligned}$$

Note that, for all $U \subseteq V$, if $S = U \cup \{\emptyset \}$, then

$$\begin{aligned} \langle Q_G, \chi _S \otimes \chi _S^*\rangle = \alpha (G) - |U| + |E(G[U])|. \end{aligned}$$

Take now $(x, y) \in E$. Let $I \subseteq V$ be a maximum independent set in $G - (x, y)$; then $|I| = \alpha (G) + 1$ and hence we must have *x*, $y \in I$. Write $S = I \cup \{\emptyset \}$, so

$$\begin{aligned} \langle Q_G, \chi _S \otimes \chi _S^*\rangle = \alpha (G) - (\alpha (G) + 1) + 1 = 0 \end{aligned}$$

and similarly

$$\begin{aligned} \langle Q_G, \chi _{S-x} \otimes \chi _{S-x}^*\rangle = 0, \end{aligned}$$

whence $\langle Z, \chi _S \otimes \chi _S^*\rangle = \langle Z, \chi _{S-x} \otimes \chi _{S-x}^*\rangle = 0$. Now, since $Z(x, y) = 0$ if $(x, y) \notin E$,

$$\begin{aligned} \begin{aligned} 0&= \langle Z, \chi _S \otimes \chi _S^*\rangle \\&= \langle Z, \chi _{S-x} \otimes \chi _{S-x}^*\rangle + 2 Z(\emptyset , x) + 2 Z(x, y)\\&= 2 Z(\emptyset , x) + 2 Z(x, y). \end{aligned} \end{aligned}$$

Since *x* and *y* are interchangeable in the above argument, we see immediately that $Z(\emptyset , x) = -Z(x, y) = Z(\emptyset , y)$. Now *G* is connected, and so it follows immediately that there is a number *a* such that $Z(\emptyset , x) = -a$ for all $x \in V$ and $Z(x, y) = a$ for all $(x, y) \in E$.

We are almost done. If $(x, y) \in E$, then $\langle Z, \chi _{\{x,y\}} \otimes \chi _{\{x,y\}}^*\rangle \ge 0$, so $a \ge 0$. If *I* is a maximum independent set in *G* and $S = I \cup \{\emptyset \}$, then $\langle Q_G, \chi _S \otimes \chi _S^*\rangle = 0$ and

$$\begin{aligned} 0 = \langle Z, \chi _S \otimes \chi _S^*\rangle = Z(\emptyset , \emptyset ) - 2a|I|, \end{aligned}$$

whence $Z(\emptyset , \emptyset ) = 2a\alpha (G)$ and $Z = 2aQ_G$, as we wanted. $\square$

### An alternative normalization and polytope constraints

The constraint “${{\,\mathrm{tr}\,}}A = 1$” in (6) is there to prevent the problem from being unbounded: it is a *normalization constraint*. There is another kind of normalization constraint that can be used to replace the trace constraint; by doing so we obtain an equivalent problem and also gain the ability to add to our problem constraints from the *Boolean-quadratic polytope*, which given a nonempty finite set *V* is defined as

$$\begin{aligned} {{\,\mathrm{BQP}\,}}(V) = {{\,\mathrm{conv}\,}}\{\, f \otimes f^* : f:V\rightarrow \{0,1\}\,\}. \end{aligned}$$

Such constraints are also implied by constraints from the Boolean-quadratic cone, but in practice, given our limited computational power, they are useful. For instance, the inclusion–exclusion inequalities used by Keleti et al. [22] to get better upper bounds for $G({\mathbb {R}}^2, \{1\})$ come from facets of ${{\,\mathrm{BQP}\,}}(V)$, as we will soon see.

Let $G = (V, E)$ be a topological graph where *V* is a compact Hausdorff space, $\omega$ be a finite Borel measure on *V*, and ${\mathcal {K}}(V) \subseteq {{\,\mathrm{PSD}\,}}(V)$ be a convex cone. Since ${\mathcal {K}}(V)$ is a subset of the cone of positive kernels, Mercer’s theorem implies that any continuous kernel in ${\mathcal {K}}(V)$ is trace class and that the trace is the integral over the diagonal. The alternative version of (6) is:

27 $$\begin{aligned} \begin{array}{r@{\ }l@{\quad }l} \text {maximize}&{}{{\,\mathrm{tr}\,}}A\\ &{}A(x, y) = 0\quad \hbox { if}~\ (x, y) \in E,\\ &{}\begin{pmatrix} 1&{}\ {{\,\mathrm{tr}\,}}A\\ {{\,\mathrm{tr}\,}}A&{}\ \langle J, A\rangle \end{pmatrix}\text { is positive semidefinite,}\\ &{}A \text {is continuous and}~A \in {\mathcal {K}}(V). \end{array} \end{aligned}$$

If *A* is a feasible solution of the above problem, then $A' = ({{\,\mathrm{tr}\,}}A)^{-1} A$ is feasible for $\vartheta (G, {\mathcal {K}}(V))$. Moreover, the positive-semidefiniteness of the $2 \times 2$ matrix in (27) implies that $({{\,\mathrm{tr}\,}}A)^2 \le \langle J, A\rangle$, whence

$$\begin{aligned} \langle J, A'\rangle = ({{\,\mathrm{tr}\,}}A)^{-1} \langle J, A\rangle \ge {{\,\mathrm{tr}\,}}A, \end{aligned}$$

so $\vartheta (G, {\mathcal {K}}(V))$ is $\ge$ the optimal value of (27). The reverse inequality is also true: if *A* is a feasible solution of (6), then one easily checks that $A' = \langle J, A\rangle A$ is a feasible solution of (27) and that ${{\,\mathrm{tr}\,}}A' = \langle J, A\rangle$. So problems (6) and (27) are actually equivalent.

Fix a finite set $U \subseteq V$ and let $Z:U \times U \rightarrow {\mathbb {R}}$ be a symmetric matrix and $\beta$ be a real number such that $\langle Z, A\rangle \ge \beta$ is a valid inequality for ${{\,\mathrm{BQP}\,}}(U)$, that is, $\langle Z, A\rangle \ge \beta$ for all $A \in {{\,\mathrm{BQP}\,}}(U)$.

If *G* and $\omega$ satisfy the hypotheses of Theorem 5.1, then any number of constraints

28 $$\begin{aligned} \sum _{x,y \in U} Z(x, y) A(x, y) \ge \beta \end{aligned}$$

can be added to (27) with ${\mathcal {K}}(V) = {{\,\mathrm{PSD}\,}}(V)$ and we still get an upper bound for $\alpha _\omega (G)$. Indeed, if *I* is a measurable independent set of positive measure, then $A = R(\chi _I \otimes \chi _I^*)$ is easily checked to be a feasible solution of (27) with ${\mathcal {K}}(V) = {{\,\mathrm{PSD}\,}}(V)$ that moreover satisfies (28), and ${{\,\mathrm{tr}\,}}A = \omega (I)$. The alternative normalization is essential for this approach to work: if we try to add constraint (28) to (6), then if $\beta \ne 0$ we get a *nonlinear* constraint because of the different normalization, making it more difficult to deal with the resulting problem in practice.

The same ideas can be applied to problem (21). First, given a closed set $D \subseteq (0, \infty )$ of forbidden distances, we consider an alternative normalization that gives rise to an equivalent problem:

29 $$\begin{aligned} \begin{array}{r@{\ }l@{\quad }l} \text {maximize}&{}f(0)\\ &{}f(x) = 0\quad \hbox { if}~\ \Vert x\Vert \in D,\\ &{}\begin{pmatrix} 1&{}\ f(0)\\ f(0)&{}\ M(f) \end{pmatrix}\text { is positive semidefinite,}\\ &{}{f:{\mathbb {R}}^n \rightarrow {\mathbb {R}}\text { is continuous and}~f \in {\mathcal {K}}({\mathbb {R}}^n).} \end{array} \end{aligned}$$

Then, we observe that we can add to this problem, with ${\mathcal {K}}({\mathbb {R}}^n) = {{\,\mathrm{PSD}\,}}({\mathbb {R}}^n)$, any number of constraints of the form

30 $$\begin{aligned} \sum _{x, y \in U} Z(x, y) f(x - y) \ge \beta \end{aligned}$$

for finite $U \subseteq {\mathbb {R}}^n$ and *Z*, $\beta$ such that $\langle Z, A\rangle \ge \beta$ is valid for ${{\,\mathrm{BQP}\,}}(U)$ and still prove that the optimal value provides an upper bound for the independence density of $G({\mathbb {R}}^n, D)$.

Given points $x_1$, ..., $x_N \in {\mathbb {R}}^n$, the inclusion-exclusion inequality used by Keleti, Matolcsi, Oliveira, and Ruzsa is

$$\begin{aligned} \sum _{1 \le i < j \le N} f(x_i - x_j) - N f(0) \ge -1. \end{aligned}$$

This constraint is just (30) with *Z* such that $Z(x_i, x_i) = -1$ for all *i* and $Z(x_i, x_j) = 1/2$ for all $i \ne j$. It can be easily checked that $\langle Z, A\rangle \ge -1$ is a valid inequality for ${{\,\mathrm{BQP}\,}}(\{x_1, \ldots , x_N\})$; one can even verify that it gives a facet of the polytope, simply by finding enough affinely independent points in the polytope for which the inequality is tight.

Constraints from ${{\,\mathrm{BQP}\,}}(U)$ for a finite $U \subseteq {\mathbb {R}}^n$ are implied by constraints from ${{\,\mathrm{BQC}\,}}(U \cup \{\emptyset \})$ together with the other constraints from (6) or (21). It is still useful to consider constraints from ${{\,\mathrm{BQP}\,}}(U)$ mainly since $U \cup \{\emptyset \}$ is a larger set than *U*, and therefore computing the facets of ${{\,\mathrm{BQC}\,}}(U \cup \{\emptyset \})$ can be much harder than computing the facets of ${{\,\mathrm{BQC}\,}}(U)$, as is the case already when $|U|=6$. For instance, Deza and Laurent [11, §30.6] survey some numbers for the cut polytope, which is equivalent to the Boolean-quadratic polytope under a linear transformation. For 6 points, the total number of facets is 116, 764, distributed among 11 equivalence classes. The approach we use to find violated constraints cannot, however, exploit the full symmetry of the polytope, so we end up using a list of 428 facets. For 7 points, the total number of facets is 217, 093, 472, distributed among 147 classes. Taking into account the smaller symmetry group we use, the total list of facets needed for our procedure would have more than ten thousand entries.

## Better upper bounds for the independence number of graphs on the sphere

By adding ${{\,\mathrm{BQP}\,}}(U)$-constraints to $\vartheta (G(S^{n-1}, \{\pi /2\}), {{\,\mathrm{PSD}\,}}(S^{n-1}))$ using the approach described in Sect. 7.2, one is able to improve on the best upper bounds for $\alpha _\omega (G(S^{n-1}, \{\pi /2\})) = m_0(S^{n-1})$. Table 1 shows bounds thus obtained for the *independence ratio*, namely

$$\begin{aligned} \alpha _\omega (G(S^{n-1}, \{\pi /2\})) / \omega _n, \end{aligned}$$

for $n = 3$, ..., 8. The rest of this section is devoted to an explanation of how these bounds were computed. The bounds have also been checked to be correct; the verification procedure is explained in detail in a document available with the arXiv version of this paper. The programs used for verification can also be found with the arXiv version.

### Invariant kernels on the sphere

Let $\mathrm {O}(n)$ be the orthogonal group on ${\mathbb {R}}^n$, that is, the group of $n \times n$ orthogonal matrices. The orthogonal group acts on a kernel $A:S^{n-1} \times S^{n-1} \rightarrow {\mathbb {R}}$ by

$$\begin{aligned} (T \cdot A)(x, y) = A(T^{-1}x, T^{-1}y), \end{aligned}$$

where $T \in \mathrm {O}(n)$; we say that *A* is *invariant* if $T\cdot A = A$ for all $T \in \mathrm {O}(n)$. An invariant kernel is thus a real-valued function with domain $[-1,1]$, since if $x \cdot y = x' \cdot y'$, then $A(x', y') = A(x, y)$.

Let $D \subseteq (0, \pi ]$ be a set of forbidden distances. If the cone ${\mathcal {K}}(S^{n-1})$ is invariant under the action of the orthogonal group, then one can add to the problem $\vartheta (G(S^{n-1}, D), {\mathcal {K}}(S^{n-1}))$ the restriction that *A* has to be invariant without changing the optimal value of the resulting problem. Indeed, if *A* is a feasible solution, then so is $T \cdot A$ for all $T \in \mathrm {O}(n)$, and hence its *symmetrization*

$$\begin{aligned} {\overline{A}}(x, y) = \int _{\mathrm {O}(n)} A(T^{-1}x, T^{-1}y)\, d\mu (T), \end{aligned}$$

where $\mu$ is the Haar measure on $\mathrm {O}(n)$, is also feasible and has the same objective value as *A*.

The advantage of requiring *A* to be invariant is that invariant and positive kernels can be easily parameterized. Indeed, let $P_k^n$ denote the Jacobi polynomial of degree *k* and parameters $(\alpha , \alpha )$, where $\alpha = (n - 3) / 2$, normalized so $P_k^n(1) = 1$ (for background on Jacobi polynomials, see the book by Szegö [44]). A theorem of Schoenberg [40] says that $A:S^{n-1} \times S^{n-1} \rightarrow {\mathbb {R}}$ is continuous, invariant, and positive if and only if there are nonnegative numbers *a*(0), *a*(1), ... such that $\sum _{k=0}^\infty a(k) < \infty$ and

31 $$\begin{aligned} A(x, y) = \sum _{k=0}^\infty a(k) P_k^n(x \cdot y) \end{aligned}$$

for all *x*, $y \in S^{n-1}$; in particular, the sum above converges absolutely and uniformly on $S^{n-1} \times S^{n-1}$.

### Primal and dual formulations

When a continuous, invariant, and positive kernel *A* is represented as in (31), constraint (28) becomes

$$\begin{aligned} \beta \le \sum _{x,y \in U} Z(x, y) A(x, y) = \sum _{k=0}^\infty a(k) \sum _{x,y \in U} Z(x, y) P_k^n(x \cdot y) = \sum _{k=0}^\infty a(k) r(k), \end{aligned}$$

where $r:{\mathbb {N}}\rightarrow {\mathbb {R}}$ is the function such that

$$\begin{aligned} r(k) = \sum _{x, y \in U} Z(x, y) P_k^n(x \cdot y). \end{aligned}$$

Let ${\mathcal {R}}$ be a finite collection of ${{\,\mathrm{BQP}\,}}(U)$-constraints represented as pairs $(r, \beta )$, where *r* is given by the above expression for a valid inequality $\langle Z, A\rangle \ge \beta$ for ${{\,\mathrm{BQP}\,}}(U)$ for some finite $U \subseteq S^{n-1}$.

If a continuous, invariant, and positive kernel *A* is given by expression (31), then $\langle J, A\rangle = \omega _n^2 a(0)$. Moreover, all diagonal entries of *A* are the same, and hence

$$\begin{aligned} {{\,\mathrm{tr}\,}}A = \omega _n \sum _{k=0}^\infty a(k). \end{aligned}$$

Using the alternative normalization of Sect. 7.2, problem $\vartheta (G(S^{n-1}, \{\theta \}), {{\,\mathrm{PSD}\,}}(S^{n-1}))$, strengthened with the ${{\,\mathrm{BQP}\,}}(U)$-constraints in ${\mathcal {R}}$, can be equivalently written as

32 $$\begin{aligned} \begin{array}{r@{\ }l@{\quad }l} \text {maximize}&{}\sum _{k=0}^\infty a(k)\\ &{}\sum _{k=0}^\infty a(k) P_k^n(\cos \theta ) = 0,\\ &{}\sum _{k=0}^\infty a(k) r(k) \ge \beta \quad \text {for }(r, \beta ) \in {\mathcal {R}},\\ &{}\begin{pmatrix} 1&{}\ \omega _n\sum _{k=0}^\infty a(k)\\ \omega _n\sum _{k=0}^\infty a(k)&{}\omega _n^2 a(0) \end{pmatrix}\text { is positive semidefinite,}\\ &{}a(k) \ge 0 \text { for all}~k \ge 0. \end{array} \end{aligned}$$

Notice that the objective function was scaled so the optimal value is a bound for the independence ratio $\alpha _\omega (G(S^{n-1}, \{\theta \})) / \omega _n$.

A dual for this problem is the following optimization problem on variables $\lambda$, $y(r, \beta )$ for $(r, \beta ) \in {\mathcal {R}}$, and $z_1$, $z_2$, $z_3$:

33 $$\begin{aligned} \begin{array}{r@{\ }l@{\quad }l} \text {minimize}&{}z_1 + \sum _{(r, \beta ) \in {\mathcal {R}}} y(r, \beta ) \beta \\ &{}\lambda + \sum _{(r, \beta ) \in {\mathcal {R}}} y(r, \beta ) r(0) + z_2\omega _n + z_3\omega _n^2 \ge 1,\\ &{}\lambda P_k^n(\cos \theta ) + \sum _{(r, \beta ) \in {\mathcal {R}}} y(r, \beta ) r(k) + z_2\omega _n \ge 1,\quad \text {for } k \ge 1,\\ &{}\begin{pmatrix} z_1&{}-\frac{1}{2} z_2\\ -\frac{1}{2}z_2&{}-z_3 \end{pmatrix}\text { is positive semidefinite,}\\ &{}y \le 0. \end{array} \end{aligned}$$

In practice, this is the problem that we solve to obtain an upper bound; there are two main reasons for this. The first one comes from weak duality: the objective value of any feasible solution of this problem is an upper bound for the independence ratio. Indeed, let $\lambda$, *y*, $z_1$, $z_2$, $z_3$ be a feasible solution of (33) and *a* be a feasible solution of (32). Then

$$\begin{aligned} \begin{aligned} z_1 + \sum _{(r, \beta ) \in {\mathcal {R}}} y(r, \beta ) \beta&\ge z_1 + \sum _{(r, \beta ) \in {\mathcal {R}}} y(r, \beta ) \sum _{k=0}^\infty a(k) r(k)\\&= z_1 + \sum _{k=0}^\infty a(k) \sum _{(r, \beta ) \in {\mathcal {R}}} y(r, \beta ) r(k)\\&\ge z_1 + a(0)(-z_3\omega _n^2) + \sum _{k=0}^\infty a(k) (1 - \lambda P_k^n(\cos \theta ) - z_2 \omega _n)\\&=z_1 - z_3\omega _n^2 a(0) + (1-z_2\omega _n) \sum _{k=0}^\infty a(k) - \lambda \sum _{k=0}^\infty a(k) P_k^n(\cos \theta )\\&=z_1 - z_3\omega _n^2 a(0) - z_2\omega _n \sum _{k=0}^\infty a(k) + \sum _{k=0}^\infty a(k)\\&\ge \sum _{k=0}^\infty a(k), \end{aligned} \end{aligned}$$

as we wanted, where for the last inequality we use the positive-semidefiniteness of the $2 \times 2$ matrices in (32) and (33).

The second reason is that the dual is a semidefinite program with finitely many variables, though infinitely many constraints, including one constraint for each $k \ge 0$. In practice, we choose $d > 0$ and disregard all constraints for $k > d$. Then we solve a finite semidefinite program, and later on we prove that a suitable modification of the solution found is indeed feasible for the infinite problem, as we will see now.

### Finding feasible dual solutions and checking them

To find good feasible solutions of (33), we start by taking ${\mathcal {R}}= \emptyset$. Then we turn our problem into a finite one: we choose $d > 0$ and disregard all constraints for $k > d$. We have then a finite semidefinite program, which we solve using standard semidefinite programming solvers. The idea is that, if *d* is large enough, then the solution found will be close enough to being feasible, and so by slightly changing $z_1$, $z_2$, and $z_3$ we will be able to find a feasible solution.

By solving the finite problem we obtain at the same time an optimal solution of the corresponding finite primal problem, in which $a(k) = 0$ if $k > d$ (notice this is likely not an optimal solution of the original primal problem). We use this primal solution to perform a *separation round*, that is, to look for violated polytope constraints that we can add to the problem. One way to do this is as follows.

Say *a* is the primal solution and let

$$\begin{aligned} A(x, y) = \sum _{k=0}^\infty a(k) P_k^n(x \cdot y). \end{aligned}$$

Fix an integer $N \ge 2$, write $[N] = \{1, \ldots , N\}$, and let $Z \in {\mathbb {R}}^{N \times N}$, $\beta \in {\mathbb {R}}$ be such that $\langle Z, X \rangle \ge \beta$ is valid for ${{\,\mathrm{BQP}\,}}([N])$. Then we try to find points $x_1$, ..., $x_N \in S^{n-1}$ that maximize the violation

34 $$\begin{aligned} \beta - \sum _{i,j=1}^N Z(i, j) A(x_i, x_j) \end{aligned}$$

of the polytope inequality. If we find points such that the violation is positive, then we have a violated constraint which can be added to ${\mathcal {R}}$; the whole procedure can then be repeated: the dual problem is solved again and a new separation round is performed.

To find violated constraints we need to know valid inequalities, or better yet facets, of ${{\,\mathrm{BQP}\,}}([N])$. Up to $N = 6$ it is possible to work with a full list of facets; for $N = 7$ only with a partial list. To find points $x_1$, ..., $x_N \in S^{n-1}$ maximizing (34), we represent the points on the sphere by stereographic projection on the $x_n = -1$ plane and use some method for unconstrained optimization that converges to a local optimum.

After a few optimization/separation rounds, one starts to notice only minor improvements to the bound. Then it is time to check how far from feasible the dual solution is and to fix it in order to get a truly feasible solution and therefore an upper bound. A detailed description of the verification procedure, together with a program to check the dual solutions used for the results in this section, can be found together with the arXiv version of this paper.

## Better upper bounds for the independence density of unit-distance graphs

Just like in the case of graphs on the sphere, we can add ${{\,\mathrm{BQP}\,}}(U)$-constraints to $\vartheta (G({\mathbb {R}}^n, \{1\}), {{\,\mathrm{PSD}\,}}({\mathbb {R}}^n))$ and so obtain improved upper bounds for $\alpha _{{\bar{\delta }}}(G({\mathbb {R}}^n , \{1\}))$ for $n = 3$, ..., 8. These improved upper bounds then provide new lower bounds for the *measurable chromatic number* $\chi _{\mathrm {m}}(G({\mathbb {R}}^n, \{1\}))$ of the unit-distance graph, which is the minimum number of measurable independent sets needed to partition ${\mathbb {R}}^n$, for $n = 4$, ..., 8. Indeed, since

$$\begin{aligned} \alpha _{{\bar{\delta }}}(G({\mathbb {R}}^n, \{1\})) \chi _{\mathrm {m}}(G({\mathbb {R}}^n, \{1\})) \ge 1, \end{aligned}$$

if $$\alpha _{{\bar{\delta }}}(G({\mathbb {R}}^n, \{1\})) \le u$$, then $$\chi _{\mathrm {m}}(G({\mathbb {R}}^n, \{1\})) \ge \lceil 1/u \rceil $$.

Table 2 shows these new bounds compared to the previously best ones. To obtain the bounds for $n = 4$, ..., 8, subgraph constraints (see Sect. 7.1) have also been used. In the remainder of this section we will see how these bounds have been computed; they have also been checked to be correct, and the verification procedure is explained in detail in a document available with the arXiv version of this paper. The programs used for the verification can also be found with the arXiv version.

### Radial functions

The orthogonal group $\mathrm {O}(n)$ acts on a function $f:{\mathbb {R}}^n \rightarrow {\mathbb {C}}$ by

$$\begin{aligned} (T \cdot f)(x) = f(T^{-1}x), \end{aligned}$$

where $T \in \mathrm {O}(n)$; we say that *f* is *radial* if it is invariant under this action, that is, if $T \cdot f = f$ for all $T \in \mathrm {O}(n)$. A radial function *f* is thus a function of one real variable, since if $\Vert x\Vert = \Vert y\Vert$, then $f(x) = f(y)$.

Let $D \subseteq (0, \infty )$ be a set of forbidden distances. If the cone ${\mathcal {K}}({\mathbb {R}}^n) \subseteq L^\infty ({\mathbb {R}}^n)$ is invariant under the action of the orthogonal group, then one can add to the problem $\vartheta (G({\mathbb {R}}^n, D), {\mathcal {K}}({\mathbb {R}}^n))$ the restriction that *f* has to be radial without changing the optimal value of the resulting problem. Indeed, if *f* is a feasible solution, then so is $T \cdot f$ for all $T \in \mathrm {O}(n)$, and hence its *radialization*

$$\begin{aligned} {\overline{f}}(x) = \int _{\mathrm {O}(n)} f(T^{-1} x)\, d\mu (T) = \frac{1}{\omega (S^{n-1})} \int _{S^{n-1}} f(\Vert x\Vert \xi )\, d\omega (\xi ), \end{aligned}$$

where $\mu$ is the Haar measure on $\mathrm {O}(n)$, is also feasible and has the same objective value as *f*.

The advantage of requiring *f* to be radial is that radial functions of positive type can be easily parameterized. Indeed, if $f \in {{\,\mathrm{PSD}\,}}({\mathbb {R}}^n)$ is continuous, then Bochner’s theorem says that there is a finite Borel measure $\nu$ on ${\mathbb {R}}^n$ such that

$$\begin{aligned} f(x) = \int _{{\mathbb {R}}^n} e^{i u \cdot x}\, d\nu (u). \end{aligned}$$

But then we obtain the following expression, due to Schoenberg [39], for the radialization of *f*:

35 $$\begin{aligned} \begin{aligned} {\overline{f}}(x)&= \frac{1}{\omega (S^{n-1})}\int _{S^{n-1}} \int _{{\mathbb {R}}^n} e^{i u \cdot \Vert x\Vert \xi }\, d\nu (u) d\omega (\xi )\\&=\int _{{\mathbb {R}}^n} \frac{1}{\omega (S^{n-1})} \int _{S^{n-1}} e^{i u \cdot \Vert x\Vert \xi }\, d\omega (\xi ) d\nu (u)\\&= \int _0^\infty \Omega _n(t \Vert x\Vert )\, d\alpha (t), \end{aligned} \end{aligned}$$

where

36 $$\begin{aligned} \Omega _n(\Vert u\Vert ) = \frac{1}{\omega (S^{n-1})} \int _{S^{n-1}} e^{i u \cdot \xi }\, d\omega (\xi ) \end{aligned}$$

for $u \in {\mathbb {R}}^n$ and $\alpha$ is the Borel measure on $[0, \infty )$ such that

$$\begin{aligned} \alpha (X) = \nu (\{\, \lambda \xi : \lambda \in X \text { and } \xi \in S^{n-1}\,\}) \end{aligned}$$

for every measurable set *X*. The function $\Omega _n$ has a simple expression in terms of Bessel functions, namely

37 $$\begin{aligned} \Omega _n(t) = \Gamma \Bigl (\frac{n}{2}\Bigr ) \Bigl (\frac{2}{t}\Bigr )^{(n-2) / 2} J_{(n-2)/2}(t) \end{aligned}$$

for $t > 0$ and $\Omega _n(0) = 1$, where $J_\alpha$ denotes the Bessel function of first kind of order $\alpha$ (for background, see the book by Watson [47]).

### Primal and dual formulations

When a continuous radial function *f* of positive type is represented as in (35), constraint (30) becomes

$$\begin{aligned} \beta \le \sum _{x,y \in U} Z(x, y) f(x-y) = \int _0^\infty \sum _{x,y \in U} Z(x, y) \Omega _n(t \Vert x-y\Vert )\, d\alpha (t) = \int _0^\infty r(t)\, d\alpha (t), \end{aligned}$$

where $r:[0, \infty ) \rightarrow {\mathbb {R}}$ is the continuous function such that

$$\begin{aligned} r(t) = \sum _{x,y \in U} Z(x, y) \Omega _n(t\Vert x-y\Vert ). \end{aligned}$$

As shown in Sect. 7.1, a subgraph constraint is implied by one ${{\,\mathrm{BQP}\,}}(U)$-constraint together with the other constraints of $\vartheta (G({\mathbb {R}}^n, \{1\}), {{\,\mathrm{PSD}\,}}({\mathbb {R}}^n))$, so in the discussion below we treat them as ${{\,\mathrm{BQP}\,}}(U)$-constraints.

Let ${\mathcal {R}}$ be a finite collection of ${{\,\mathrm{BQP}\,}}(U)$-constraints represented as pairs $(r, \beta )$, where *r* is given by the above expression for a valid inequality $\langle Z, A\rangle \ge \beta$ for ${{\,\mathrm{BQP}\,}}(U)$ for some finite $U \subseteq {\mathbb {R}}^n$. Using the alternative normalization of Sect. 7.2, problem $\vartheta (G({\mathbb {R}}^n, \{1\}), {{\,\mathrm{PSD}\,}}({\mathbb {R}}^n))$, strengthened with the ${{\,\mathrm{BQP}\,}}(U)$-constraints in ${\mathcal {R}}$, can be equivalently written as

38 $$\begin{aligned} \begin{array}{r@{\ }l@{\quad }l} \text {maximize}&{}\alpha ([0, \infty ))\\ &{}\int _0^\infty \Omega _n(t)\, d\alpha (t) = 0,\\ &{}\int _0^\infty r(t)\, d\alpha (t) \ge \beta \quad \text {for} (r, \beta ) \in {\mathcal {R}},\\ &{}\begin{pmatrix} 1&{}\ \alpha ([0, \infty ))\\ \alpha ([0, \infty ))&{}\ \alpha (\{0\}) \end{pmatrix}\text { is positive semidefinite,}\\ &{}\alpha \text { is a finite Borel measure on}~[0, \infty ). \end{array} \end{aligned}$$

A dual for this problem is the following optimization problem on variables $\lambda$, $y(r, \beta )$ for $(r, \beta ) \in {\mathcal {R}}$, and $z_1$, $z_2$, $z_3$:

39 $$\begin{aligned} \begin{array}{r@{\ }l@{\quad }l} \text {minimize}&{}z_1 + \sum _{(r, \beta ) \in {\mathcal {R}}} y(r, \beta ) \beta \\ &{}\lambda + \sum _{(r, \beta ) \in {\mathcal {R}}} y(r, \beta ) r(0) + z_2 + z_3 \ge 1,\\ &{}\lambda \Omega _n(t) + \sum _{(r, \beta ) \in {\mathcal {R}}} y(r, \beta ) r(t) + z_2 \ge 1\quad \text {for}~t > 0,\\ &{}\begin{pmatrix} z_1&{}-\frac{1}{2} z_2\\ -\frac{1}{2}z_2&{}-z_3 \end{pmatrix}\text { is positive semidefinite,}\\ &{}{y \le 0.} \end{array} \end{aligned}$$

Again, this is the problem that we solve to obtain an upper bound, and the two reasons for this are the same as before. The first one comes from weak duality: the objective value of any feasible solution of this problem is an upper bound for the independence density. Indeed, let $\lambda$, *y*, $z_1$, $z_2$, $z_3$ be a feasible solution of (39) and $\alpha$ be a feasible solution of (38). Then

$$\begin{aligned} \begin{aligned} z_1 + \sum _{(r, \beta ) \in {\mathcal {R}}} y(r, \beta ) \beta&\ge z_1 + \sum _{(r, \beta ) \in {\mathcal {R}}} y(r, \beta ) \int _0^\infty r(t)\, d\alpha (t)\\&= z_1 + \int _0^\infty \sum _{(r, \beta ) \in {\mathcal {R}}} y(r, \beta ) r(t)\, d\alpha (t)\\&\ge z_1 + \alpha (\{0\})(-z_3) + \int _0^\infty 1 - \lambda \Omega _n(t) - z_2\, d\alpha (t)\\&=z_1 - z_3 \alpha (\{0\}) + (1-z_2) \alpha ([0, \infty )) - \lambda \int _0^\infty \Omega _n(t)\, d\alpha (t)\\&=z_1 - z_3 \alpha (\{0\}) -z_2 \alpha ([0, \infty )) + \alpha ([0,\infty ))\\&\ge \alpha ([0, \infty )), \end{aligned} \end{aligned}$$

as we wanted.

The second reason is that the dual is a semidefinite program with finitely many variables, though infinitely many constraints, including one constraint for each $t > 0$. In practice, we discretize the set of constraints and solve a finite semidefinite program, later on proving that a suitable modification of the solution found is indeed feasible for the infinite problem, as we discuss now.

### Finding feasible dual solutions and checking them

To find good feasible solutions of (39), we start by taking ${\mathcal {R}}= \emptyset$. Then we discretize the constraint set: we choose a finite sample ${\mathcal {S}}\subseteq (0, \infty )$ and instead of all constraints for $t > 0$ we only consider constraints for $t \in {\mathcal {S}}$. Then we have a semidefinite program, which we solve using standard semidefinite programming solvers. The idea is that, if the sample ${\mathcal {S}}$ is fine enough, then the solution found will be close enough to being feasible, and so by slightly increasing $z_1$ and $z_2$ we will be able to find a feasible solution.

By solving the discretized dual problem we obtain at the same time an optimal solution of the discretized primal problem, in which $\alpha$ is a sum of Dirac $\delta$ measures supported on ${\mathcal {S}}\cup \{0\}$ (notice this is likely not an optimal solution of the original primal problem, but of the discretized one). We use this primal solution to perform a *separation round*, that is, to look for violated ${{\,\mathrm{BQP}\,}}(U)$-constraints that we can add to the problem. One way to do this is as follows.

Say that $\alpha$ is the primal solution and let

$$\begin{aligned} f(x) = \int _0^\infty \Omega _n(t\Vert x\Vert )\, d\alpha (t). \end{aligned}$$

Fix an integer $N \ge 2$, write $[N] = \{1, \ldots , N\}$, and let $Z \in {\mathbb {R}}^{N \times N}$, $\beta \in {\mathbb {R}}$ be such that $\langle Z, A \rangle \ge \beta$ is valid for ${{\,\mathrm{BQP}\,}}([N])$. Then we try to find points $x_1$, ..., $x_N \in {\mathbb {R}}^n$ that maximize the violation

40 $$\begin{aligned} \beta - \sum _{i,j=1}^N Z(i, j) f(x_i - x_j) \end{aligned}$$

of the ${{\,\mathrm{BQP}\,}}(U)$-constraint. If we find points such that the violation is positive, then we have a violated constraint which can be added to ${\mathcal {R}}$; the whole procedure can then be repeated: the dual problem is solved again and a new separation round is performed. To find violated constraints we work with a list of facets of ${{\,\mathrm{BQP}\,}}([N])$, as in Sect. 8.3. To find points $x_1$, ..., $x_N \in {\mathbb {R}}^n$ maximizing (40) we simply use some method for unconstrained optimization.

After a few optimization/separation rounds, one starts to notice only minor improvements to the bound. Then it is time to check how far from feasible the dual solution is and to fix it in order to get a truly feasible solution and therefore an upper bound. The verification procedure for the dual solution has already been outlined by Keleti et al. [22] and will be omitted here; the dual solutions that give the bounds in Table 2 and a program to verify them can be found together with the arXiv version of this paper.

## Sets avoiding many distances in ${\mathbb {R}}^n$ and the computability of the independence density

Reassuring though Theorem 5.1 may be, the computational results of Sects. 8 and 9 do not use it, or rather use only the easy direction of the statement. In this section we will see how the full power of Theorem 5.1 can be used to recover results about densities of sets avoiding several distances in Euclidean space.

Furstenberg et al. [17] showed that, if $n \ge 2$, then any subset of ${\mathbb {R}}^n$ with positive upper density realizes all arbitrarily large distances. More precisely, if $I \subseteq {\mathbb {R}}^n$ has positive upper density, then there is $d_0 > 0$ such that for all $d > d_0$ there are *x*, $y \in I$ with $\Vert x-y\Vert =d$. This fails for $n = 1$: the set $\bigcup _{k \in {\mathbb {Z}}} (2k, 2k+1)$ has density 1/2 but does not realize any odd distance.

Falconer [14] proved the following related theorem: if $(d_m)$ is a sequence of positive numbers that converges to 0, then for all $n \ge 2$

$$\begin{aligned} \lim _{m\rightarrow \infty } \alpha _{{\bar{\delta }}}(G({\mathbb {R}}^n, \{d_1, \ldots , d_m\})) = 0. \end{aligned}$$

This theorem also fails when $n = 1$, as can be seen from an adaptation of the previous example.

Bukh [6] proved a theorem that implies both theorems above; namely, he showed that, as the ratios $d_2 / d_1$, ..., $d_m / d_{m-1}$ between the distances $d_1$, ..., $d_m$ go to infinity, so does $\alpha _{{\bar{\delta }}}(G({\mathbb {R}}^n, \{d_1, \ldots , d_m\}))$ go to $\alpha _{{\bar{\delta }}}(G({\mathbb {R}}^n, \{1\}))^m$, provided $n \ge 2$. More precisely, for every $n \ge 2$ and every $m \ge 2$,

41 $$\begin{aligned} \lim _{q \rightarrow \infty } \sup \{\, \alpha _{{\bar{\delta }}}(G({\mathbb {R}}^n, \{d_1, \ldots , d_m\})) : d_k / d_{k-1} > q\,\} = \alpha _{{\bar{\delta }}}(G({\mathbb {R}}^n, \{1\}))^m. \end{aligned}$$

Oliveira and Vallentin [36] showed that the limit above decreases exponentially fast as *m* increases. They showed that

$$\begin{aligned} \lim _{q \rightarrow \infty } \sup \{\, \vartheta (G({\mathbb {R}}^n, \{d_1, \ldots , d_m\}), {{\,\mathrm{PSD}\,}}({\mathbb {R}}^n)) : d_k / d_{k-1} > q\,\} \le 2^{-m}, \end{aligned}$$

using in the proof only a few properties of the Bessel function. In this section, we will see how Bukh’s result (41) can be obtained in a similar fashion using Theorem 5.1. This illustrates how the completely positive formulation provides a good enough characterization of the independence density to allow us to prove such precise asymptotic results.

Bukh derives his asymptotic result from an algorithm to compute the independence density to any desired precision. As a by-product of the approach of this section we also obtain such an algorithm based on solving a sequence of stronger and stronger convex optimization problems.

Finally, similar decay results can be proved for distance graphs on other metric spaces, such as the sphere or the real or complex projective space [35]. The methods of this section can in principle be applied to any metric space, as long as the harmonic analysis can be tackled successfully.

### Thick constraints

The better bounds for the independence density described in Sect. 9 were obtained by adding to the initial problem $\vartheta (G({\mathbb {R}}^n,\{1\}), {{\,\mathrm{PSD}\,}}({\mathbb {R}}^n))$ a few ${{\,\mathrm{BQP}\,}}(U)$-constraints for finite sets *U*. Our approach in this section is similar: we wish to add more and more constraints to the initial problem in a way that is guaranteed to give us closer and closer approximations of the independence density. The constraints used in Sect. 9 are easy to deal with in computations, but it is not clear (and we do not know) whether by adding a finite number of them to the initial problem we can get arbitrarily close to the independence density. A slight modification of these constraints, however, displays this property, even though such modified constraints are much harder to deal with in practice.

For a finite set $U \subseteq {\mathbb {R}}^n$ write

$$\begin{aligned} m(U) = \min \{\,\Vert x-y\Vert : x, y \in U,\ x \ne y\,\} \end{aligned}$$

for the minimum distance between pairs of distinct points in *U*. The following lemma provides an alternative characterization of ${\mathcal {C}}({\mathbb {R}}^n)$.

#### Lemma 10.1

A continuous and real-valued function $f \in L^\infty ({\mathbb {R}}^n)$ belongs to ${\mathcal {C}}({\mathbb {R}}^n)$ if and only if

42 $$\begin{aligned} \sum _{x,y\in U} Z(x, y) \int _{B(x, \delta )} \int _{B(y, \delta )} f(x'-y')\, dy'dx' \ge 0 \end{aligned}$$

for all finite $U \subseteq {\mathbb {R}}^n$, $Z \in {\mathcal {C}}^*(U)$, and $0 < \delta \le m(U)/2$.

Compare this lemma to the definition of ${\mathcal {C}}({\mathbb {R}}^n)$ from Sect. 6.3. A constraint (42) is obtained from

$$\begin{aligned} \sum _{x, y \in U} Z(x, y) f(x-y) \ge 0 \end{aligned}$$

by considering an open ball of radius $\delta$ around each point in *U*; since $\delta \le m(U)/2$, balls around different points do not intersect. So we are “thickening” each point in *U*.

#### Proof

Let $f \in L^\infty ({\mathbb {R}}^n)$ be a continuous and real-valued function and suppose there is a finite $U \subseteq {\mathbb {R}}^n$ and $Z \in {\mathcal {C}}^*(U)$ such that

$$\begin{aligned} \sum _{x, y \in U} Z(x, y) f(x - y) < 0. \end{aligned}$$

Since *f* is continuous, for every $\epsilon > 0$ there is $\delta > 0$ such that for all *x*, $y \in U$ we have $|f(x - y) - f(x' - y')| < \epsilon$ for all $x' \in B(x, \delta )$ and $y' \in B(y, \delta )$. So for all *x*, $y \in U$ one has

$$\begin{aligned} \begin{aligned}&\biggl |f(x - y) - ({{\,\mathrm{vol}\,}}B(0, \delta ))^{-2} \int _{B(x, \delta )} \int _{B(y, \delta )} f(x' - y')\, dy'dx'\biggr |\\&\qquad \le ({{\,\mathrm{vol}\,}}B(0, \delta ))^{-2} \int _{B(x, \delta )} \int _{B(y, \delta )} |f(x - y) - f(x' - y')|\, dy' dx'\\&\qquad <\epsilon . \end{aligned} \end{aligned}$$

It follows that, by taking $\epsilon$ small enough, the left-hand side of (42) for the corresponding $\delta$ will be negative.

For the other direction, we approximate integrals of *f* by finite sums. If *f* is such that the left-hand side of (42) is negative, then take for $U'$ the set consisting of a fine sample of points inside each $B(x, \delta )$ for $x \in U$. In this way one approximates by summation the double integrals in (42), showing that

$$\begin{aligned} \sum _{x, y \in U'} Z'(x, y) f(x - y) < 0, \end{aligned}$$

where $Z':U' \times U' \rightarrow {\mathbb {R}}$ is the copositive matrix derived from *Z* by duplication of rows and columns. $\square$

Recall from Sect. 9.1 that a continuous radial function $f \in L^\infty ({\mathbb {R}}^n)$ of positive type can be represented by a finite Borel measure $\alpha$ on $[0, \infty )$ via

$$\begin{aligned} f(x) = \int _0^\infty \Omega _n(t \Vert x\Vert )\, d\alpha (t). \end{aligned}$$

Using this expression, a constraint like (42) becomes

$$\begin{aligned} \int _0^\infty r(t)\, d\alpha (t), \end{aligned}$$

where $r:[0, \infty ) \rightarrow {\mathbb {R}}$ is the function such that

43 $$\begin{aligned} r(t) = \sum _{x,y \in U} Z(x, y) \int _{B(x, \delta )} \int _{B(y, \delta )} \Omega _n(t \Vert x'-y'\Vert )\, dy' dx'; \end{aligned}$$

note *r* is continuous. The following lemma establishes two key properties of such a function *r*.

#### Lemma 10.2

If *r* is given as in (43), then *r* vanishes at infinity. If moreover $n \ge 2$ and ${{\,\mathrm{tr}\,}}Z \ne 0$, then $r(t) \ge 0$ for all large enough *t*.

#### Proof

Let *B* be an open ball centered at the origin and fix $z \in {\mathbb {R}}^n$. Let $\mu$ be the Haar measure on the orthogonal group $\mathrm {O}(n) \subseteq {\mathbb {R}}^{n \times n}$, normalized so the total measure is 1. Averaging over $\mathrm {O}(n)$ the Fourier transform (on the space ${\mathbb {R}}^{2n}$) of the characteristic function $\chi _{B \times (z+B)}$ of $B \times (z + B)$ we get

$$\begin{aligned} \begin{aligned}&\int _{\mathrm {O}(n)} {\widehat{\chi }}_{B \times (z+B)}(Tu, -Tu)\, d\mu (T)\\&\qquad =\int _{\mathrm {O}(n)} \int _{{\mathbb {R}}^n} \int _{{\mathbb {R}}^n} \chi _B(x) \chi _{z+B}(y) e^{-i (Tu \cdot x - Tu \cdot y)}\, dy dx d\mu (T)\\&\qquad = \int _{{\mathbb {R}}^n} \int _{{\mathbb {R}}^n} \chi _B(x) \chi _{z+B}(y) \int _{\mathrm {O}(n)} e^{-i Tu \cdot (x - y)}\, d\mu (T) dy dx\\&\qquad = \int _B \int _{z+B} \Omega _n(\Vert u\Vert \Vert x-y\Vert )\, dy dx, \end{aligned} \end{aligned}$$

which provides us with an expression for the double integrals appearing in (43) in terms of the Fourier transform of $\chi _{B \times (z + B)}$; the lemma will follow from this relation.

First, it is immediate from this relation that *r* vanishes at infinity. Indeed, the Riemann–Lebesgue lemma [38, Theorem IX.7] says that the Fourier transform of the characteristic function vanishes at infinity (that is, as $\Vert u\Vert \rightarrow \infty$) and so, since *Z* is a fixed matrix, we must have that *r* vanishes at infinity.

To see that *r* is nonnegative at infinity is only slightly more complicated. Note

$$\begin{aligned} {\widehat{\chi }}_{B\times (z+B)}(u, -u) = e^{iu\cdot z} {\widehat{\chi }}_{B \times B}(u, -u). \end{aligned}$$

Since *B* is centered at the origin, ${\widehat{\chi }}_{B \times B}(Tu, -Tu) = {\widehat{\chi }}_{B \times B}(u, -u)$ for all $T \in \mathrm {O}(n)$, so averaging gives us

44 $$\begin{aligned} \begin{aligned} \int _B \int _{z+B} \Omega _n(\Vert u\Vert \Vert x-y\Vert )\, dy dx&= \int _{\mathrm {O}(n)} e^{i Tu\cdot z} {\widehat{\chi }}_{B \times B}(Tu, -Tu)\, d\mu (T)\\&= \int _{\mathrm {O}(n)} e^{i Tu\cdot z} {\widehat{\chi }}_{B \times B}(u, -u)\, d\mu (T)\\&=\Omega _n(\Vert u\Vert \Vert z\Vert ) {\widehat{\chi }}_{B \times B}(u, -u). \end{aligned} \end{aligned}$$

Recall that $\Omega _n(0) = 1$. Since $n \ge 2$, the function $\Omega _n$ vanishes at infinity.^6^ Then, since ${{\,\mathrm{tr}\,}}Z \ne 0$, and hence ${{\,\mathrm{tr}\,}}Z > 0$ as *Z* is copositive, using (44) it follows that for all large *t* the diagonal summands in (43) together dominate the off-diagonal ones.

Now ${\widehat{\chi }}_{B \times B}(u, -u) \ge 0$ as follows from the definition of the Fourier transform. So since ${{\,\mathrm{tr}\,}}Z > 0$, it follows that for all large enough *t* we have $r(t) \ge 0$. $\square$

Say now ${\mathcal {R}}$ is any finite collection of functions *r* each one defined in terms of a thick constraint as in (43), and let $d_1$, ..., $d_m$ be *m* distinct positive numbers. Consider the optimization problem

45 $$\begin{aligned} \begin{array}{r@{\ }l@{\quad }l} \text {maximize}&{}\alpha (\{0\})\\ &{}\alpha ([0, \infty )) = 1,\\ &{}\int _0^\infty \Omega _n(d_i t)\, d\alpha (t) = 0\qquad \text {for}~i = 1, \dots ,~m,\\ &{}\int _0^\infty r(t)\, d\alpha (t) \ge 0\qquad \hbox { for}~\ r \in {\mathcal {R}},\\ &{}{\alpha \text {is a Borel measure on}~[0, \infty ).} \end{array} \end{aligned}$$

This problem is comparable to (38), but instead of using the alternative normalization of Sect. 7.2, the standard normalization is used, and instead of considering only distance 1 as a forbidden distance, distances $d_1$, ..., $d_m$ are forbidden; this way we get an infinite-dimensional linear program instead of a semidefinite program. By construction, the optimal value of (45) is an upper bound for $\alpha _{{\bar{\delta }}}(G({\mathbb {R}}^n, \{d_1, \ldots , d_m\}))$.

A dual problem for (45) is the following (cf. problem (39)):

46 $$\begin{aligned} \begin{array}{r@{\ }l@{\quad }l} \text {minimize}&{}\lambda \\ &{}\lambda + \sum _{i=1}^m z_i + \sum _{r \in {\mathcal {R}}} y(r) r(0) \ge 1,\\ &{}\lambda + \sum _{i=1}^m z_i\Omega _n(d_i t) + \sum _{r \in {\mathcal {R}}} y(r) r(t) \ge 0&{}\text {for all}~t > 0,\\ &{}y \le 0. \end{array} \end{aligned}$$

(Recall $\Omega _n(0) = 1$, hence the coefficient of $z_i$ in the first constraint is 1.) Weak duality holds between (45) and (46): if $\lambda$, *z*, and *y* is any feasible solution of the dual problem and $\alpha$ is any feasible solution of the primal problem, then $\alpha (\{0\}) \le \lambda$; the proof of this fact is analogous to the proof of the weak duality relation between problems (38) and (39), given in Sect. 9.2. So any feasible solution $\lambda$, *z*, and *y* of the dual provides an upper bound for the independence density, namely

$$\begin{aligned} \alpha _{{\bar{\delta }}}(G({\mathbb {R}}^n, \{d_1, \ldots , d_m\})) \le \lambda . \end{aligned}$$

### A sequence of primal problems

For each finite nonempty set *U*, the set

$$\begin{aligned} {\mathcal {T}}^*(U) = \{\, Z \in {\mathcal {C}}^*(U) : \Vert Z\Vert _1 \le 1\,\}, \end{aligned}$$

the *tip* of ${\mathcal {C}}^*(U)$, is a compact convex set, and every copositive matrix is a multiple of a matrix in the tip.^7^ There is then a countable dense subset ${\mathcal {T}}^*_{\aleph _0}(U)$ of ${\mathcal {T}}^*(U)$, and we may assume that all $Z \in {\mathcal {T}}^*_{\aleph _0}(U)$ are such that ${{\,\mathrm{tr}\,}}Z > 0$ and $\langle J, Z\rangle > 0$.

If $U \subseteq {\mathbb {R}}^n$ is finite, then the set of constraints of the form (42) with $Z \in {\mathcal {T}}^*_{\aleph _0}(U)$ and $\delta = m(U)/(2k)$ for integer $k \ge 1$ is countable. If we consider all finite subsets *U* of ${\mathbb {Q}}^n$ and all corresponding constraints, then the set of all constraints thus obtained is also countable. The corresponding functions (43) can be enumerated as $r_1$, $r_2$, .... We use this enumeration to define a sequence of optimization problems, the *N*th one being

47 $$\begin{aligned} \begin{array}{r@{\ }l@{\quad }l} \text {maximize}&{}\alpha (\{0\})\\ &{}\alpha ([0, \infty )) = 1,\\ &{}\int _0^\infty \Omega _n(t)\, d\alpha (t) = 0,\\ &{}\int _0^\infty r_k(t)\, d\alpha (t) \ge 0\qquad \text {for}~1 \le k \le N,\\ &{}{\alpha \text { is a Borel measure on}~[0, \infty )}. \end{array} \end{aligned}$$

Note this is just problem (45) with ${\mathcal {R}}= \{ r_1, \ldots , r_N\}$, $m = 1$, and $d_1 = 1$. Let $\vartheta _N$ denote both the *N*th optimization problem above and its optimal value, and denote by $\vartheta _\infty$ the optimization problem in which constraints for all $k \ge 1$ are added, as well as the optimal value of this problem. We know that $\vartheta _N \ge \alpha _{{\bar{\delta }}}(G({\mathbb {R}}^n, \{1\}))$ for all $N \ge 1$. By the construction of the $r_k$ functions, using Lemma 10.1 and Theorem 6.3, we also know that $\vartheta _\infty = \alpha _{{\bar{\delta }}}(G({\mathbb {R}}^n, \{1\}))$.

#### Theorem 10.3

If $$n \ge 2$$, then $$\lim _{N \rightarrow \infty } \vartheta _N = \vartheta _\infty $$.

#### Proof

Since $\vartheta _N \ge \vartheta _{N+1}$ and $\vartheta _N \ge \vartheta _\infty$ for all $N \ge 1$, the limit exists and is at least $\vartheta _\infty$; we show now the reverse inequality.

So let $(\alpha _N)$ be a sequence of measures such that $\alpha _N$ is a feasible solution of $\vartheta _N$ and $\alpha _N(\{0\}) \ge L$ for all $N \ge 1$ and some $L > 0$. Each $\alpha _N$ is a finite Radon measure (since $[0, \infty )$ is a complete separable metric space), being therefore an element of the space $M([0, \infty ))$ of signed Radon measures of bounded total variation. By the Riesz Representation Theorem [16, Theorem 7.17], the space $M([0, \infty ))$ is the dual space of $C_0([0, \infty ))$, which is the space of continuous functions vanishing at infinity equipped with the supremum norm.

For $f \in C_0([0, \infty ))$ and $\mu \in M([0, \infty ))$, write

$$\begin{aligned}{}[f, \mu ] = \int _0^\infty f(t)\, d\mu (t). \end{aligned}$$

If $\Vert f\Vert _\infty \le 1$, then $|[f, \alpha _N]| \le 1$ since $\alpha _N([0, \infty )) = 1$. So all $\alpha _N$ belong to the closed unit ball

$$\begin{aligned} \{\, \mu \in M([0, \infty )) : |[f, \mu ]| \le 1 \text { for all}~f \in C_0([0, \infty )) \text { with}~\Vert f\Vert _\infty \le 1\,\}, \end{aligned}$$

which by Alaoglu’s theorem [16, Theorem 5.18] is compact in the weak-$*$ topology on $M([0, \infty ))$.

So $(\alpha _N)$ has a weak-$*$-convergent subsequence^8^; let us assume that the sequence itself converges to a measure $\alpha \in M([0, \infty ))$. Here is what we want to prove:

(i) $$\alpha (\{0\}) \ge \lim _{N \rightarrow \infty } \alpha _N(\{0\})$$;
(ii) $$\alpha ([0, \infty )) \le 1$$;
(iii) $\alpha ([0, \infty ))^{-1} \alpha$ is a feasible solution of $\vartheta _\infty$.

From these three claims the reverse inequality, and hence the theorem, follows.

To see (i), note first that $\alpha$ must be nonnegative. For suppose $\alpha (X) < 0$ for some set *X*. Since $\alpha$ is Radon, it is inner regular on $\sigma$-finite sets [16, Proposition 7.5], so there is a compact set $C \subseteq X$ such that $\alpha (C) < 0$. For $k \ge 1$, let $U_k$ be the set of all points at distance less than 1/*k* from *C*; note that $U_k$ is open and that *C* is the intersection of $U_k$ for $k \ge 1$.

For every $k \ge 1$, Urysohn’s lemma says that there is a continuous function $f_k:[0, \infty ) \rightarrow [0,1]$ that is 1 on *C* and 0 outside of $U_k$, and since $U_k$ is bounded this function vanishes at infinity. Now $\alpha (C) = \lim _{k\rightarrow \infty } \alpha (U_k)$, so if *k* is large enough we have

$$\begin{aligned} 0 > [f_k, \alpha ] = \lim _{N \rightarrow \infty } [f_k, \alpha _N], \end{aligned}$$

and for some *N* we must have $[f_k, \alpha _N] < 0$, a contradiction since $f \ge 0$ and $\alpha _N$ is nonnegative.

Next, for every $\epsilon > 0$ let $f_\epsilon :[0, \infty ) \rightarrow [0, 1]$ be a continuous function such that $f_\epsilon (0) = 1$ and $f_\epsilon (t) = 0$ for $t \ge \epsilon$. Note that

$$\begin{aligned} \alpha (\{0\}) = \lim _{\epsilon \downarrow 0} \alpha ([0, \epsilon )). \end{aligned}$$

Now

$$\begin{aligned} \alpha ([0, \epsilon )) \ge [f_\epsilon , \alpha ] = \lim _{N \rightarrow \infty } [f_\epsilon , \alpha _N] \ge \lim _{N \rightarrow \infty } \alpha _N(\{0\}), \end{aligned}$$

proving (i).

For (ii), if $\alpha ([0, \infty )) > 1$, then there is *U* such that $\alpha ([0, U)) > 1$. Let $f:[0, \infty ) \rightarrow [0, 1]$ be a continuous function such that $f(t) = 1$ for $t \in [0, U)$ and $f(t) = 0$ for $t \ge U + 1$. Then

$$\begin{aligned} 1 < \alpha ([0, U)) \le [f, \alpha ] = \lim _{N \rightarrow \infty } [f, \alpha _N], \end{aligned}$$

and for some *N* we have $\alpha _N([0, U+1)) \ge [f, \alpha _N] > 1$, a contradiction since $\alpha _N$ is feasible for $\vartheta _N$.

Finally, for (iii), recall that $\Omega _n$ vanishes at infinity for $n \ge 2$. Then

$$\begin{aligned} \int _0^\infty \Omega _n(t)\, d\alpha (t) = [\Omega _n, \alpha ] = \lim _{N \rightarrow \infty } [\Omega _n, \alpha _N] = 0. \end{aligned}$$

From Lemma 10.2 we know that $r_k$ vanishes at infinity for all *k*, so similarly we have $[r_k, \alpha ] \ge 0$ for all $k \ge 1$, finishing the proof of (iii) and that of the theorem. $\square$

### A sequence of dual problems

Following (46), here is a dual problem for $\vartheta _N$:

48 $$\begin{aligned} \begin{array}{r@{\ }l@{\quad }l} \text {minimize}&{}\lambda \\ &{}\lambda + z + \sum _{k=1}^N y_k r_k(0) \ge 1,\\ &{}\lambda + z \Omega _n(t) + \sum _{k=1}^N y_k r_k(t) \ge 0&{}\text {for all}~t > 0,\\ &{}y \le 0. \end{array} \end{aligned}$$

Weak duality holds between this problem and $\vartheta _N$, but in this case we know even more, namely that there is no duality gap between primal and dual problems:

#### Theorem 10.4

If $n \ge 2$, then the optimal value of (48) is $\vartheta _N$.

In Sect. 9.3 we saw how problem (39), which is similar to (48), is solved: we disregard all constraints for $t > L$ for some $L > 0$, take a finite sample ${\mathcal {S}}$ of points in [0, *L*], and consider only constraints for $t \in {\mathcal {S}}$. We then have a finite linear program, which can be solved by computer. Most likely, an optimal solution of this problem will be (slightly) infeasible for the original, infinite problem. However, the hope is that, if *L* is large enough and the sample ${\mathcal {S}}$ is fine enough, then the solution obtained from the discretized problem can be fixed to become a feasible solution of the original problem.

The proof of the above theorem follows the same strategy, but while in Sect. 9.3 we did not have to argue that this solution strategy *always* works (since we were only interested in having it work for the cases considered), here we have to. For that we need two lemmas, the first one to help us find the number *L*.

#### Lemma 10.5

If $n \ge 2$ and if $t_0 > 0$ is such that $\Omega _n(t_0) < 0$ and $r_k(t_0) \ge 0$ for $k = 1$, ..., *N*, then the polyhedron in ${\mathbb {R}}^{N+2}$ consisting of vectors $(\lambda , z, y_1, \ldots , y_N)$ satisfying

49 $$\begin{aligned} \begin{array}{l} -1 \le \lambda \le 2,\\ y_k \le 0\quad \hbox {for}~ k = 1 {, \dots ,}~ N,\\ \lambda + z + \sum _{k=1}^N y_k r_k(0) \ge 1,\\ \lambda + z\Omega _n(t_0) + \sum _{k=1}^N y_k r_k(t_0) \ge 0 \end{array} \end{aligned}$$

is bounded.

Note that such a $t_0$ as in the statement above exists, as follows from Lemma 10.2 since $\Omega _n$ has zeros of arbitrarily large magnitude.^9^

#### Proof

Let ${\mathcal {K}}\subseteq {\mathbb {R}}^{N+2}$ be the cone generated by the $N+4$ vectors

$$\begin{aligned} \begin{array}{l} l_1 = (1, 0, \ldots , 0),\\ l_2 = (-1, 0, \ldots , 0),\\ e_1 = (0, 0, -1, \ldots , 0), e_2 = (0, 0, 0, -1, \ldots , 0), \ldots , e_N = (0, 0, 0, \ldots , -1),\\ s_1 = (1, 1, r_1(0), \ldots , r_N(0)),\\ s_2 = (1, \Omega _n(t_0), r_1(t_0), \ldots , r_N(t_0)). \end{array} \end{aligned}$$

The polyhedron given by the inequalities (49) is bounded if and only if ${\mathcal {K}}= {\mathbb {R}}^{N+2}$; let us show that this is the case.^10^

By construction we have $r_k(0) > 0$ (recall that the copositive matrix *Z* used in the definition of $r_k$ is such that $\langle J, Z\rangle > 0$; see Sect. 10.2); add nonnegative multiples of $l_2$, $e_1$, ..., $e_N$ to $s_1$ to get $w_1 = (0, 1, 0, \ldots , 0) \in {\mathcal {K}}$. Since $r_k(t_0) \ge 0$, add nonnegative multiples of $l_2$, $e_1$, ..., $e_N$ to $s_2$ and rescale the result to see that $-w_1 \in {\mathcal {K}}$.

Finally, for each $k = 1$, ..., *N*, add to $s_1$ nonnegative multiples of $l_2$, $-w_1$, and $e_i$ for $i \ne k$ and rescale the result to see that $-e_k \in {\mathcal {K}}$, finishing the proof that ${\mathcal {K}}= {\mathbb {R}}^{N+2}$. $\square$

The second lemma provides some crude bounds on the derivative of the functions $\Omega _n$ and $r_k$, and will be used to help us decide how fine the sample ${\mathcal {S}}$ has to be.

#### Lemma 10.6

If $n \ge 2$, then for all $t \ge 0$ we have $|\Omega '_n(t)| \le \Gamma (n / 2)$. If *r* is given as in (43), then

$$\begin{aligned} |r'(t)| \le \sum _{x,y \in U} |Z(x, y)| (\Vert x-y\Vert +2\delta )({{\,\mathrm{vol}\,}}B(0,\delta ))^2 \Gamma (n / 2). \end{aligned}$$

#### Proof

It follows directly from the series expansion of the Bessel function of order $\alpha$ that

$$\begin{aligned} \frac{d t^{-\alpha } J_\alpha (t)}{dt} = -t^{-\alpha } J_{\alpha +1}(t), \end{aligned}$$

and so from (37) we get

$$\begin{aligned} \Omega '_n(t) = -\Gamma \Bigl (\frac{n}{2}\Bigr ) \Bigl (\frac{2}{t}\Bigr )^{(n-2)/2} J_{n/2}(t). \end{aligned}$$

Compare this with the expression for $\Omega _{n+2}$ to get

$$\begin{aligned} \Omega '_n(t) = -(t/n) \Omega _{n+2}(t). \end{aligned}$$

Now $|J_\alpha (t)| \le 1$ for all $\alpha \ge 0$ and $t \ge 0$ [47, equation (10), §13.42]. Combine this with the first expression for $\Omega '_n$ to see that for $t \ge 2$ we have $|\Omega '_n(t)| \le \Gamma (n/2)$. From the definition (36) of $\Omega _n$, it follows that $|\Omega _n(t)| \le 1$ for all *t*, hence from the second expression for $\Omega '_n$ it is clear that $|\Omega '_n(t)| \le 2 / n$ for $t \le 2$. For $n \ge 2$ we have $\Gamma (n/2) \ge 2 / n$, and so $|\Omega '_n(t)| \le \Gamma (n/2)$.

For the estimate on $r'$, take *x*, $y \in U$. Then

$$\begin{aligned} \begin{aligned}&\biggl |\frac{d}{dt} \int _{B(x, \delta )} \int _{B(y, \delta )} \Omega _n(t\Vert x'-y'\Vert )\, dy' dx'\biggr |\\&\qquad =\biggl |\int _{B(x, \delta )} \int _{B(y, \delta )} \frac{d\Omega _n(t\Vert x'-y'\Vert )}{dt}\, dy' dx'\biggr |\\&\qquad \le \int _{B(x, \delta )} \int _{B(y, \delta )} \Vert x'-y'\Vert |\Omega _n'(t\Vert x'-y'\Vert )|\, dy' dx'\\&\qquad \le (\Vert x-y\Vert +2\delta )({{\,\mathrm{vol}\,}}B(0, \delta ))^2 \Gamma (n/2), \end{aligned} \end{aligned}$$

and the estimate for $r'$ follows. $\square$

We now have everything needed to prove that there is no duality gap.

#### Proof of Theorem 10.4

Fix $\epsilon > 0$ and let $t_0$ be such that $\Omega _n(t_0) < 0$ and $r_k(t_0) \ge 0$ for all $k = 1$, ..., *N*. Lemma 10.5 says that the polyhedron described by the inequalities (49) is bounded; let *M* be an upper bound on the Euclidean norm of any vector in this polyhedron. Since $\Omega _n$ vanishes at infinity and so does $r_k$ for all *k* (cf. Lemma 10.2), there is $L \ge t_0$ such that

50 $$\begin{aligned} \Vert (\Omega _n(t), r_1(t), \ldots , r_N(t))\Vert \le \epsilon / M\qquad \text {for all}~t \ge L. \end{aligned}$$

Lemma 10.6 implies that there is a constant *D* such that

51 $$\begin{aligned} \Vert (\Omega '_n(t), r'_1(t), \ldots , r'_k(t))\Vert \le D\qquad \text {for all}~t \ge 0. \end{aligned}$$

Let ${\mathcal {S}}\subseteq [0, L]$ be a finite set of points with the property that given $t \in [0, L]$ there is $s \in {\mathcal {S}}$ with $|t-s| \le \epsilon / (M D)$ and make sure that both $t_0$ and *L* are in ${\mathcal {S}}$.

Now consider the optimization problem

52 $$\begin{aligned} \begin{array}{r@{\ }l@{\quad }l} \text {minimize}&{}\lambda \\ &{}\lambda + z + \sum _{k=1}^N y_k r_k(0) \ge 1,\\ &{}\lambda + z \Omega _n(t) + \sum _{k=1}^N y_k r_k(t) \ge 0&{}\text {for all}~t \in {\mathcal {S}},\\ &{}-1 \le \lambda \le 2,\\ &{}y \le 0, \end{array} \end{aligned}$$

which is a finite linear program. Let $\lambda$, *z*, and *y* be an optimal solution of this problem and write

$$\begin{aligned} g(t) = z\Omega _n(t) + \sum _{k=1}^N y_k r_k(t). \end{aligned}$$

Since $t_0 \in {\mathcal {S}}$, we know from Lemma 10.5 that $\Vert (z, y_1, \ldots , y_N)\Vert \le M$. Using the Cauchy–Schwarz inequality together with (50) we see that, for all $t \ge L$,

53 $$\begin{aligned} |g(t)| \le M (\epsilon / M) = \epsilon . \end{aligned}$$

Given $t \in [0, L]$, there is $s \in {\mathcal {S}}$ such that $|t-s| \le \epsilon / (M D)$. Then using the mean-value theorem, the Cauchy–Schwarz inequality, and (51) we get

54 $$\begin{aligned} |g(t) - g(s)| \le |t-s| M D \le \epsilon . \end{aligned}$$

Since $\lambda + g(s) \ge 0$, we then have that $\lambda + g(t) \ge -\epsilon$.

The estimates (53) and (54) together show that $\lambda + \epsilon$, *z*, and *y* is a feasible solution of (48). We now find a solution of $\vartheta _N$, defined in (47), of value close to it.

To do so, notice that if $\epsilon$ is small enough, then (53) implies in particular that $\lambda > -1$, or else $\lambda + g(L) < 0$, a contradiction. Since our solution is optimal, we must also have $\lambda < 2$ (notice $\lambda = 1$, $z = 0$, and $y = 0$ is a feasible solution of our problem).

Now problem (52) is a finite linear program, and we can apply the strong duality theorem. Its dual looks very much like problem $\vartheta _N$, except that the measure $\alpha$ is now a discrete measure supported on ${\mathcal {S}}\cup \{0\}$ and there are two extra variables corresponding to the constraints $\lambda \ge -1$ and $\lambda \le 2$. Since our optimal solution of (52) is such that $-1< \lambda < 2$, complementary slackness implies that these two extra variables of the dual of (52) will be 0 in an optimal solution. So if $\alpha$ is an optimal solution of the dual of (52), then it is also a feasible (though likely not optimal) solution of $\vartheta _N$.

We have then a solution of $\vartheta _N$ of value $\lambda$ and a feasible solution of (48) of value $\lambda + \epsilon$. Making $\epsilon$ approach 0 we obtain the theorem. $\square$

### Asymptotics for many distances

The theorem below implies the ‘$\le$’ direction of Bukh’s result (41). The reverse inequality is much simpler to prove; the reader is referred to Bukh’s paper [6].

#### Theorem 10.7

If $n \ge 2$ and $m \ge 2$, then for every $\epsilon > 0$ there is *q* such that if $d_1$, ..., $d_m$ are positive numbers such that $d_i / d_{i-1} > q$ for $i = 2$, ..., *m*, then

$$\begin{aligned} \alpha _{{\bar{\delta }}}(G({\mathbb {R}}^n, \{d_1, \ldots , d_m\})) \le (\alpha _{{\bar{\delta }}}(G({\mathbb {R}}^n, \{1\})) + \epsilon )^m + \epsilon (m - 1). \end{aligned}$$

#### Proof

All ideas required for the proof can be more clearly presented when only two distances are considered; for larger values of *m* one only has to use induction.

So fix $\epsilon > 0$. Theorems 6.3 and 10.3 imply that we can choose *N* such that $\vartheta _N \le \alpha _{{\bar{\delta }}}(G({\mathbb {R}}^n, \{1\})) + \epsilon /2$ and Theorem 10.4 then says that we can take a feasible solution $\lambda$, *z*, and *y* of the dual (48) of $\vartheta _N$ satisfying

$$\begin{aligned} \lambda \le \vartheta _N + \epsilon /2 \le \alpha _{{\bar{\delta }}}(G({\mathbb {R}}^n, \{1\})) + \epsilon . \end{aligned}$$

We may assume moreover that $\lambda \le 1$. Since $\lambda$ is an upper bound on the independence density of the unit-distance graph, which is positive, by taking $\epsilon$ small enough we assume that $\lambda \ge \epsilon$.

Write

$$\begin{aligned} g(t) = z\Omega _n(t) + \sum _{k=1}^N y_k r_k(t); \end{aligned}$$

note *g* is continuous. Since $(\lambda , z, y)$ is feasible, we know that $g(0) \ge 1 - \lambda$ and $g(t) \ge -\lambda$ for all $t > 0$. Now $\Omega _n$ vanishes at infinity for $n \ge 2$, and together with Lemma 10.2 this implies that *g* also vanishes at infinity, so there is $L > 0$ such that $|g(t)| \le \epsilon$ for all $t \ge L$. Since *g* is continuous at 0, we can pick $\eta > 0$ such that $g(t) \ge 1 - \lambda - \epsilon$ for all $t \in [0, \eta ]$.

Set $q = L / \eta$ and suppose $d_1$, $d_2$ are distances satisfying $d_2 / d_1 > q$. The independence density does not change if we scale the forbidden distances, so we may assume that $d_2 = 1$ and then $d_1 < q^{-1}$. Consider the function $h(t) = g(d_1 t)$. Then $\lambda ^2 + \epsilon + g(t) + \lambda h(t)$ is

(i) at least $1 + \epsilon$ if $t = 0$;
(ii) at least $\epsilon - \lambda \epsilon \ge 0$ if $t \in [0, L]$, since $0 \le \lambda \le 1$ and $d_1 t < q^{-1} t = \eta t / L \le \eta$;
(iii) at least 0 if $t \ge L$, since $\lambda \ge \epsilon$.

Now notice

$$\begin{aligned} h(t) = z\Omega _n(d_1 t) + \sum _{k=1}^N y_k r_k(d_1 t), \end{aligned}$$

where from (43)

$$\begin{aligned} \begin{aligned} r_k(d_1 t)&= \sum _{x,y \in U_k} Z_k(x, y) \int _{B(x, \delta _k)} \int _{B(y, \delta _k)} \Omega _n(d_1 t \Vert x'-y'\Vert )\, dy' dx'\\&=\sum _{x,y \in U_k} Z_k(x, y) \int _{B(x, \delta _k)} \int _{B(y, \delta _k)} \Omega _n(t \Vert d_1 x'- d_1 y'\Vert )\, dy' dx'\\&=\sum _{x,y \in U_k} Z_k(x, y) \int _{d_1 B(x, \delta _k)} \int _{d_1 B(y, \delta _k)} \Omega _n(t \Vert x'- y'\Vert ) d_1^{-2n}\, dy' dx'\\&=\sum _{x,y \in U_k} (d_1^{-2n} Z_k(x, y)) \int _{B(d_1 x, d_1 \delta _k)} \int _{B(d_1 y, d_1 \delta _k)} \Omega _n(t \Vert x'- y'\Vert )\, dy' dx'\\&=\sum _{x,y \in d_1 U_k} (d_1^{-2n} Z_k(x, y)) \int _{B(x, d_1 \delta _k)} \int _{B(y, d_1 \delta _k)} \Omega _n(t \Vert x'- y'\Vert )\, dy' dx'. \end{aligned} \end{aligned}$$

This shows that ${\tilde{r}}_k(t) = r_k(d_1 t)$ also comes from a thick constraint through (43). Write now ${\mathcal {R}}= \{ r_1, \ldots , r_N, {\tilde{r}}_1, \ldots , {\tilde{r}}_N\}$. Then from (i)–(iii) we see that

$$\begin{aligned} \begin{array}{l} {\overline{\lambda }} = \lambda ^2 + \epsilon ,\\ {\overline{z}}_1 = \lambda z,\quad {\overline{z}}_2 = z,\\ {\overline{y}}(r_k) = y_k\quad \text {for}~k = 1, \dots ,~N, \text { and}\\ {\overline{y}}({\tilde{r}}_k) = \lambda y_k\quad \text {for}~k = 1, \dots ,~N \end{array} \end{aligned}$$

is a feasible solution of (46) for distances $d_1$, $d_2$, whence

$$\begin{aligned} \alpha _{{\bar{\delta }}}(G({\mathbb {R}}^n, \{d_1, d_2\})) \le {\overline{\lambda }} = \lambda ^2 + \epsilon \le (\alpha _{{\bar{\delta }}}(G({\mathbb {R}}^n, \{1\})) + \epsilon )^2 + \epsilon , \end{aligned}$$

as we wanted. $\square$

### Computability of the independence density

The sequence of dual problems of Sect. 10.3 can be used to construct a Turing machine that computes the independence ratio of the unit-distance graph up to any prescribed precision. Here is a brief sketch of the idea.

First we describe a Turing machine that computes an increasing sequence of lower bounds for the independence density that come arbitrarily close to it.

Given $$T > 0$$, let $${\mathcal {P}}_{T, N}$$ be the partition of $$[-T, T)^n$$ consisting of all half-open cubes $$C_1 \times \cdots \times C_n$$ with

$$\begin{aligned} C_i \in \{\, [-T + 2kT / N, -T + 2(k+1)T/N) : {k = 0, \dots , N-1}\,\}. \end{aligned}$$

For each such partition let $G_{T, N}$ be the graph whose vertex set is ${\mathcal {P}}_{T, N}$ and in which two vertices *X*, *Y* are adjacent if and only there are $x \in X$ and $y \in Y$ such that $\Vert x-y\Vert = 1$. Given *T* and *N*, the finite graph $G_{T, N}$ can be computed by a Turing machine.

By construction, if ${\mathcal {I}}$ is an independent set of $G_{T, N}$, then the union *I* of all *X* in ${\mathcal {I}}$ is an independent set of the unit-distance graph with measure $|I| {{\,\mathrm{vol}\,}}[0, 2T/N]^n$ and

$$\begin{aligned} \bigcup _{v \in (2T + 1){\mathbb {Z}}^n} v + I \end{aligned}$$

is a periodic independent set of the unit-distance graph with density

55 $$\begin{aligned} \frac{|I| {{\,\mathrm{vol}\,}}[0, 2T/N]^n}{{{\,\mathrm{vol}\,}}[-T - 1/2, T + 1/2]^n}. \end{aligned}$$

We know from Sect. 6.1 that periodic independent sets can come arbitrarily close to the independence density. It is then not hard to show that by taking larger and larger *T* and larger and larger *N* one can by the above construction generate lower bounds for the independence density that can come arbitrarily close to it.

So our Turing machine simply fixes an enumeration $(T_1, N_1)$, $(T_2, N_2)$, ... of $({\mathbb {N}}{\setminus } \{0\})^2$, computes the independence number of $G_{T_i, N_i}$ for all *i*, uses (55) to get a lower bound, and outputs at each step the best lower bound found so far.

Let us now see how to construct a Turing machine that computes a decreasing sequence of upper bounds for the independence density that come arbitrarily close to it.

The idea is to find at the *N*th step a feasible solution of the dual (48) of $\vartheta _N$ with value at most $\vartheta _N + 1 / N$. This we do by mimicking the proof of Theorem 10.4: we disregard constraints for $t \ge L$ for some large *L* and we discretize the interval [0, *L*]. Following the proof of the theorem, one sees that it is possible to estimate algorithmically how large *L* has to be and how fine the discretization has to be so we obtain a feasible solution of value at most $\vartheta _N + 1 / N$.

One problem now is that we have to work with rational numbers and not real numbers. The Bessel function and all integrals involved have to be approximated by rationals, which can be done to any desired precision algorithmically. In the end, however, we are not solving the original dual problem, but an approximated version of it. Why is the solution of this approximated version close to the solution of the original version, given, that is, that the approximation is good enough? Such a result, related to what is known in linear programming as sensitivity analysis, follows from Lemma 10.5: we work with problems of bounded feasible region, so there is a universal upper bound on the magnitude of any number appearing in any feasible solution, and it is possible to show that if the input data approximates the real data well enough, then the solutions will be very close together; moreover, it is possible to estimate how good the approximation has to be.

Another problem is to see that the set $\{r_1, r_2, \ldots \}$ can be enumerated by a Turing machine. The only difficulty here is how to enumerate the set ${\mathcal {T}}^*_{\aleph _0}(U)$ for some finite set *U*. One way to do it is as follows. First, note that ${\mathcal {T}}^*(U)$ is a subset of the $L^1$ unit ball in ${\mathbb {R}}^{U \times U}$. Given $\epsilon > 0$, consider a finite $\epsilon$-net ${\mathcal {N}}_\epsilon$ for this unit ball. Let now ${\mathcal {N}}'_\epsilon$ be a finite set containing for each $A \in {\mathcal {N}}_\epsilon$ a matrix $B \in {\mathcal {T}}^*(U)$ with $\Vert B\Vert _1 \le 1$ such that $\Vert A-B\Vert _1 \le \epsilon$, if it exists. Then, since ${\mathcal {N}}_\epsilon$ is an $\epsilon$-net, for every $Z \in {\mathcal {T}}^*(U)$ there is $B \in {\mathcal {N}}'_\epsilon$ such that $\Vert Z-B\Vert _1 \le 2\epsilon$. So we may take for ${\mathcal {T}}^*_{\aleph _0}(U)$ the union of ${\mathcal {N}}'_{1/k}$ for $k \ge 1$.

It only remains to show how ${\mathcal {N}}'_\epsilon$ can be computed. Given $A \in {\mathcal {N}}_\epsilon$, we want to solve the following finite-dimensional optimization problem:

$$\begin{aligned} \begin{array}{r@{\ }l@{\quad }l} \text {minimize}&{}\Vert A-B\Vert _1\\ &{}\Vert B\Vert _1 \le 1,\\ &{}B \in {\mathcal {C}}^*(U). \end{array} \end{aligned}$$

The $L_1$ norms above can be equivalently rewritten using linear constraints, so the above problem is a conic program that can be solved with the ellipsoid method (the separation problem is NP-hard, as follows from the equivalence between separation and optimization [19], but in this case we do not care for efficiency: it is enough to have a separation algorithm for the copositive cone, and we do [18]). By solving this problem repeatedly one can construct ${\mathcal {N}}'_\epsilon$.

So we have two Turing machines, one to find better and better lower bounds, and one to find better and better upper bounds. Running the two alternately, one constructs a third Turing machine that given $\epsilon > 0$ stops when the best lower bound is $\epsilon$-close to the best upper bound found.

## Footnotes

1 For more about nets, see Folland [16].

2 The results in this section are similar to those related to step kernels in the theory of graph limits of Lovász and Szegedy [28, §4.2].

3 First, the integral is well defined as the composition $A \circ (\sigma \mapsto (\sigma x, \sigma y))$ is measurable, since *A* is measurable and the map $\sigma \mapsto (\sigma x, \sigma y)$ is continuous from the continuous action of $\Gamma$. Second, the pushforward of the Haar measure is a finite measure. Then $L^2(V \times V) \subseteq L^1(V \times V)$ [16, Exercise 5, §6.1], and Tonelli’s theorem applied to the product measure on $(V \times V) \times \Gamma$ says that $(x, y) \mapsto \int _\Gamma |A(\sigma x, \sigma y)|\, d\mu (\sigma )$, and hence $R(A)(x, y)$, exists for almost all $(x, y) \in V \times V$. One checks similarly that $R(A) \in L^2(V \times V)$.

4 Since $\Gamma$ is compact and metrizable, it is separable. This implies that $L^2(\Gamma \times \Gamma )$ is separable, and hence ${\mathcal {T}}(\Gamma )$ is metrizable; see Sect. 4.3.

5 For every $\epsilon > 0$, there is a compact set $B \subseteq {\mathbb {R}}^n$ such that $\bigl |f(x) - \int _B e^{iu\cdot x}\, d\nu (u)\bigr | < \epsilon$ for all $x \in {\mathbb {R}}^n$.

6 This follows e.g. from the asymptotic formula for the Bessel function [47, equation (1), §7.21] and is false for $n = 1$.

7 Here we take the $L^1$ norm for the matrix *Z* simply for convenience; except for the developments of Sect. 10.5, any norm will do.

8 In principle, we know that $(\alpha _N)$ has a weak-$*$-convergent *subnet*, which is not necessarily a sequence. However, since $C_0([0, \infty ))$ with the supremum norm is separable, the closed unit ball in $M([0, \infty ))$ is second countable [16, p. 171, Exercise 50], and hence the sequence $(\alpha _N)$ has a weak-$*$-convergent subsequence.

9 This is true for the Bessel function [47, Chapter XV].

10 This follows from Farkas’s Lemma. The vectors above form the rows of the constraint matrix of the finite linear-inequality system (49).
